# Vibrational Damping Reveals Vibronic Coupling in Thermally Activated Delayed Fluorescence Materials

**DOI:** 10.1021/acs.chemmater.0c03783

**Published:** 2021-04-28

**Authors:** Matthias Hempe, Nadzeya A. Kukhta, Andrew Danos, Mark A. Fox, Andrei S. Batsanov, Andrew P. Monkman, Martin R. Bryce

**Affiliations:** † Chemistry Department, Durham University, South Road, Durham DH1 3LE, U.K.; ‡ Physics Department, Durham University, South Road, Durham DH1 3LE, U.K.

## Abstract

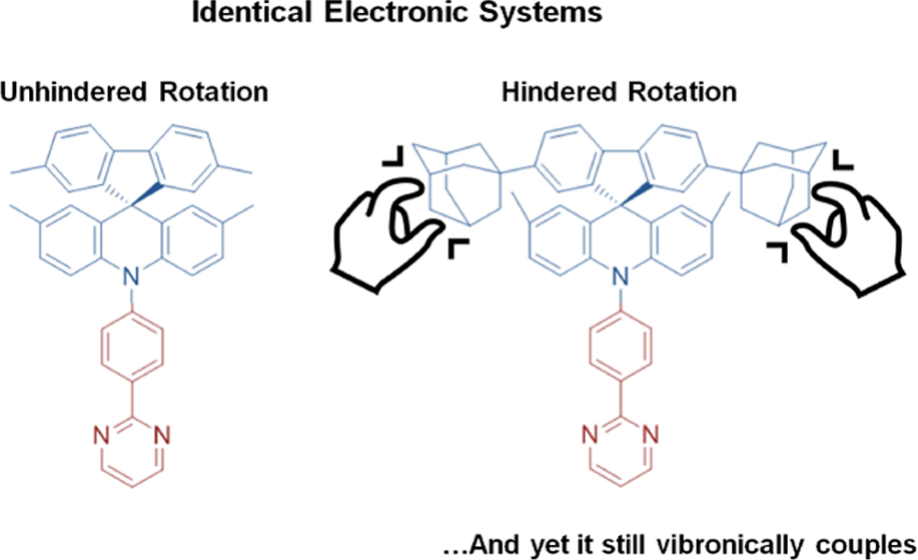

We investigate a series of D–A molecules consisting of spiro[acridan-9,9′-fluorene] as the donor and 2-phenylenepyrimidine as the acceptor. In two of the materials, a spiro center effectively electronically isolates the D unit from (consequently) optically innocent yet structurally influential adamantyl side groups. In a third material, adamantyl groups attached directly to the acceptor strongly influence the electronic properties. Steady-state and time-resolved photophysical studies in solution, Zeonex polymer matrix, and neat films reveal that the substituents impact the efficiency of vibronic coupling between singlet and triplet states relevant to reverse intersystem crossing (rISC) and thermally activated delayed fluorescence (TADF), without significantly changing the singlet–triplet gap in the materials. The adamantyl groups serve to raise the segmental mass and inertia, thereby damping intramolecular motions (both vibrational and rotational). This substitution pattern reveals the role of large-amplitude (primarily D–A dihedral angle rocking) motions on reverse intersystem crossing (rISC), as well as smaller contributions from low-amplitude or dampened vibrations in solid state. We demonstrate that rISC still occurs when the high-amplitude motions are suppressed in Zeonex and discuss various vibronic coupling scenarios that point to an underappreciated role of intersegmental motions that persist in rigid solids. Our results underline the complexity of vibronic couplings in the mediation of rISC and provide a synthetic tool to enable future investigations of vibronic coupling through selective mechanical dampening with no impact on electronic systems.

In recent years, purely organic light-emitting materials displaying thermally activated delayed fluorescence (TADF) have proven their high potential for optoelectronic applications. Upon electrical excitation, these materials demonstrate their capability of overcoming the 25% efficiency limitation of purely fluorescent materials imposed by spin statistics, paving the way for future high-efficiency lighting and display applications.^1^ While the main application of TADF materials is in organic light-emitting diodes (OLEDs),^2−4^ other applications include fluorescence sensing and imaging,^5^ optical temperature sensing,^6^ and catalysis.^7^

While the exact photophysical processes involved in TADF continue to be the focus of considerable experimental and computational research,^8^ the established general understanding involves the thermally facilitated upconversion and spin-flip of excited triplet states to excited singlet states in a key step known as reverse intersystem crossing (rISC). In order for this step to be efficient, emitting molecules should exhibit minimal energy splitting (Δ*E*_ST_) between the involved excited states. This electronic property can be engineered in molecules containing electron donor (D) and acceptor (A) moieties on spatially separated segments, leading to a minimal overlap of the frontier molecular orbitals. This design results in excited states of intramolecular charge-transfer (CT) character, which were found to be a crucial part of the TADF mechanism. Since spin–orbit coupling (SOC) of singlet and triplet states of the same character is formally forbidden, recent studies of the TADF mechanism instead indicate that a coupling of excited triplet charge-transfer states (^3^CT) with localized excited triplet states (^3^LE) allows for rapid rISC toward excited singlet charge-transfer states (^1^CT).^9,10^ This kind of coupling requires a small energy difference between the states involved, as well as the existence of coupling vibrational modes (primarily D–A dihedral angle rocking), which further lower the energetic activation barrier of the rISC.^11−13^

In this context, successful applications of Arrhenius models to the temperature dependence of experimental rISC rates demonstrate the high relevance of Δ*E*_ST_ to rISC and TADF performance. Nonetheless, the underlying two-state Arrhenius model is not sophisticated enough to fully explain the rISC mechanism. A very recent study correctly notes that the model neglects “the underlying physics encoded in the empirical preexponential factor of the Arrhenius equation (e.g., the vibrational density of states, spin–orbit coupling, and other nonadiabatic effects),” which are vital in promoting rISC, especially for larger Δ*E*_ST_ materials.^14^ Indeed, it was observed that despite very similar energy gaps in a series of D–A–D molecules, severely reducing the dihedral twisting motion between the donor and acceptor segments by substituent effects, or changing the excitonic character (CT versus LE), can lead to large variations of the rISC rate of the respective materials.^15,16^ This former result indicates a strong correlation between the TADF efficiency and the intramolecular (intersegmental) freedom of movement, while the latter demonstrates that other molecular factors encompassed by the Arrhenius preexponential factor can, in some cases, be just as important as Δ*E*_ST_.

While these previous results have shown that substituents on the D and/or A subunits can have an important role in the TADF and rISC processes, suitable molecular structures to systematically and directly investigate the role of vibrational modes on the rISC mechanism have not been reported. The main challenge in this respect lies in the design of directly comparable TADF molecular systems, where the D–A intersegmental or vibrational motion is modulated without otherwise impacting the shape of the energy surface, i.e., maintaining the energy states and gaps involved in the coupling process, so that direct comparison can be made.

Furthermore, relevant low-frequency intersegmental motions (in the range of 100 meV; 800 cm^–1^) in D–A compounds can be divided into “major” dihedral rotational displacements of large amplitude, as well as more subtle, low amplitude, or localized intramolecular movements such as rocking, bending, or stretching (breathing) mode vibrations. While major torsional motions are often invoked in the discussion of TADF, fully unrestricted motion is available only in the gas phase or in the solution state. The latter low-amplitude intersegmental motions are often neglected despite these still being potentially active in solid state relevant to thin-film OLED devices. Hence, these subtle intramolecular motions may have an unrecognized impact on the rISC process and the overall TADF efficiency. However, the isolated contribution of such motion toward rISC and TADF has not been studied until now, as methods to selectively dampen large-amplitude motions without changing the underlying electronic system have not been reported.

As a launching point toward this deeper understanding, pyrimidine-based donor–acceptor compounds have been reported as high-efficiency TADF emitters for vacuum-processed OLEDs, with external quantum efficiencies exceeding 30%.^17,18^ Due to their intrinsically high triplet energies and weaker electron-accepting properties than widespread 1,3,5-triazine derivatives, pyrimidines can be used for wide-band-gap donor–acceptor materials and offer a good basis for the design of blue-emitting TADF molecules.^19−22^ Similarly, spirofluorene-substituted acridine donor segments have been used to construct materials with high photoluminescence quantum yields and low singlet–triplet energy gaps (Δ*E*_ST_).^19,23,24^ The spirofluorene moiety is accessible for derivatization and hence is an excellent platform for material development.^25^ In this context, the sp^3^-nature of the spiro connection allows for the attachment of varying substituents on the fluorene site, which are strongly decoupled from the electronic system of the acridine donor segment.^26^ This feature is crucial for probing the physical properties of charge transfer-based excited states, without introducing any differences in the electronic system that otherwise confound direct comparison.

In this paper, the influence of different vibrational motions on TADF performance is investigated. Inspired by classical mechanics, we report a comprehensive structure–property investigation of spiro[acridan-9,9′-fluorene]–2-phenylene-pyrimidine-based D–A compounds (Figure 1) bearing optically innocent, yet structurally influential, adamantyl side groups. The attachment of these groups onto the lateral positions of a TADF parent compound^19^ leads to a negligible impact on the electronic structure of the original system while raising the segmental molecular mass and inertia (both vibrational and rotational). Despite their remote point of attachment with respect to the intersegmental C–N bond, these side groups lead to a damping of vibrational modes due to the rigidity of the overall structure. The effect of these altered intramolecular dynamics on the TADF properties was observed through steady-state and transient photophysical measurements, both in the solution and in the solid state, highlighting the fundamental process of vibronic coupling in TADF materials. Therefore, although the rotational potential energy surfaces are the same, the dampened material will explore this potential more slowly, corresponding to less active vibrations and diminished rISC. Combined with theoretically predicted structural and electronic properties, and consideration of molecular packing, the photophysical data also reveal contributions of small-amplitude vibrational motion to electronic properties relevant to rISC. The comparisons described in this work are underpinned by the ability of adamantyl substitution on spiro-linked D–A compounds to modify the relaxation pathways without altering the electronic structure, thus giving deeper insights into the working principles of TADF emitters.

**Figure 1:**
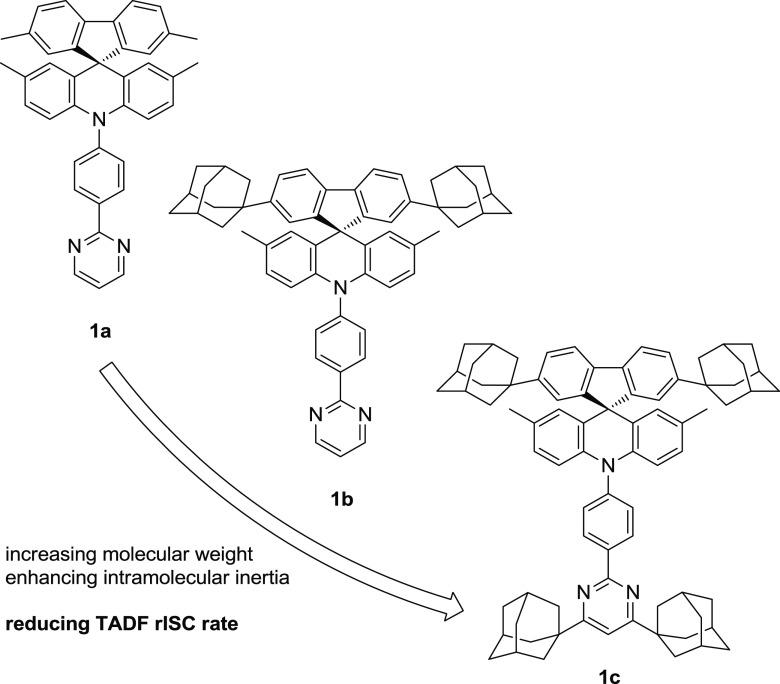
Concept and molecular design presented in this work.

## Experimental Section

### General Experimental Details

Chemicals and reagents were purchased from commercial providers and used without further purification. Detailed experimental procedures and characterization data are given in the Supporting Information. Solvents were dried using an Innovative Technology solvent purification system and were stored in ampules under argon. Moisture and/or air-sensitive experiments were conducted using thoroughly dried glassware under an argon atmosphere. ^1^H NMR spectra were recorded on Bruker AV400, Varian VNMRS 600, and 700 spectrometers operating at 400, 600, and 700 MHz, respectively. ^13^C NMR spectra were recorded on the same instruments at 100, 150, and 175 MHz. Chemical shifts (δ) in ^1^H NMR and ^13^C NMR spectra were reported in ppm and were referenced against the residual solvent signal as reported in the literature.^27^ The fine structure of proton signals was specified as s (singlet), d (doublet), t (triplet), q (quartet), m (multiplet), and br (broad). Flash chromatography was carried out on silica gel 60 (40–63 μm) purchased from Fluorochem. High-resolution mass spectroscopy was carried out on a Waters LCT Premier XE using ASAP ionization with TOF detection. Samples were analyzed directly as solids. Melting points were carried out on a Stuart SMP40 machine with a ramping rate of 4 °C min^–1^. Videos were replayed manually to determine the melting point or melting range. Single-crystal X-ray experiments were performed on a Bruker 3-circle D8 Venture diffractometer with a PHOTON 100 CMOS area detector, using Mo Kα radiation (λ = 0.71073 Å) from an Incoatec IμS microsource with focusing mirrors. Crystals were cooled using a Cryostream (Oxford Cryosystems) open-flow N_2_ gas cryostat. The data were processed using APEX3 v.2016.1-0, reflection intensities integrated using SAINT v8.38A software (Bruker AXS, 2016) and scaled using SADABS-2016/2 program.^28^ The structures were solved by dual-space intrinsic phasing method using SHELXT 2018/2 program^29^ and refined by full-matrix least squares using SHELXL 2018/3 software^30^ on the OLEX2 platform.^31^ Cyclic voltammetry was conducted using an electrochemical cell comprised of a platinum electrode with a 1 mm diameter of the working area as a working electrode, an Ag/AgCl electrode as a reference electrode, and a platinum coil as an auxiliary electrode. Cyclic voltammetry measurements were conducted at room temperature at a potential rate of 50 mV s^–1^ and were calibrated against the internal ferrocene/ferrocenium redox couple. The measurements were conducted in ca. 1.0 mM concentrations of all compounds in 0.1 M solutions of TBAPF_6_ in *N,N*-dimethylformamide (abs.). Steady-state absorption and emission spectra were measured using a double beam Shimadzu UV-3600 UV/VIS/NIR spectrophotometer and a Horiba Jobin Yvon Fluorolog-3 spectrofluorometer. Time-resolved measurements were performed using a spectrograph and a Stanford Computer Optics 4 Picos ICCD camera, where samples were excited with a Nd:YAG laser (EKSPLA), 10 Hz, 355 nm or using a nitrogen laser, 10 Hz, 337 nm, for power dependence measurements.

## Results and Discussion

### Molecular Design and Synthesis

The synthesis of the materials **1a–c** used in this study (Figure 1) is based on the modular substitutions of the respective acridine donor and phenyl–pyrimidine acceptor segments. 5,5′-Dimethyl-substituted acridine–pyrimidine-based TADF molecules have been shown to emit at a wavelength of 448 nm in toluene solution,^19^ while the intrinsic fluorescence wavelength of the fluorene structure is onset at 320 nm (3.8 eV),^32^ with a lowest excited triplet state energy of 3.1 eV. Hence, the high-energy excited states of the fluorene system cannot quench excitons from the acridine–pyrimidine singlet or triplet. Furthermore, the electron-rich 2- and 7-positions of the fluorene precursor allow for attachment of bulky adamantyl groups via Friedel–Crafts alkylation reactions (see the Supporting Information). These groups install relatively high molecular mass in the molecule at sites that are well displaced from the axis of D–A bond rotation, while having no impact on the D–A charge-transfer system in the case of **1b** (see optical results below). In a similar approach, bulky adamantyl groups can be attached at the pyrimidine site through the use of Minisci reactions. However, without the electronic decoupling afforded by the spiro linkage, this modification was found to decrease the acceptor strength, precluding direct comparison of optical properties for the resulting material **1c**. Full descriptions of the synthetic procedures and structural characterization (HRMS, ^13^C and ^1^H NMR, and VT NMR) are presented in the SI.

#### X-ray Structures

**Figure 2:**
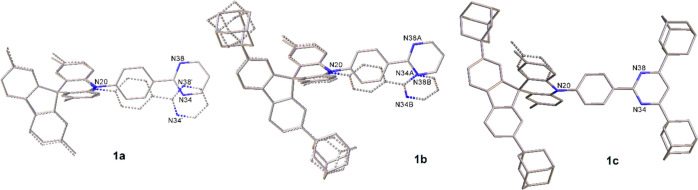
X-ray molecular structures of compounds **1a** in pure (solid) and solvated (dashed) crystal, **1b** (showing overlay of two independent molecules), and **1c** (showing the disorder). H atoms are omitted for clarity.

Compound **1a** crystallized in pure form and as a **1a**·CD_2_Cl_2_ solvate, the latter with two host molecules and two disordered CD_2_Cl_2_ molecules per asymmetric unit. Compound **1b** also crystalized as a CD_2_Cl_2_ solvate, its asymmetric unit comprising four host molecules and three CD_2_Cl_2_ molecules. Derivative **1c** crystalized as **1c**·3CD_2_Cl_2_ solvate, with intensely disordered solvent. The conformations of independent molecules show substantial variations (Figure 2) but are broadly similar (see the Supporting Information, Tables S1A,B): the acridine system is folded along the N···C(sp^3^) vector (angle θ), the acridine N atom has nearly planar geometry (the sum of bond angles >355°), and the phenylene ring is nearly perpendicular to the acridine plane (angle τ) and nearly coplanar with the pyrimidine ring (angle φ). The most prominent conformational difference is the large tilt of the *N*-phenylenepyrimidine vector (e.g., indicated by its angle with the long axis of the fluorene moiety, ψ), between two crystal forms of **1a** and between four independent molecules of **1b**.

## Calculations

Detailed hybrid density functional theory (DFT) and time-dependent DFT (TD-DFT) computations were performed on all three compounds **1a–c** to predict the reported photophysical data in this study. Ground-state (S_0_) geometry optimizations of **1a** with various self-consistent field (SCF) methods (HF, pure DFT, hybrid-DFT) revealed geometries where the folding of the acridine is absent (Figure S30). This contrasts with acridine folding present in the experimental geometries determined by X-ray crystallography. The fully electron-correlated Møller–Plesset perturbation theory (MP2) gave a minimum geometry in agreement with the experimental geometries of **1a** (Figure S31). The transition state geometry of **1a** at MP2 where the acridine is planar (as observed with SCF methods) is estimated to be only 0.015 eV higher in energy. However, the post-SCF method MP2 could not be used here for photophysical predictions due to limited computing resources available. Given that the energy differences between geometries with and without acridine folding are very small, it is assumed here that geometries with planar and folded acridines are present in solutions of **1a**–**1c** at 20–25 °C.

**Figure 3:**
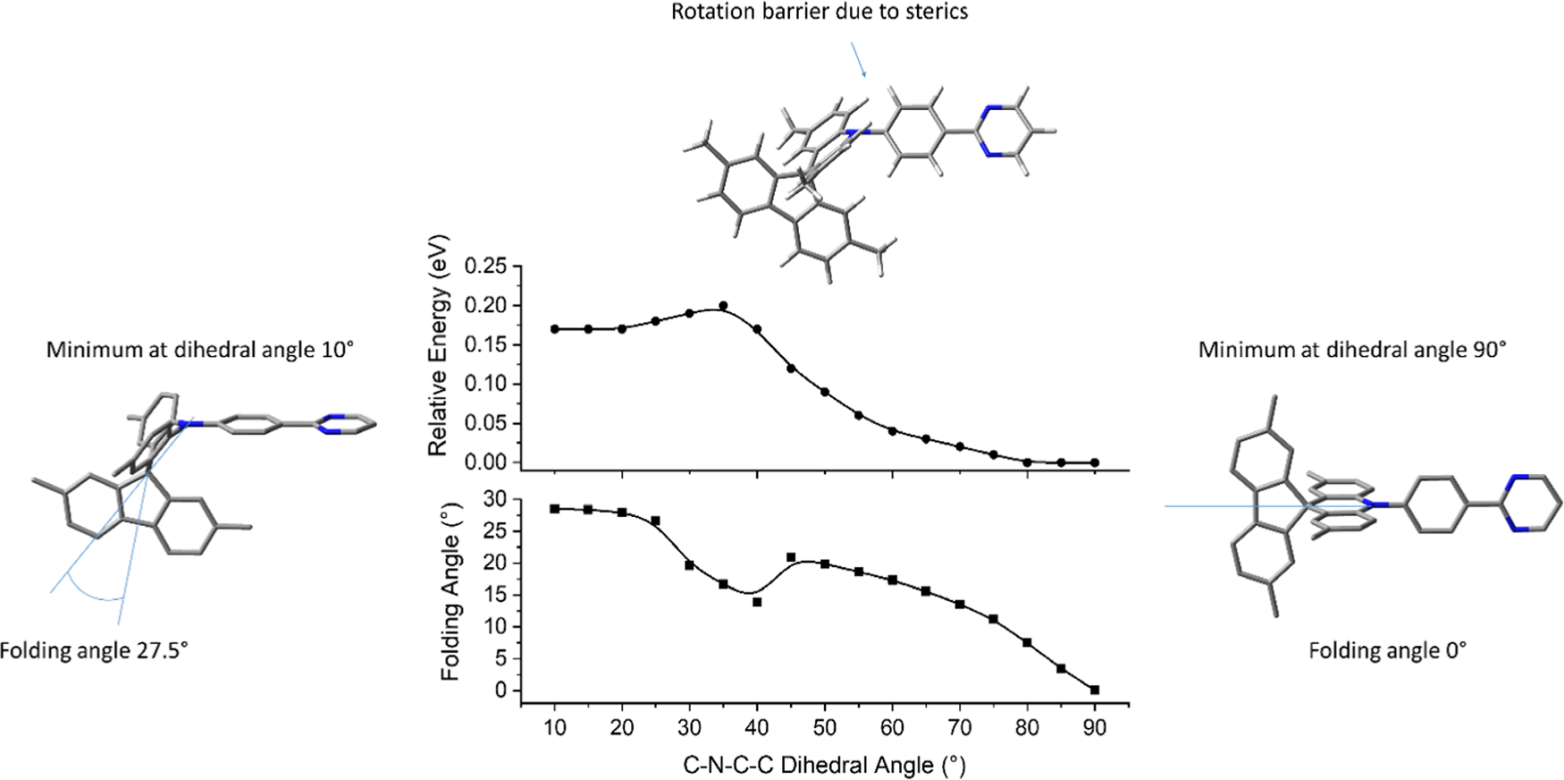
Plots of relative energy and acridine folding angle (ω) against the fixed C–N–C–C dihedral angle (τ) related to D–A bond rotation for **1a** in the S_0_ ground state at CAM-B3LYP/6-31G(d).

Of the SCF methods available, the hybrid-DFT CAM-B3LYP functional is appropriate for donor–acceptor (D–A) molecules, and their charge-transfer (CT) properties thus are employed here for **1a**–**1c**. Experimental geometries of **1a**–**1c** have a range of C–N–C–C dihedral angles between 73.4 and 89.5° related to the rotation about the D–A N–C bond. Here, optimized geometries with the C–N–C–C dihedral angle (τ) constrained at 5° intervals show that the folding of the acridine (ω) increases rapidly from τ = 70° to 90° with energy differences of merely 0.015 eV for **1a**–**1c** (Figures 3 and S32). All three D–A molecules have the same energy trends when D–A bond rotations take place; thus, the adamantyl groups in groups in **1b** and **1c** have no steric influence on the D–A bond rotations.

Alternative minima were located for **1a**–**1c** with C–N–C–C dihedral angles at around 10° and are 0.15–0.17 eV higher in energies compared to corresponding geometries with angles at 90° (Figures 3 and S32). The rotation energy barriers between the two minima arise from unfavorable steric interactions between phenylene and folded acridine at 35–40°. Orthogonal conformers with C–N–C–C dihedral angles of 70–90° and planar/folded acridine groups are predicted to exist in standard experimental conditions for **1a**–**1c**. The lowest frequencies calculated for the most stable minimum of **1a** were 3.0 cm^–1^ for the acridine folding, 20.4 cm^–1^ for the twisting of the D–A N–C bond, and 21.7 cm^–1^ for the inversion at acridine nitrogen, which are in agreement with conformers found experimentally.

The frontier orbitals for **1a** and **1b** and their energies are essentially identical which demonstrate that the bulky adamantyl groups at the donor group are electronically innocent (Figure S33). As expected for D–A molecules, the lowest unoccupied molecular orbital (LUMO) is located on the phenylenepyrimidine moiety and the highest occupied molecular orbital (HOMO) is at the acridine unit. Any difference in the photophysical data between **1a** and **1b** must therefore be influenced by steric interactions from the bulky adamantyl groups. A slightly different picture applies for **1c** where the adamantyl groups at the pyrimidine in **1c** influence the LUMO with the LUMO energy 0.27 eV higher than LUMO energies for **1a** and **1b**, whereas the HOMO energy in **1c** is only 0.05 eV higher than HOMO energies for **1a** and **1b**. These MO energy trends are in excellent agreement with observed cyclic voltammetry (CV) data, where the oxidation potentials for **1a**–**1c** are all within 0.02 V and the reduction potential of **1c** is 0.14 and 0.19 V more negative than **1a** and **1b**, respectively (Table S5 and Figures S58–S60). The HOMO–LUMO energy gaps estimated by CV data are 2.78, 2.75, and 2.93 eV for **1a**, **1b**, and **1c**. A subtle difference in the LUMOs between **1a** and **1c** is the relative % MO contributions of phenylene:pyrimidine units in the acceptor group with 41:57 for **1a** and 46:52 for **1c** (Figure S33).

Predicted absorption spectra by time-dependent DFT (TD-DFT) computations on minima of **1a**–**1c** gave the expected charge-transfer (CT) transitions as the lowest energies. However, the S_0_ → S_1_ transitions have zero oscillator strengths which are expected from zero orbital overlaps between HOMO and LUMO.^9^ As other conformers are assumed to be present with C–N–C–C dihedral angles (τ) of 70° or higher with folded acridine units, simulated absorption spectra on conformers of **1a** reveal that the oscillator strength of the charge transfer increases on decreasing the dihedral angle τ (Figure S34). Simulated absorption spectra for **1a–1c** using conformers with C–N–C–C dihedral angles of 80° show that weak CT bands with **1c** blue-shifted with respect to **1a** and **1b** in line with their predicted and experimental HOMO–LUMO energy gaps (Figure S35).

The primary focus of our photophysical investigation is the luminescence properties, especially TADF, of the D–A molecules **1a–1c**. Unlike absorption spectra and optimized ground-state geometries, emission spectra arise from excited states and can be best modeled from optimized excited-state geometries. The optimized S_1_ excited-state geometries for **1a**–**1c** overall resemble the corresponding optimized geometries at S_0_ with C–N–C–C dihedral angles (τ) of 90° and planar acridine groups (Figure S36). There are significant geometric changes at the planar phenylenepyrimidine units so reorganization energies are substantial from S_0_ to S_1_ geometries at 0.24, 0.24, and 0.26 eV for **1a**, **1b**, and **1c**, respectively (Figure S37). It is important to note that these reorganization energies, identical for **1a** and **1b**, are calculated in the absence of an explicit medium.

**Figure 4:**
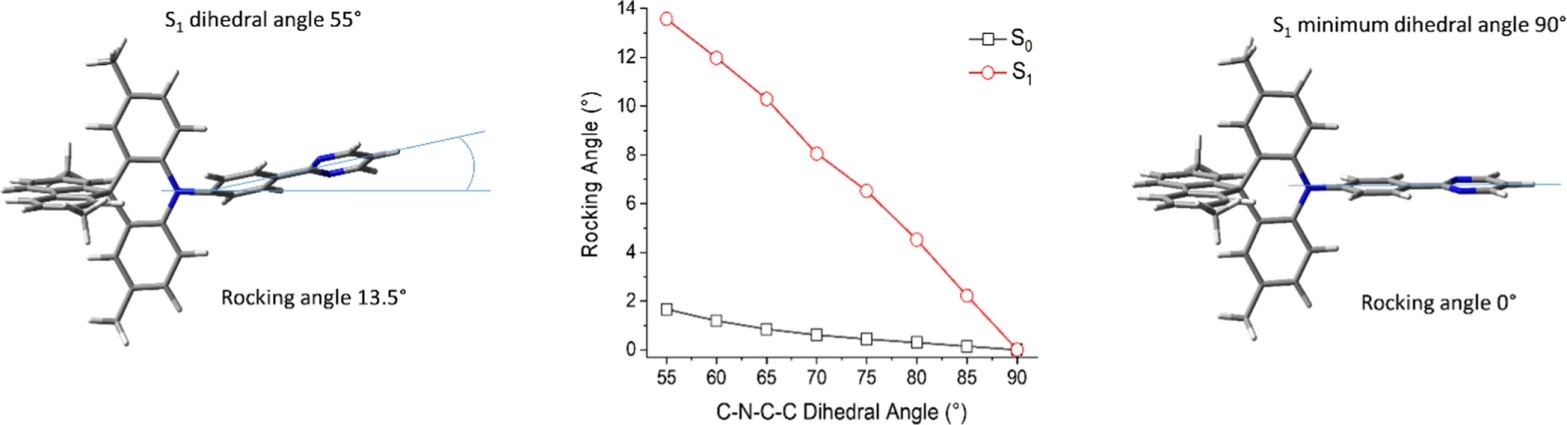
Comparison of the rocking angle against the fixed C–N–C–C dihedral angle (τ) related to D–A N–C bond rotation for **1a** in the S_0_ ground and S_1_ excited states at CAM-B3LYP/6-31G(d).

Changes in the S_1_ excited-state geometries when bond rotation takes place at the D–A N–C bond were examined for **1a** using fixed C–N–C–C dihedral angles (τ) between 55 and 90° at 5° intervals. Instead of the acridine folding as the main geometric effect found in the S_0_ ground state, the bending (rocking) of the acceptor group with respect to the D–A N–C bond was the major change demonstrated in the S_1_ excited state (Figure 4). The relative energies between S_0_ ground and S_1_ excited states on D–A N–C bond rotations are remarkably similar despite the different geometry changes on D–A N–C bond rotation, and the potential energy surface for this molecular motion is identical for **1a** and **1b** in the S_1_ state (Figure S38). The lowest frequencies calculated for the most stable minimum of **1a** at the S_1_ excited state were 4.1 cm^–1^ for the acridine folding, 12.3 cm^–1^ for the rocking of the acceptor at the N–C bond, 26.2 cm^–1^ for the twisting of the D–A N–C bond, and 21.7 cm^–1^ for the inversion at acridine nitrogen in agreement with the structural changes described in Figure 4.

Charge-transfer emissions are solvatochromic and different emission maxima from the D–A systems **1a**–**1c** are expected from solutions and solids of different polarities. The state-specific corrected linear response polarizable continuum solvation model (cLR-PCM)^33,34^ is applied to optimized S_1_ geometries with argon, toluene, *ortho*-dichlorobenzene (*o*-DCB), dichloromethane (DCM), and acetonitrile as solvents (Table S2). The cLR-PCM S_1_ (^1^CT) energies align with known polarities and dielectric constants of the solvents. One exception is that DCM has a higher solvent polarity index value but a lower dielectric constant value than *o*-DCB. TADF is considered to be present when the S_1_ and T_1_ energies are close together to promote reverse intersystem crossing (rISC). Favorable spin–orbit couplings (SOC) between the charge-transfer (^1^CT) singlet excited state and calculated excitation energies are therefore similar for DCM and *o*-DCB.

**Figure 5:**
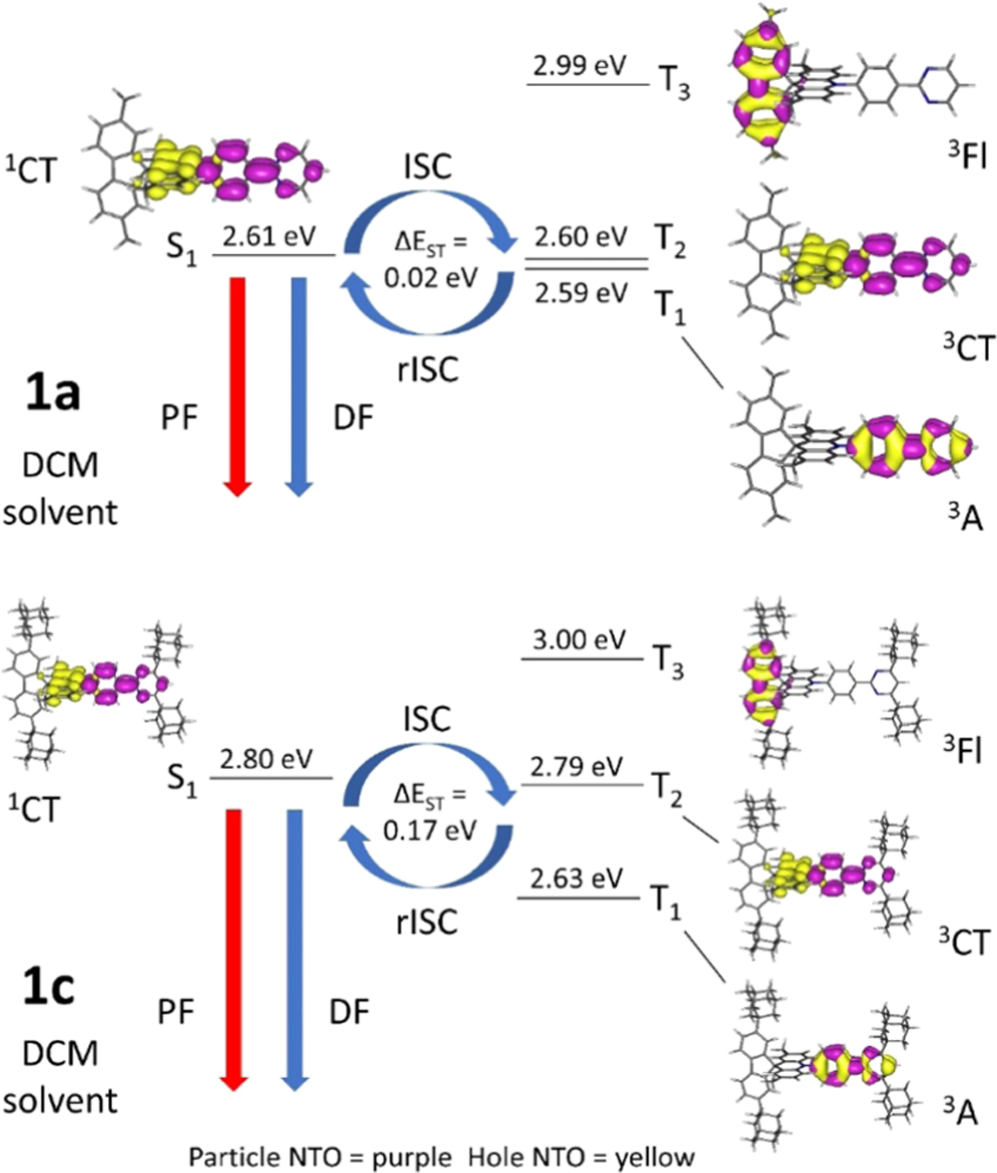
Energy diagrams illustrating TADF with natural transition orbitals (NTOs) for each state on optimized S_1_ excited-state geometry of **1a** and **1c** from TD-DFT computations with the solvation model cLR-PCM using DCM as a solvent. PF = prompt fluorescence, DF = (thermally activated) delayed fluorescence, ISC = intersystem crossing, RISC = reverse intersystem crossing, ΔE_ST_ = S_1_ energy – T_1_ energy, ^1^CT = singlet charge-transfer state, ^3^CT = triplet charge-transfer state, ^3^Fl = local triplet excitation state at the fluorene donor unit, and ^3^A = local triplet excitation state at the acceptor unit. Contours in NTOs are drawn at ± 0.04 (e/bohr^3^)^1/2^.

The important energy gaps between S_1_ and T_1_ energies decrease on increasing solvent polarity for **1a**–**1c** in all solvents explored here. The T_1_ state is the local excitation (^3^LE) except for **1a** and **1b** in acetonitrile, where the T_1_ state is charge transfer (^3^CT). Computed excited-state data for **1b** are essentially identical to **1a** reflecting zero electronic influence by the bulky adamantyl groups at the donor unit on the predicted emission properties. The S_1_(^1^CT) and T_1_(^3^LE) energies are close together for **1a** and **1b** in DCM and *o*-DCB solutions with only 0.01–0.02 eV differences and would result in efficient TADF. Figure 5 shows the excited states with natural transition orbitals (NTOs) expected to be involved in the TADF process in **1a** and **1c** by TD-DFT computations with DCM as solvent. For **1c** in DCM and *o*-DCB solutions, the S_1_(^1^CT) and T_1_(^3^LE) energies are not as close with a difference of 0.17 eV and thus would not be expected to have TADF as efficient as for **1a** and **1b**. **Table S3** lists the nature and energies of relevant excited states for three compounds.

The singlet states (S_1_) in **1a**–**1c** from the calculated NTOs have essentially pure charge-transfer character (95–96%), indicating that solvatochromism is expected in emissions from solutions of different polarities (Table S3). The lowest energy triplet states, T_1_, T_2_, and T_3_, in all compounds are a local acceptor state (^3^A), a pure charge-transfer state (^3^CT), and a local fluorenyl state (^3^Fl) respectively. The local triplet states (^3^A) close in energies (ΔE_ST_ 0.01–0.36 eV in solvents, with the S_1_ charge-transfer states) facilitate TADF as spin–orbit couplings (SOCs) occur between singlet and triplet states of different orbital characters with SOC matrix elements (SOCME) at 0.66–0.70 cm^–1^ (Table S4). Spin–orbit couplings between a singlet charge-transfer state and a triplet charge-transfer state are forbidden with SOCME calculated at 0.00 cm^–1^. The vibronic couplings between close low-energy triplet states (ΔE_TT_ 0.01–0.16 eV, Table S3) are also assumed to aid the rISC process for TADF.

Zeonex (solid-state film) is relatively nonpolar and thus predicted to have emission properties closer to toluene and argon gas. With this in mind, **1a**–**1c** in Zeonex may not have efficient TADF compared to that of **1a**–**1c** in DCM and *o*-DCB solutions due to the larger predicted S_1_(^1^CT) and T_1_(^3^LE) energy gaps of 0.23–0.36 eV in toluene and 0.37–0.47 eV in argon (Table S2).

The optimized T_1_ geometries differ from optimized S_0_ and S_1_ geometries considerably at CAM-B3LYP/6-31G(d) with the C–N–C–C dihedral angles at the D–A N–C bond at 56.8, 56.8, and 56.6° for **1a**, **1b**, and **1c**, respectively. The ISC/rISC reorganization energies are predicted from optimized S_1_ and T_1_ geometries to be 0.29, 0.29, and 0.30 eV for **1a**, **1b**, and **1c** in this order. These calculated values uphold the previous trend of **1a** and **1b** showing near-identical electronic properties.

## Photophysical Properties

### Zeonex Solid Solutions

**Figure 6:**
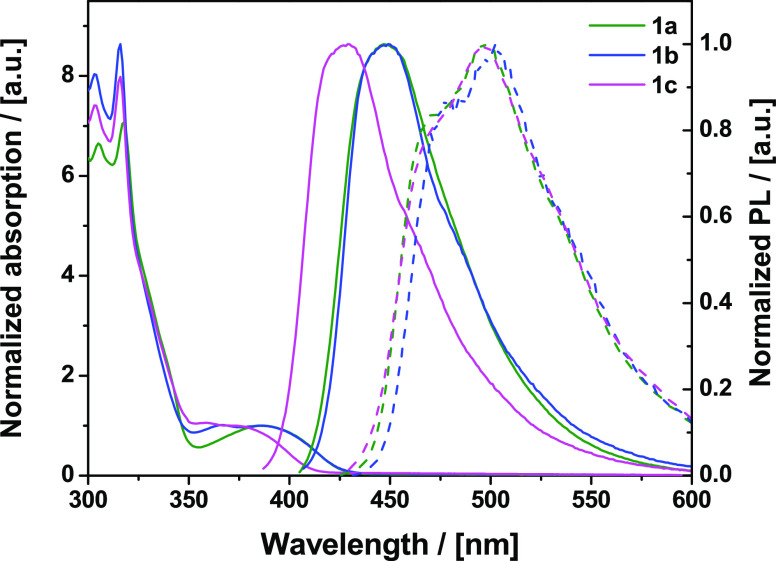
Steady-state absorption and photoluminescence spectra (solid lines) along with the 80 K phosphorescence spectra (delay 10 ms, dashed lines) of 5% w/w Zeonex films of **1a**, **1b**, and **1c**.

The UV–vis absorption of molecular dispersions of the target compounds in Zeonex (5% w/w films drop-cast from toluene on quartz/sapphire substrates) extends to 440 nm (Figure 6). As the D and A units absorb only below 350 nm,^17,32,35−38^ the new weak bands at λ_max_ 375–385 nm arise from direct excitation of the “whole molecule” CT band—as expected from the simulated UV–vis spectra (Figures S34 and S35**)**. Further evidence of this assignment is the blue shift of these absorption bands with increasing solvent polarity (Figure S39). The CT character of the pyrimidine derivatives’ S_1_ state is also confirmed by the positive solvatochromism in emission (Figure S40). While the low-energy bands in absorption and emission spectra of **1a** and **1b** almost coincide, both UV–vis and photoluminescence (PL) spectra of tetra-adamantyl-substituted **1c** are significantly blue-shifted by ca. 20 nm (∼0.15 eV) in the same host/solvent. The blue shift is expected as the electronically distinct acceptor of **1c** leads to the weakest CT character as predicted by the DFT calculations and cyclic voltammetric data (Table S5 and Figures S58–S60). We do not believe that the blue shift for **1c** is due to packing effects as the molecules are at low concentration (5% w/w) in the host film. This is supported by similar observations in dilute solution (Figures S39 and S40) where packing effects can be strongly excluded from consideration. Structured low-temperature phosphorescence spectra (PH) in Zeonex (Figure 6) reveal that all of the compounds feature the same emissive triplet state, presumably stemming from the acridine moiety.^35^ Of note, the photoluminescence quantum yields (PLQYs) of **1a** and **1b** in Zeonex are also almost identical (71 and 66%, respectively), again indicating a very similar electronic system. In contrast, **1c** has a significantly different, lower, PLQY of 38%, consistent with an altered electronic system due to the weaker acceptor.

**Figure 7:**
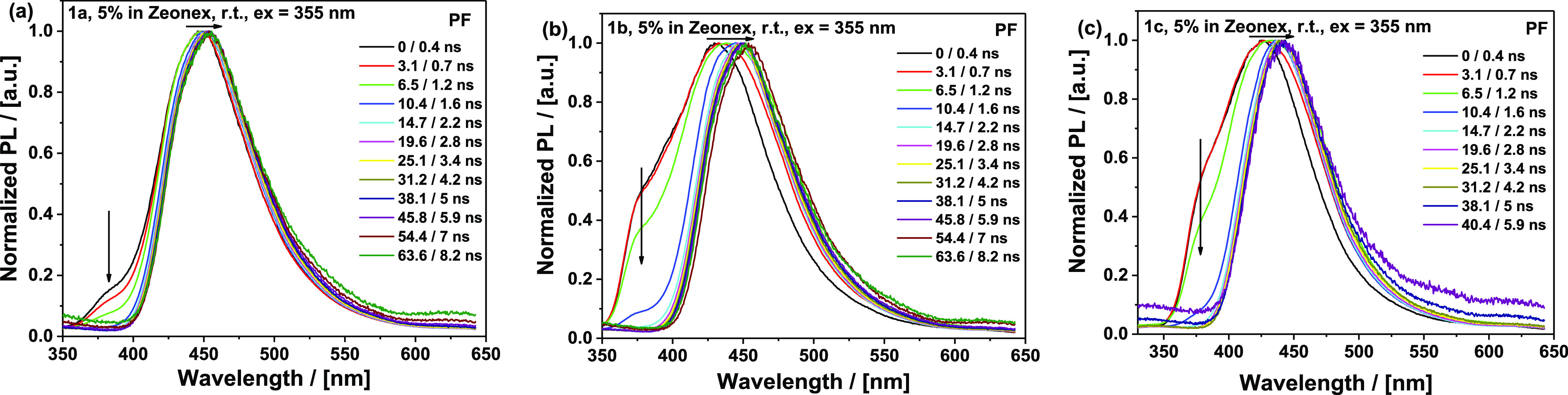
Time-resolved prompt photoluminescence spectra of 5% Zeonex films of (a) **1a**, (b) **1b**, and (c) **1c** at specified delay/integration times (excitation 355 nm, Nd-YAG laser, room temperature).

**Figure 8:**
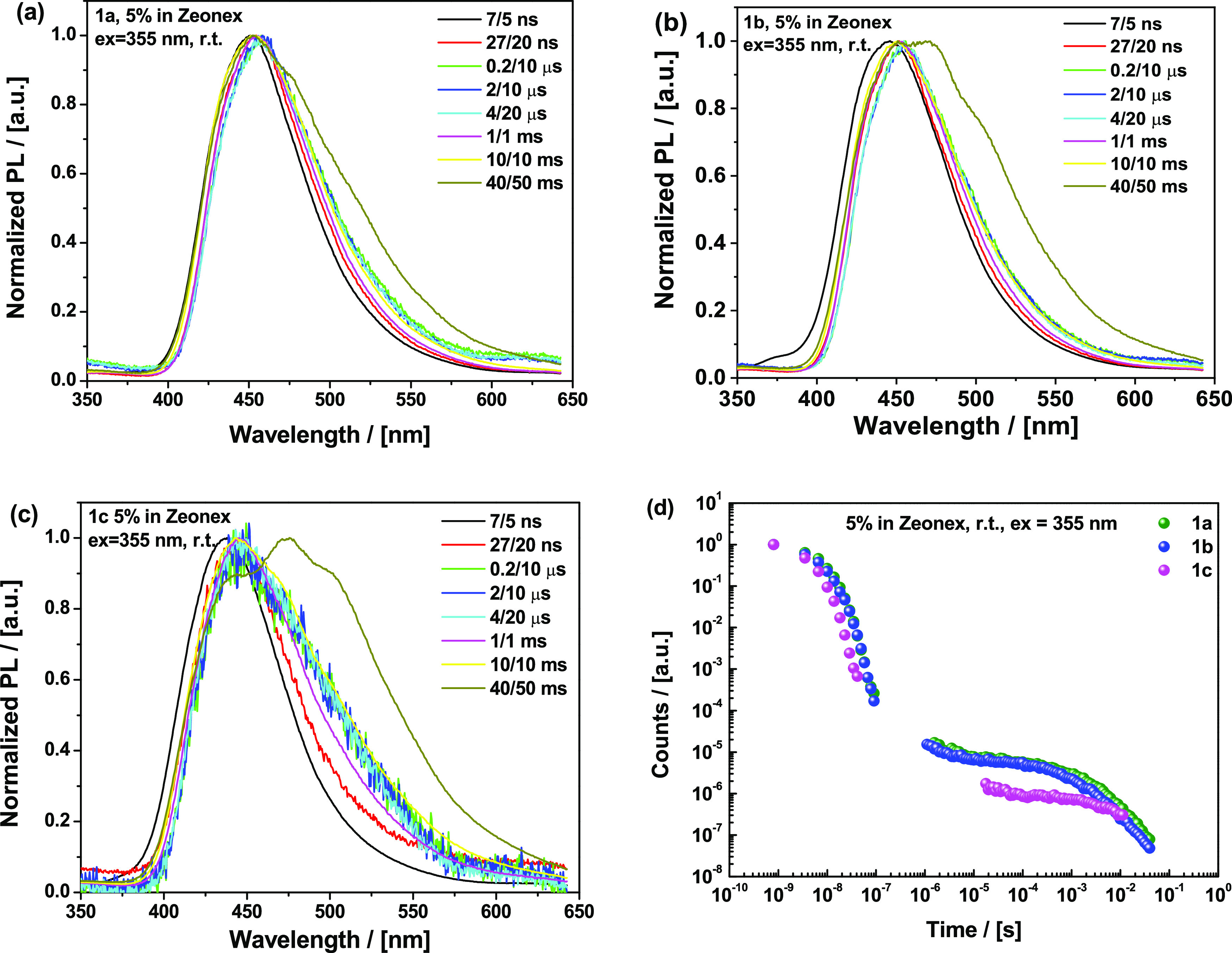
Time-resolved photoluminescence spectra of 5% Zeonex films of (a) **1a**, (b) **1b**, and (c) **1c** recorded at room temperature at specified delay/integration times (excitation 355 nm, Nd-YAG laser); (d) decay curves.

As the molecular design of the compounds in this work was inspired by a reported TADF emitter,^19^ time-resolved spectroscopic data were collected for the Zeonex films to study the effect of the D–A adamantyl substitution on the TADF performance. Room-temperature time-resolved emission spectra in Zeonex (Figures 7 and 8) reveal important differences between the compounds. Even though **1a** and **1b** feature nearly identical steady-state photophysics, comparison of the time-resolved prompt fluorescence (PF) (Figure 7) points at the key role of adamantyl substitution in the conformational dynamics and electron transfer (ET) leading to CT state formation.^39^ Compound **1a** with lighter methyl substituents on the donor (and an unencumbered acceptor) is capable of faster D–A angle relaxation and associated CT stabilization, showing only a low-intensity LE-origin peak (at ca. 380 nm, from direct excitation of the D or A fragments) in its early PF. This LE PF emission component corresponds to donor or acceptor emission occurring on faster timescales than the slow ET in these highly decoupled, perpendicular D–A systems. Furthermore, after the formation of the CT state, PF emission shifts by 7 nm (∼50 meV) over 63 ns until reaching a relaxed conformation (λ_max_ = 452 nm). In contrast, **1b** with heavier adamantyl moieties on the donor reveals a higher intensity shoulder at 380 nm and a 23 nm (∼160 meV) shift over 63 ns until it reaches the same CT emission as **1a** (λ_max_ = 452 nm) (confirmed by the maps of normalized emission spectra, Figure S42). With an additionally hindered acceptor leading to yet slower ET, **1c** features an even higher intensity of the LE peak (380 nm) and a shift of 17 nm (∼120 meV) over the time needed for CT (λ_max_ = 441 nm) stabilization, although its weaker acceptor strength (and therefore different driving force for the ET step) makes it difficult to compare **1c** directly to **1a** and **1b**. These results strongly suggest that heavy adamantyl groups hinder the vibrations and rotational motion of the C–N bond in Zeonex films that are associated with initial electron transfer and CT state relaxation. This effect is also reflected in the slightly shorter PF decay times for **1a** compared to **1b** in Zeonex (Table 1; the different CT character of **1c** means it cannot be directly compared to **1a** and **1b** in this regard). These same vibrational and twisting modes are also known to be involved in the rISC process in other D–A molecules.^15,16^

**Table 1:** Important Photophysical Parameters of **1a**, **1b**, and **1c**

compound	solvent/host	S_1_ (eV)^a^	T_1_ (eV)^b^	ΔE_ST_ (eV)^f^	PF lifetime (ns)^c^	DF lifetime (μs)^c^	*k*_f_ (×10^7^ s^–1^)^d^	*k*_ISC_ (×10^6^ s^–1^)^d^	*k*_rISC_ (×10^5^ s^–1^)^d^
**1a**	DCM	2.68		–0.13	21	3.6	2.6	16	4.5
	*o*-DCB	2.75		–0.06	23	2.0	3.72	4.0	5.4
	Zeonex	3.00	2.81	0.19	6.0	3.3 × 10^3^^e^			
	neat	3.11		0.30	7.0	1.6 and 62^e^			
**1b**	DCM	2.65		–0.13	24	5.0	2.0	21	4.3
	*o*-DCB	2.75		–0.03	23	3.4	3.3	5.2	2.9
	Zeonex	2.98	2.78	0.20	7.3	2.4 × 10^3^^e^			
	neat	3.10		0.32	11	19 and 83^e^			
**1c**	DCM	2.85		0.02	21	15	4.0	1.0	0.6
	*o*-DCB	2.95		0.12	17	5.8	4.9	1.2	1.4
	Zeonex	3.13	2.83	0.30	4.4	12 × 10^3^^e^			
	neat	3.05		0.22	7.3	3.5 and 197^e^			

a Determined from photoluminescence onset in different host/film environments.

b Determined from phosphorescence onset in Zeonex (80 K, 10 ms delay after pulsed excitation).

c Determined from amplitude weighted averages of double exponential fit components or individual component lifetimes for highly nonexponential neat film decays (fits in the SI, Figures S49–S57).

d Determined from kinetic fitting of PF and DF by the previously reported TADF model.^49^

e DF emission included a significant room-temperature phosphorescence (RTP) component, precluding extraction of rISC rates through kinetic fitting.

f S_1_ – T_1_, values < 0 indicate that the singlet state (CT character) lies below the phosphorescent and TADF-active triplet state (LE character) (see ref (44)).

While the early (7 ns) and late (27 ns) PF of **1a** almost coincide with the spectra of delayed emission (μs region), the emission of **1b** and **1c** experiences a red shift with time (Figure 8a–c). The decay kinetics (Figure 8d) and the monomolecular DF dependence on laser pulse energy (Figure S41) clearly indicate a thermally activated origin of the delayed emission. Interestingly, with further time delay (ms region), complex non-Gaussian photoluminescence is observed, corresponding to a mixture of late TADF emission and room-temperature phosphorescence (RTP). To demonstrate this, the late delayed emission is reconstructed from unambiguous PH spectra (collected at 80 K) along with early TADF emission (Figure S43).

While the room-temperature emission onsets at 400 nm for **1a–c** throughout the ms time region, the spectral shape varies with the nature and number of the substituents on the segments (Figure 8a–c). While only subtle shoulders at ca. 473 and 502 nm can be detected in the late ms-region PL of **1a**, the intensity of these peaks increases gradually with the introduction of the adamantyls on the spirofluorene donor part in **1b**, indicating a stronger contribution from PH emission (and weaker contribution from TADF). Finally, the addition of adamantyls to the pyrimidine acceptor results in the highest-intensity vibronically-shaped green RTP contribution. Interestingly, the transition from blue late DF to green PH occurs over time for all three materials. The observation of a stronger green PH component in **1b** compared to **1a** (Figure S43) cannot be due to differences in their electronic systems, which previous calculations and steady-state spectra show to be minimal. Instead, we propose that the reduced vibrational motion in these materials induced by the introduction of massive adamantyl substituents leads to hindered rISC and therefore more emission directly from triplet states compared to 1**a**. Furthermore, the introduction of massive bulky adamantyl fragments should significantly dampen large-amplitude vibrational motion (such as D–A dihedral rocking) in the solid Zeonex films. We propose that partially dampened (small-amplitude) D–A rocking and/or other localized vibrational modes (which may include acridine folding or acceptor rocking motions identified in calculations above) are instead responsible for the active rISC and TADF that is nonetheless observed in **1a** and **1b**.

### Solutions of Different Viscosities

The effect of adamantyl substitution was further investigated by comparing the time-resolved data of **1a–c** in degassed solutions of different viscosities. It is noteworthy that Feringa et al. demonstrated that the rotational speed of molecular rotors can be lowered when using high-viscosity solvents, and we expect similar damping of TADF-relevant molecular motions in viscous media.^40^ Solution measurements on **1a–c** would also allow for large-amplitude motions to more dominantly impact the DF and rISC kinetics, whereas these motions are restricted in solid state. Dichloromethane (DCM) and *o*-dichlorobenzene (*o*-DCB) were chosen due to only a slight difference in polarity (demonstrated by similar singlet onsets of **1a** and **1b**, Table 1) but a significant difference in viscosity.^41^ We note however that the solvent polarities, while similar, are not identical (dielectric constant of 9.99 for *o*-DCB and 8.93 for DCM).^42^ It is therefore only instructive to compare the isoelectronic, but differently dampened, materials **1a** and **1b** to each other in the same solvent, rather than to themselves across different solvents.

**Figure 9:**
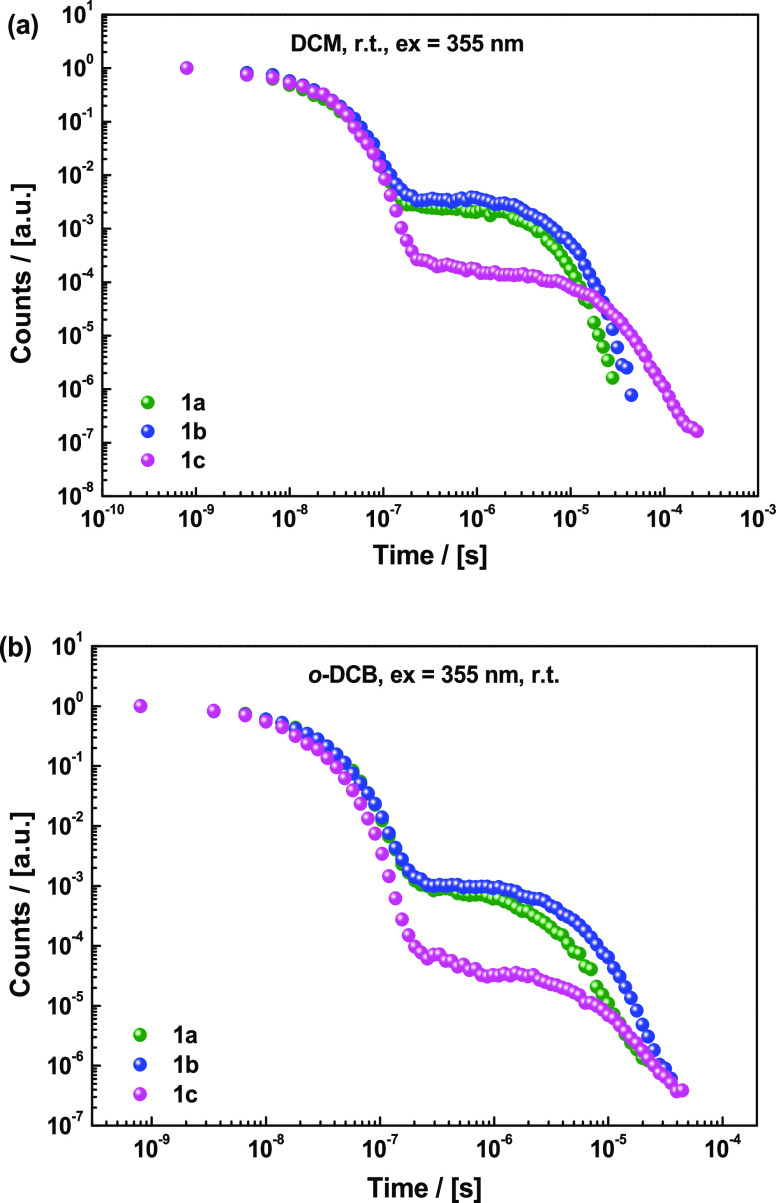
Decay curves of 5% (a) DCM and (b) *o*-DCB solutions of **1a**, **1b**, and **1c** recorded at room-temperature relaxation.

Due to the higher polarity of DCM and *o*-DCB compared to Zeonex, the CT state was stabilized in DCM and *o*-DCB leading to the significant narrowing of Δ*E*_ST_ (Table 1) and absence of PH emission in the DF (maps of normalized emission spectra, Figure S46). While all three compounds exhibit strong TADF in the abovementioned solvents (Figures S47 and S48), the decay dynamics show clear differences (Figure 9). In the less viscous DCM (0.45 cp), the DF intensity and decay rate increase in the order **1c** ≪ **1b** < **1a**. This indicates that the ISC and rISC rates are lower in **1c** (likely due to different Δ*E*_ST_ and CT character), but also that rISC is indeed hindered by the steric bulk on the donor in **1b**, which increases its mass and moment of inertia compared to **1a**. This interpretation is supported by fitted PF/DF exponential lifetimes included in Table 1 and kinetic parameters (*k*_f_, *k*_ISC_, and *k*_rISC_) determined by kinetic fitting of PF and DF simultaneously (Figures S49–S57).^43^ This result is not surprising from a classical standpoint but demonstrates in an unambiguous way that the vibronic coupling of singlet and triplet states is affected by the dynamic motion of the D–A bond.

The significant decrease in both ISC and rISC rates in **1c** may be due to both D and A being sterically encumbered, thereby significantly reducing D–A rotational motion and thus reducing the vibronic coupling that mediates rISC, although differences in CT character and donor strength present an additional unquantifiable contribution to TADF performance. The same trends are observed in the more viscous *o*-DCB (1.32 cp). The intensity of DF remains approximately the same in both **1a** and **1b**. While **1a** has a larger rISC rate than **1b** in this solvent as well, the gap between the two materials is significantly wider in the more viscous *o*-DCB. We suggest that this is because the adamantyl groups’ impact on vibrational motion in **1b** is amplified in the more viscous medium.

As was shown by Feringa et al.,^40^ attaching lateral groups to a molecule should increase its susceptibility to motional damping by viscous solvents due to entropic effects. Due to a combination of electronic and solvent viscosity arguments, the most heavily substituted **1c** also lags in terms of rISC and ISC in DCM and is the most sensitive to changes in viscosity. The DF lifetimes are consistently longer for the Zeonex films than for the solutions, although it is not possible to deconvolve these fitted lifetimes from the significant RTP emission observed in the polymer films.

### Neat Films

**Figure 10:**
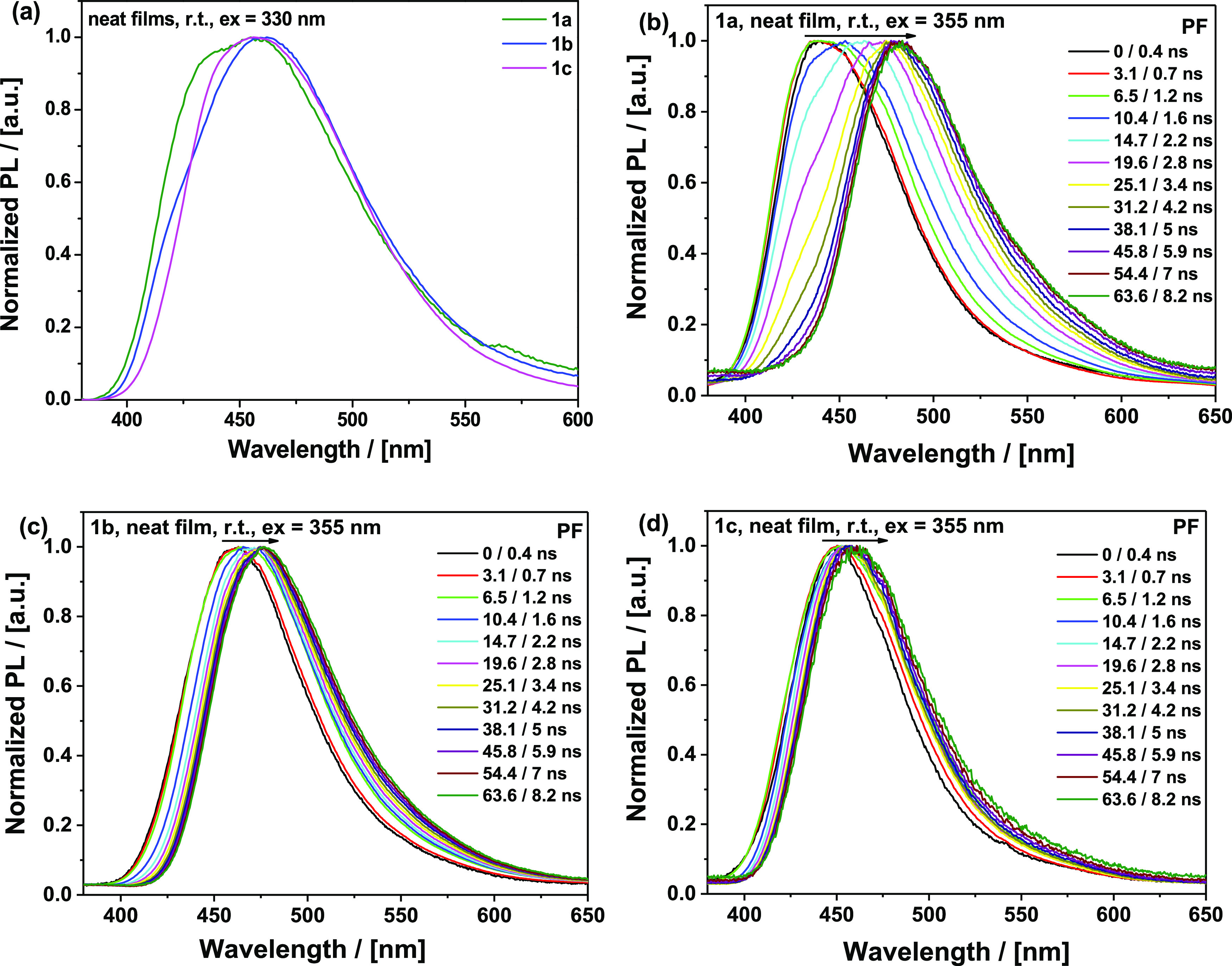
(a) Steady-state photoluminescence spectra and (b–d) time-resolved prompt photoluminescence spectra of neat films of **1a**, **1b**, and **1c** at specified delay/integration times (excitation 355 nm, Nd-YAG laser, room temperature).

Neat films of **1a–c** (drop-cast from toluene) were also investigated, as these are expected to give the most restrictive packing effects and damping of large-amplitude molecular motions. Interestingly, despite electronic system differences, the steady-state PL peak of the neat films (Figure 10a) is at the same position (ca. 458 nm) for all three materials. Both **1a** and **1b** feature non-Gaussian structures with a blue-shifted shoulder at 425–430 nm (appearing presumably due to the self-absorption in the neat films), hence resulting in relatively broad PL spectra with full width at half-minimum (FWHM) 90–98 nm. The Gaussian-shaped PL of **1c** is the narrowest within the series (FWHM 82 nm). The spectral evolution follows the opposite trend to what is seen in Zeonex. In that host, large changes in spectral shape were observed due to different contributions of LE donor emission. In neat films, material **1a** suffers the largest change in the spectrum over time, while **1c** remains relatively stable. Instead of the expected strong packing, we suggest that adamantyl fragments actually prevent tight packing in neat films that otherwise leads to π–π stacking, aggregation, and excimers of the pyrimidine section of the molecule, thus ensuring narrow and stable emission shape for **1c**. In contrast, **1a** with no such shielding from dimer species suffers significant spectral evolution, while **1b** presents an intermediate case.^44^

**Figure 11:**
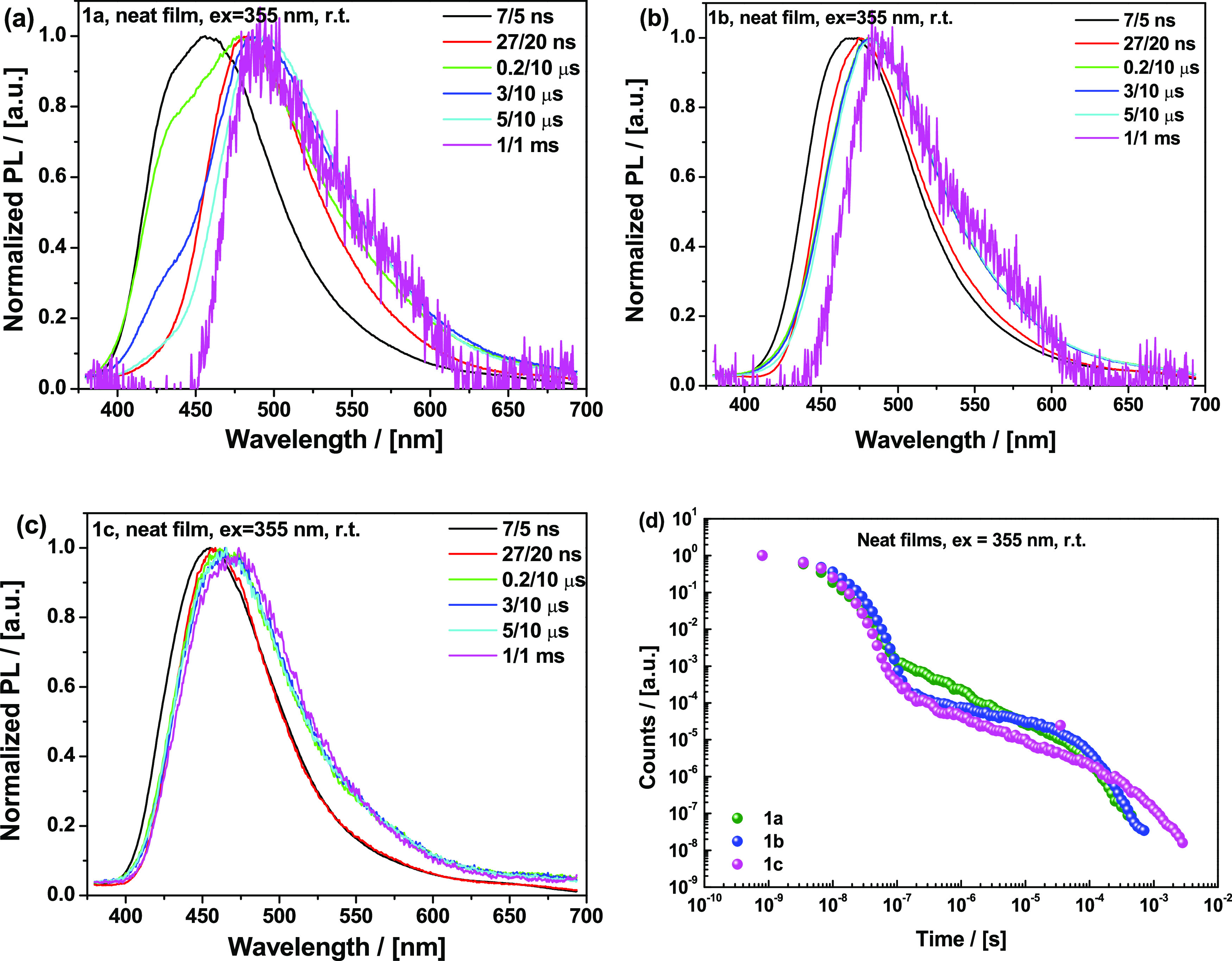
Time-resolved photoluminescence spectra of the neat films of (a) **1a**, (b) **1b**, and (c) **1c** recorded at room temperature at specified delay/integration times (excitation 355 nm, Nd-YAG laser). (d) Decay curves of the neat films of **1a**, **1b**, and **1c** recorded at room temperature.

Additionally, massive fragments are expected to increase inertia and slow the intersegmental motion, leading to restricted conformational relaxation that otherwise gives rise to the dispersion of dihedral angles and red-shifted spectra following excitation in the condensed phase. The time-resolved PF of the neat films (Figure 10b–d) increasingly red-shifts over time in the order **1c** < **1b** < **1a**, which is opposite to the trend observed in the Zeonex films (Figure 8a–c). Minimization of the PF red shift, which is generally attributed to the stabilization of the CT state,^39^ clearly points to the key role of the bulky adamantyl groups in preventing geometry relaxation. We speculate that different packing of the adamantyl substituents in neat films hinders rotational relaxation around the D–A bond, decreasing the rate of vibronically coupled rISC and increasing the DF lifetimes (Table 1) for all three materials compared to Zeonex or solution measurements, but most dramatically for the bulkiest material **1c**. Material **1a** shows the shortest lifetime and strongest thermally activated delayed emission in neat films (Figure 11), as it presents the smallest steric volume for other molecules to pack onto, thus allowing its acceptor region to still enjoy relatively unhindered rotation in the resulting void space. As in the Zeonex films though, contributions from both TADF and RTP emission make it difficult to make conclusive interpretations from a comparison of fitted DF lifetimes.

The above assumptions about neat films can be correlated with the X-ray packing patterns in the crystal structures (Figure S26). The packing mode of **1a** is quite tight with obvious parallel interactions between the pyrimidine-based acceptors. Such packing is favorable for the formation of dimer or excimer states, which may be responsible for the highly red-shifted (∼500 nm peak) millisecond emission seen in neat films of **1a** and **1b** (but not **1c**). In contrast, the molecules of **1b** are packed much more loosely in the crystal cell, without any evident interactions between the units. Adamantyl substituents tend to occupy much more space in the unit cell, acting as a “host” for the aromatic backbone and providing a packing environment of different rigidity. Indeed, the proximity of one adamantyl group to another will likely hinder the vibrations of the C–N bond in the emitter. Such cumulative effects of the self-hosting and vibrational hindrance can lead to the observed narrowing of the emission, as well as the different observed extent of spectral relaxation. In the case of **1c** bearing additional adamantyls on the acceptor fragment, the molecules are packed even more sparsely, leading to yet narrower PL at early times and a further reduction in spectral relaxation. The absence of excimer-like emission at long delay times in **1c** may also be due to the self-hosting effect of the adamantyl groups. We once again note the difficulty of comparing **1c** to the other materials, as its narrower early PL spectrum may also arise from its weaker CT character.

## Discussion

### Influence of the Vibrational Displacements on the (Reverse) Intersystem Crossing

Intersystem crossings are nonradiative transitions between different electronic states and play an important role in many phenomena, such as ultrafast phase transitions, catalysis, and photosynthesis.^45^ ISC between the electronic states of different multiplicity can occur if the states are coupled, i.e., the condition of spin–orbit coupling has to be satisfied. According to El-Sayed’s rule, this usually is the case for transitions between states of different characters in which the total angular momentum is conserved, which easily can be achieved in organometallic complexes. For purely organic compounds, this is a formally forbidden process; however, it becomes allowed and hence much faster for compounds exhibiting charge-transfer characteristics, in which CT states are coupled with LE states.

**Figure 12:**
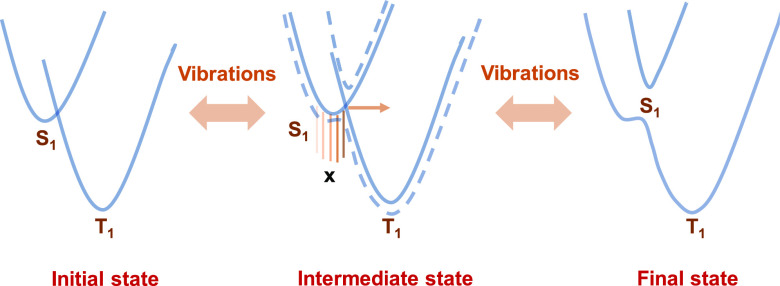
Vibrational displacement model. X—vibrational levels.

For organic molecules emitting via TADF, ISC is not only a one-way process, but thermally activated upconversion of triplet states within the reverse intersystem crossing step (rISC) also takes place. In a multistate coupling cascade mechanism, coupling of ^3^CT and ^3^LE states and subsequent spin-flip reversed intersystem crossing between ^3^LE and ^1^CT is believed to take place, making TADF a higher-order overall process. This picture becomes even more complex, as not only the respective energy gaps between triplet and singlet states are of importance, but spin-vibronic coupling between states is believed to be imperative. Disassembling the higher-order overall process into discrete IC/rIC and ISC/rISC steps, one can comprehend the intersystem crossing between the singlet and the triplet states in the TADF process using a series of dissipative quantum-mechanical two-potential-well systems (Figure 12).^46^

In the framework of this model, the dynamics cause the system to move between the potential wells and the dissipation ideally prevents a recurrence of the wave packet in the initial potential well. The mixing of states and population transfer between potential wells is primarily determined by the coupling constants and the Frank-Condon factors. For ISC and rISC to occur, it is crucial that energy is transported away from the “local” system of electronic and vibronic states. This can be achieved in sufficiently large systems (like the ones presented here), where the transition between the potential wells is thought to be mainly dominated by a single vibrational mode. In the absence of such a vibronic coupling to the nuclear degrees of freedom, the system would simply oscillate between the two electronic states.^47^ However, in the context of TADF materials, it is believed that especially small atom displacement modes are highly involved in the coupling process, making it difficult to be experimentally observed by transient optical absorption or time-resolved emission spectroscopy.^48^ In the case of TADF emitters, the potential wells correspond to the singlet (^1^CT) and triplet (^3^LE/^3^CT) states.^49,50^ When a large D–A system is excited, the vibrational modes between the states of different multiplicities are activated. As a result, energy transfer takes place from the vibronic modes of the triplet state to the vibronic modes of the singlet state (Figure 12), resulting in the repopulation of the well. Since the mode has changed significantly between the two wells, the interaction of the local system with the surroundings, and hence the dissipation, can be substantially different.

However, at this point, it remains unclear exactly which mode is relevant for promoting the interstate coupling process. The approach described within this paper probes the overall role of vibronic modes through damping, enabled by the rigidity of the molecular structure and the use of electronically innocent substituents. Substitution of the donor or acceptor segments with massive substituents on a remote molecular position, as presented herein, is expected to lead to an energy reduction of the dominant vibrational mode due to a change in the molecular segments’ inertia. This energy reduction would in turn change the efficiency of the repopulation of the wells by affecting the alignment of vibrational states and the respective nuclear coordinate. These factors will change the decay kinetics, even though the absolute energies, i.e., Δ*E*_ST_, of the involved states do not change. Lowering the coupling efficiency would lead to the reduced repopulation of the singlet state through rISC, increasing the probability of instead observing triplet deactivation by slow phosphorescence (which can be observed in the transient spectra) or nonradiative IC pathways.

From our results, it is clear that adding bulky adamantyls to the donor and acceptor severely hinders rotation/rocking about the D–A N–C bond, which produces a large reduction in the rISC rate in solution. Moreover, subtle differences are observed between optoelectronically identical **1a** and **1b**, which indicate that even structural changes in a distant part of a rigid molecule can alter intersegmental motions that affect the rISC process. In solid state, we confirm that hindering the large-amplitude motion of the acceptor about the D–A bridging bond greatly slows down rISC, but does not completely disable it. This points to two possible scenarios: (i) the large-amplitude motions are still at least partially active and mediate rISC at a much slower rate (commensurate with a smaller, damped amplitude); (ii) other low-amplitude vibrational modes also contribute to rISC and these remain mostly unaffected by packing in the solid state giving rise to the residual rISC. This would be particularly true in neat films. We also note that in simple spiro TADF materials rISC can be very efficient even though there are no large-amplitude modes available in the molecule comparable to D–A dihedral rocking^26^—and similarly so in exciplex^51−53^ and through-space TADF materials.^54,55^ While it is unclear what experimental methods may conclusively arbitrate these possibilities, our initial findings reaffirm the complexity of the vibronic coupling mechanism. If it indeed bears true that many vibrational modes can couple states to affect rISC (and other key decay channels), then the use of electronically innocent mechanical dampening, as outlined in this work, will provide a valuable tool for investigating the activity of such vibrational modes.

## Conclusions

Initially, the efficiency of TADF in light-emitting molecules was attributed primarily to the energy difference Δ*E*_ST_ between relevant singlet and triplet states; however, it has been observed that this simple two-state model is not sufficient to conclusively describe the underlying mechanism.^49,50^ Based on quantum dynamic calculations^11^ a vibronic coupling mechanism has been posited, but it is difficult to confirm this experimentally. In this paper, a model D–A system was reported which allows for the investigation of the effect of optically and electronically innocent groups that nonetheless impact the efficiency of vibronic coupling between singlet and triplet states relevant to rISC. The attachment of massive adamantyl groups on remote positions of a spiro-connected molecular system leads to a negligible electronic influence on the overall structure while affecting molecular dynamic modes within the molecule which in turn influence the emission properties.

Investigating molecules **1a** and **1b** dispersed in Zeonex polymer films and in solutions has demonstrated that adding adamantyl groups to the molecule (and thereby damping intramolecular motions) leads to differences in the delayed emission component without significantly changing Δ*E*_ST_ (Figure 8). In the case of the smaller molecule **1a**, efficient vibronic coupling between triplet and singlet states takes place. The attachment of heavy adamantyl groups in **1b** clearly changes the efficiency of the second-order vibronic coupling mechanism within the rISC step without impacting any of the relevant electronic parameters (inferred from spectra). The reduced vibronic coupling strength in **1b** therefore leads to longer emission times and an increased contribution of room-temperature phosphorescence to the overall emission profile in Zeonex. It is also observed that increasing the solvent viscosity has a greater impact on the rISC rate of the bulky molecule **1b** than on the lighter and smaller analog **1a**. This was attributed to entropic effects of the surrounding solvent molecules, which lead to a more severe damping effect in the case of molecule **1b**, than for **1a**. The more hindered intramolecular motion thereby leads to weakened vibronic coupling and lower efficiency of the rISC process. Nonetheless, rISC is still active in the restricted Zeonex environment, which could indicate vibronic coupling from other low-amplitude vibrational modes, or residual coupling from highly damped large-amplitude D–A dihedral rocking.

In the case of neat films of **1a–c**, different packing of adamantyl substituents is expected to hinder the rotational relaxation around the D–A bond, decreasing the rate of vibronically coupled rISC for all three materials compared to Zeonex or solution measurements—but most dramatically for the bulkiest material **1c**. Additional self-hosting effects lead to different degrees of dimerization (affecting spectral widths), although the least substituted **1a**, with the smallest molecular volume, retains the best rISC activity due to its ability to vibrate more within the void spaces of the film.

This work is, to the best of our knowledge, the first experimental report on vibronic coupling in isoelectronic (and therefore directly comparable) TADF materials—enabled by the effective electronic decoupling of D and A units at the spirofluorene group. For the design of future high-efficiency TADF materials, structural design should consider the possibility of fine-tuning vibrational coupling through damping or slimming down respective molecular segments, thereby maximizing the rISC rate and overall TADF properties. Future investigations of the TADF mechanism may reveal the role of other vibrational modes by mechanically dampening them in similar ways as explored here. These alternative or low-amplitude vibrational motions may be a driving factor in non-D–A TADF emitters, such as exciplexes^56,57^ and multiresonance boron–nitrogen materials, where large-amplitude modes for vibronic coupling like D–A dihedral angle rocking are not obvious.^58−60^

## Supporting Information Available

Synthetic details; ^1^H and ^13^C NMR spectra (including VT NMR data); additional crystallography and computational data; CV traces and data; additional absorption and emission spectra (PDF); and CIF files have been deposited with the Cambridge Structural Database, CCDC 2017592-2017595 (PDF)
