# Supplementary material for: Vibrational Damping Reveals Vibronic Coupling in Thermally Activated Delayed Fluorescence Materials
Source: Chem Mater. 2021 Apr 28;33(9):3066–80. doi: 10.1021/acs.chemmater.0c03783 (PMC8273894; doi:10.1021/acs.chemmater.0c03783)
Supplement: Supplementary file 1 — cm0c03783_si_001.pdf [file cm0c03783_si_001.pdf]

Supporting Information

Vibrational Damping Reveals Vibronic Coupling in Thermally Activated Delayed Fluorescence Materials

Matthias Hempe,[a] Nadzeya A. Kukhta,[a] Andrew Danos,[b] Mark A. Fox, [a] Andrei S. Batsanov, [a] Andrew P. Monkman,*[b] and Martin R. Bryce*[a]

[a] Chemistry Department, Durham University, South Road, Durham, DH1 3LE, UK

E-Mail: m.r.bryce@durham.ac.uk

[b] Physics Department, Durham University, South Road, Durham, DH1 3LE, UK

E-Mail: a.p.monkman@durham.ac.uk

1. Experimental procedures and characterization data 2

1.1. Overview 2

1.2. Synthesis of 2,7-dimethylfluorenone **3a** 4

1.3. Synthesis of 2,7-bis-(1-adamantyl)-9*H*-fluorene **5** 5

1.4. Synthesis of 2,7-bis-(1-adamantyl)-fluorenone **3b** 6

1.5. Synthesis of 2-Bromo-4,4’-dimethyldiphenylamine **7** 6

1.6. Synthesis of 2,2’-Tetramethyl-10*H*-spiro[acridine-9,9’-fluorene] **8a** 7

1.7. Synthesis of 2’,7’-Bis(adamantyl)-2,7-dimethyl-10*H*-spiro[acridine-9,9’-fluorene] **8b** 8

1.8. Synthesis of 2-(4-bromophenyl)-pyrimidine **10a** 9

1.9. Synthesis of 4,6-bis(1-adamantyl)-2-bromopyrimidine **11** 10

1.10. Synthesis of 4,6-bis(1-adamantyl)-2-(4-bromophenyl)pyrimidine **10b** 10

1.11. Syntheses of Donor-Acceptor compounds **1a-c** 11

2. NMR spectra 15

2.1. 2,7-dimethylfluorenone **3a** 15

2.2. 2,7-bis-(1-adamantyl)-9*H*-fluorene **5** 16

2.3. 2,7-bis-(1-adamantyl)-fluorenone **3b** 17

2.4. 2’,7’-Bis(adamantyl)-2,7-dimethyl-10*H*-spiro[acridine-9,9’-fluorene] **8b** 19

2.5. 2-(4-bromophenyl)-pyrimidine **10a** 20
2.6. 4,6-bis(1-adamantyl)-2-bromopyrimidine **11** 21
2.7. 4,6-bis(1-adamantyl)-2-(4-bromophenyl)pyrimidine **10b** 22
2.8. Donor-acceptor compound **1a** 23
2.9. Donor-acceptor compound **1b** 24
2.10. Donor-acceptor compound **1c** 25
3. VT-NMR 26
4. Single Crystal X-Ray Analysis 27
5. Calculations 31
6. Photophysical properties 41
7. Cyclic Voltammetry 59
8. References 60

1. Experimental procedures and characterization data

1.1. Overview

Attaching substituents at the fluorene site of the donor segment is based on the synthesis of respective fluorenone precursor molecules (**Scheme S1**). Here, methyl substituents can be attached in a two-fold Suzuki cross-coupling reaction using 2,7-diiodo-fluorenone **2** and trimethylboroxine. The attachment of bulky adamantyl groups at the 2,7-positions of this building block was instead achieved by the two-fold Friedel-Crafts alkylation reaction of fluorene **4** and 1-bromoadamantane using iron(III)chloride. The respective fluorene derivative **5** was subsequently oxidized at the benzylic 9-position, by adapting a procedure reported by Slawin et al.[1]

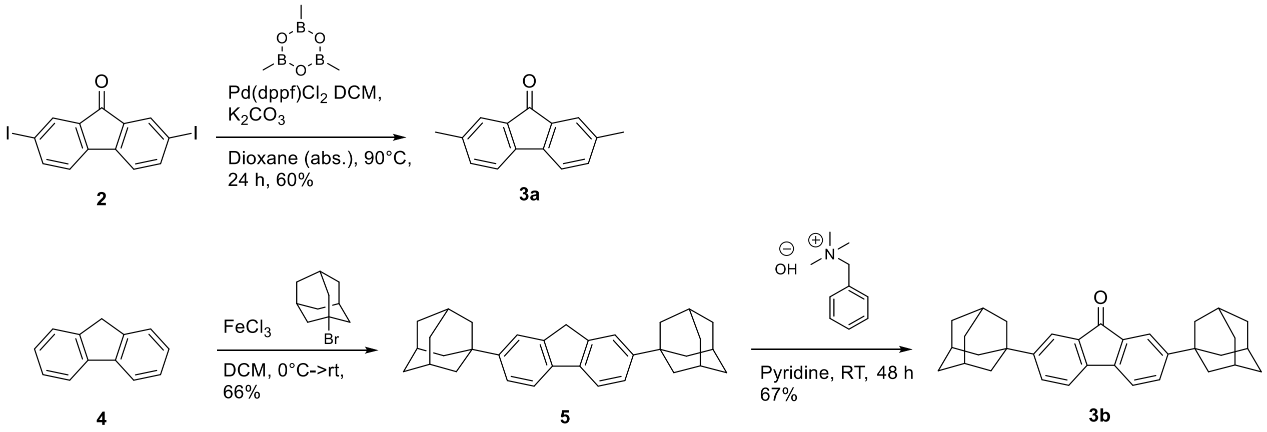

Scheme S1. Syntheses of 2,7-disubstituted fluorenone precursor molecules **3a-b**.

Using fluorenone compounds **3a-b**, the syntheses of acridine donor segments **8a-b** were achieved by adapting a reaction sequence previously reported for unsubstituted spiro-fluorene derivatives (**Scheme S2**).[2] Here, 2-bromo-4,4'-dimethyldiphenylamine **7** was obtained by Buchwald-Hartwig amination of the aniline derivative **6** and 4-iodotoluene. Compound **7** was subsequently used in a three-step reaction sequence. After halogen-metal exchange of **7**, the fluorenone compounds **3a-b** were used as electrophilic entities for the formation of the respective carbinol compounds, which were not isolated, but were subsequently cyclized to obtain acridine compounds **8a-b**.

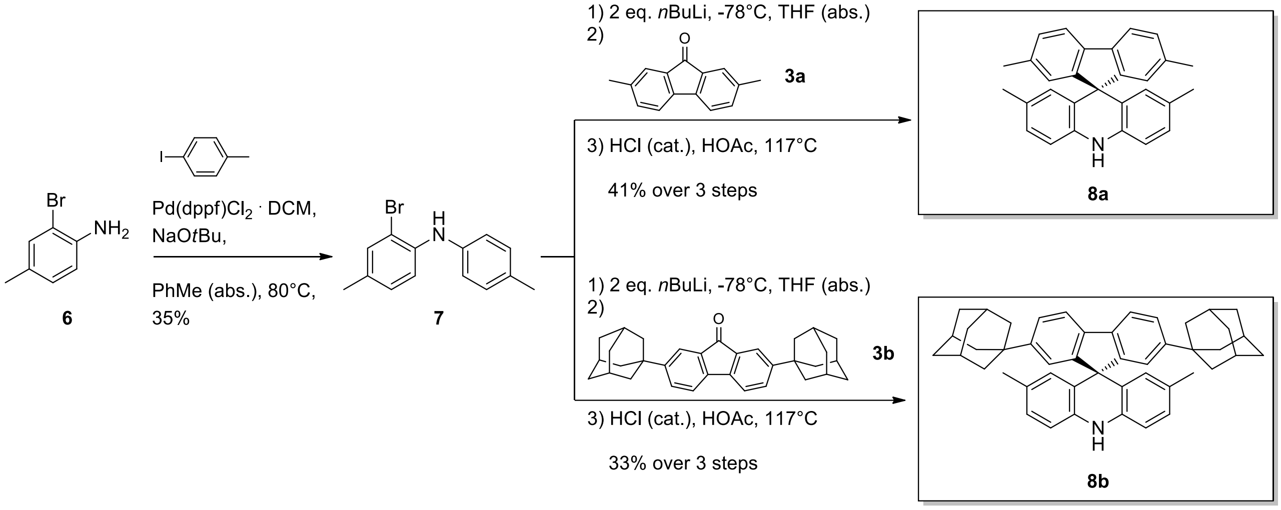

Scheme S2. Syntheses of acridine donor segments **8a-b**.

The synthesis of the unsubstituted pyrimidine-based acceptor unit **10a** was achieved by the Suzuki cross-coupling reaction of 2-bromopyrimidine **9** and 4-bromophenylboronic acid (**Scheme S3**). The adamantyl groups were attached to **9** in a two-fold Minisci reaction sequence using 1-adamantylcarboxylic acid. While the formation of the mono-adamantyl substituted pyrimidine intermediate can be readily observed,[3] the synthesis of di-substituted compound **11** required elevated reaction temperature and prolonged reaction time. Afterwards, the respective pyrimidine derivative **11** was reacted in a Suzuki cross-coupling reaction, giving the di-adamantyl-substituted acceptor segment **10b**.

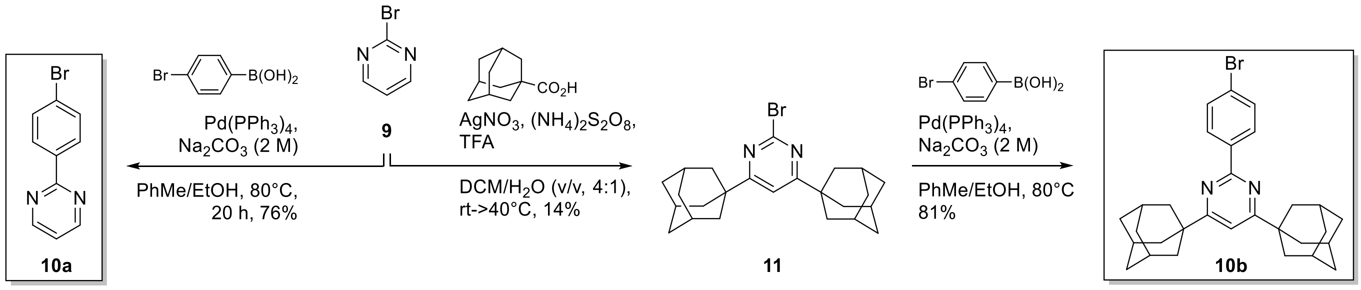

Scheme S3. Syntheses of pyrimidine-based acceptor segments **10a-b**.

Combining the acridine donor segments **8a-b** with the pyrimidine-based acceptor segments **10a-b** in final-step Buchwald-Hartwig amination reactions, gave the respective donor–acceptor compounds **1a-c** in high yields (**Scheme S4**).

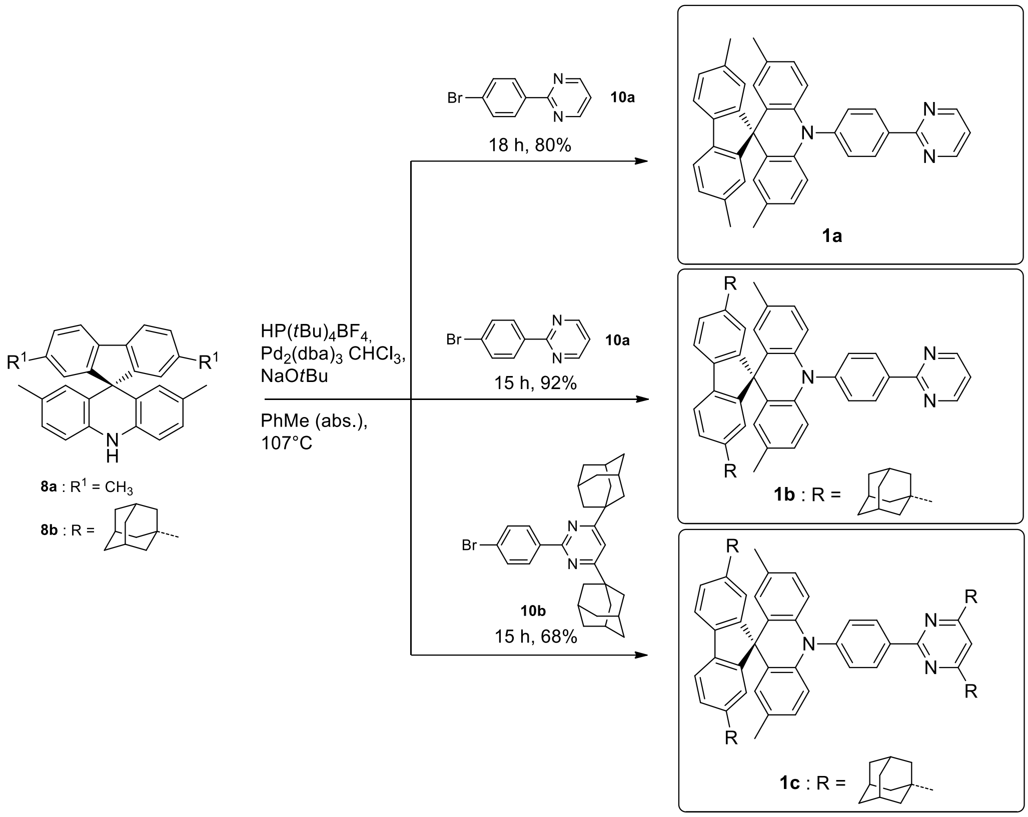

Scheme S4. Syntheses of donor-acceptor compounds **1a-c** by Buchwald-Hartwig amination reaction of acridine segments **8a-b** and pyrimidine-based acceptor units **10a-b**.

1.2. Synthesis of 2,7-dimethylfluorenone 3a

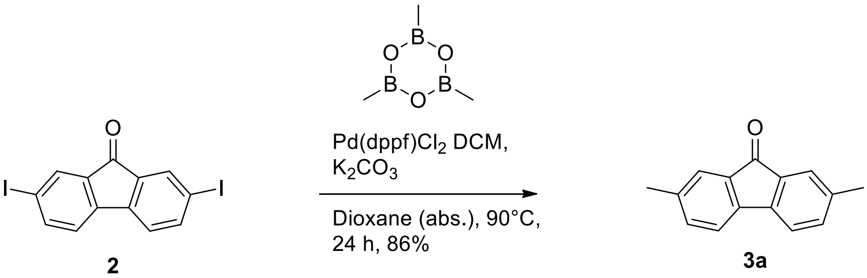

Under an argon atmosphere, 4.50 g (13.31 mmol) 2,7-dibromofluorenone and 7.36 g (4.00 eq., 53.26 mmol) K_2CO_3 were mixed with 200 mL 1,4-dioxane. The mixture was purged with argon for 20 minutes, after which 5.01 g (3.00 eq, 39.94 mmol) trimethylboroxine were added. After adding 652 mg (0.06 eq., 0.80 mmol) $Pd(dppf)Cl_2$ · DCM, the mixture was heated to 90 °C for 20 h. The mixture was cooled to room temperature, was diluted with 50 mL DCM and was filtered through a plug

of silica. The solvent was removed under reduced pressure and the residue was purified by column chromatography on silica (Hex/DCM, 3:1→1:1 v/v), resulting in 2.38 g (86%, 11.43 mmol) of the product **3a** as a yellow solid.

^{1}H-NMR ($CDCl_3$, 600 MHz, 298 K) δ (ppm) = 2.34 (s, 6H), 7.23 (d, 2H, J = 7.52 Hz), 7.32 (d, 2H, J = 7.52 Hz), 7.418 (s, 2H).

^{13}C-NMR ($CDCl_3$, 150 MHz, 298 K) δ (ppm) = 21.29, 119.78, 124.90, 134.52, 134.99, 138.67, 142.00, 194.42.

HRMS-ASAP-TOF$^+$ *m/z* calculated for $C_{15}H_{13}O$ $[M]^+$ 209.0966, found $[M]^+$ 209.0965.

M.p. 157 °C

1.3. Synthesis of 2,7-bis-(1-adamantyl)-9*H*-fluorene 5

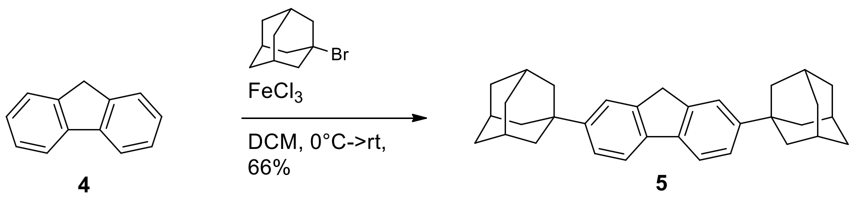

Under an argon atmosphere, 2.50 g (15.04 mmol) fluorene were mixed with 97.5 mg (0.04 eq., 0.60 mmol) $FeCl_3$ and 10 mL DCM (dry). The mixture was cooled to 0 °C and a solution of 9.77 g (3.02 eq., 45.42 mmol) of 1-bromoadamantane in 10 mL DCM (dry) was added dropwise. The brownish mixture was stirred at 0 °C for 4 h and subsequently was warmed to room temperature. After further 4 h, the reaction mixture was filtered through a plug of silica using DCM. The solvent was removed under reduced pressure, yielding an off-white solid. The residue was mixed with 180 mL EtOH and was heated to 78 °C for 3 h. Afterwards, the solid was filtered from the hot mixture and the procedure was repeated two times, yielding 4.34 g (66%, 9.97 mmol) of the product **5** as a white solid.

^{1}H-NMR (CD_2Cl_2, 400 MHz, 298 K) δ (ppm) = 1.82 (m, 12H), 1.99 (s, 12H), 2.12 (s, 6H), 3.86 (s, 2H), 7.37 (d, 2H, J = 8.05 Hz), 7.56 (s, 2H), 7.68 (d, 2H, J = 8.05 Hz).

^{13}C-NMR (CD_2Cl_2, 150 MHz, 298 K) δ (ppm) = 29.19, 36.29, 36.78, 37.02, 43.40, 118.97, 121.48, 123.28, 139.03, 143.36, 149.92.

HRMS-ASAP-TOF$^+$ *m/z* calculated for $C_{33}H_{38}$ $[M]^+$ 434.2974, found $[M]^+$ 434.2931.

M.p. 309 °C

1.4. Synthesis of 2,7-bis-(1-adamantyl)-fluorenone 3b

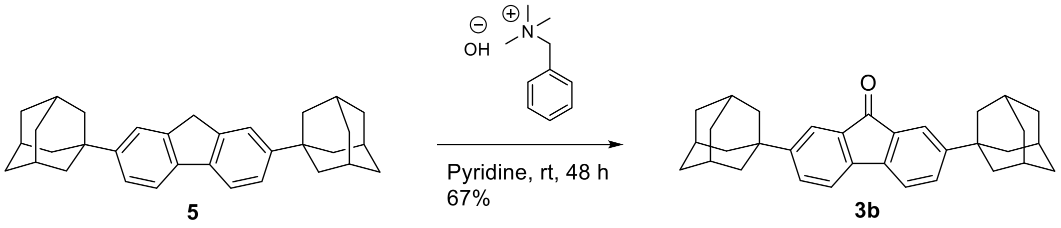

In an adaption of a literature procedure,[4] 2,7-bis-(1-adamantyl)-9*H*-fluorene **5** (2.50 g, 5.75 mmol) was mixed with 25 mL pyridine. Under vigorous stirring, 3.2 mL (1.35 g, 1.4 eq., 8.05 mmol) benzyltrimethylammonium hydroxide were added dropwise and the resulting mixture was stirred at room temperature for 72 h. Afterwards, the reaction mixture was hydrolyzed by the addition of 20 mL water, neutralized using acetic acid, and subsequently was extracted using DCM (3 x 20 mL). The organic layers were washed with copper sulfate solution (aq.) and water and were dried using Na_2SO_4. The solvent was removed under reduced pressure and the residue was purified by column chromatography on silica (Hex/DCM, 9:1 → 3:1 v/v). The product **3b** was obtained as a yellow solid (67%, 1.74 g, 3.88 mmol).

^{1}H-NMR (CD_2Cl_2, 400 MHz, 298 K) δ (ppm) = 1.80 (m, 12H), 1.94 (m, 12H), 2.11 (m, 6H), 7.45 (d, 2H, J = 7.80, 0.51 Hz), 7.48 (s, 2H, J = 7.80, 1.84 Hz), 7.65 (d, 2H, J = 1.84, 0.51 Hz).

^{13}C-NMR (CD_2Cl_2, 150 MHz, 298 K) δ (ppm) = 28.98, 36.49, 36.56, 42.91, 119.80, 120.85, 131.09, 134.60, 141.83, 152.61, 194.37.

HRMS-ASAP-TOF$^+$ *m/z* calculated for $C_{33}H_{36}O$ $[M]^+$ 448.2766, found $[M]^+$ 448.2759.

M.p. 338 °C

1.5. Synthesis of 2-Bromo-4,4'-dimethyldiphenylamine 7

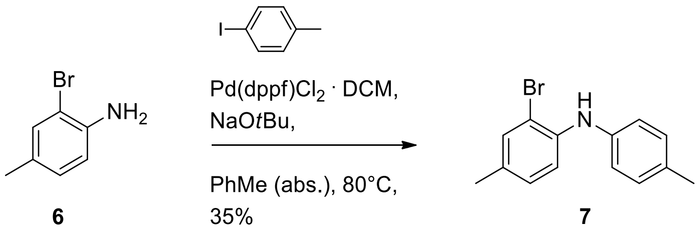

Following a literature procedure[5] and under an argon atmosphere, 17.50 g (94.06 mmol) 2-bromo-4-methylaniline and 21.43 g (1.05 eq., 98.29 mmol) 4-iodotoluene were mixed with 280 mL toluene (abs.). The mixture was purged with argon for 30 minutes, after which 1.15 g (0.015 eq., 1.41 mmol) $Pd(dppf)Cl_2$ · DCM were added. The mixture was heated to 80 °C, 18.98 g (2.1 eq., 197.52 mmol) NaO*t*Bu were added and the resulting brown suspension was stirred at 80 °C for 80 h. The mixture was

cooled to room temperature, was diluted using 150 mL ethyl acetate and was washed with 300 mL water. The organic phase was concentrated under reduced pressure and the residue was dissolved in DCM. The solution was dried using $MgSO_4$ and was then filtered through a plug of silica. The solvent was removed *in vacuo* and the remaining oil was purified by column chromatography on silica (*n*-Hex/DCM, 9:1 v/v). The combined, product-containing fractions were further purified by column chromatography using a plug of silica and *n*Hex as eluent. The pure product was obtained as an orange coloured oil (35%, 8.99 g, 32.58 mmol) which tends to crystalize after a while.

The NMR data match those reported in the literature.[5]

^{1}H-NMR (CD_2Cl_2, 400 MHz, 294 K) δ (ppm) = 2.29 (s, 3H), 2.34 (s, 3H), 5.93 (s, 1H), 6.99-7.07 (m, 3H), 7.11 (d, 1H, *J* = 8.33 Hz), 7.15 (d, 2H).

1.6. Synthesis of 2,2'-Tetramethyl-10*H*-spiro[acridine-9,9'-fluorene] 8a

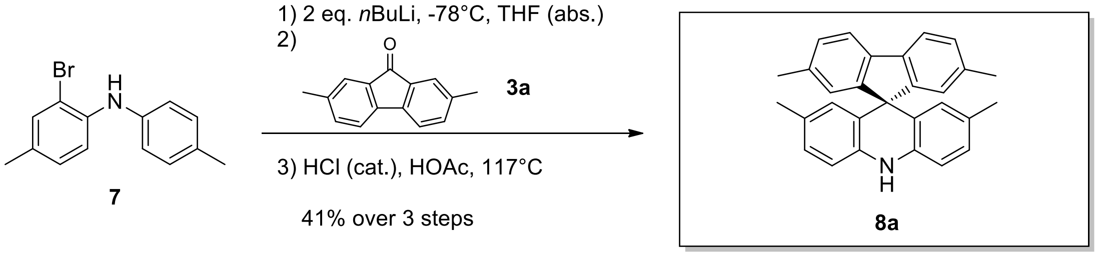

Under an argon atmosphere, 1.70 g (6.16 mmol) 2-bromo-4,4'-dimethyldiphenylamine **7** were dissolved in 20 mL THF (dry) and cooled to -78 °C. In a separate flask, 6.03 mL (2.5 M, 2.45 eq., 15.08 mmol) of a solution of *n*-butyl lithium in *n*-hexane were cooled to -78 °C and the precooled solution of the aryl bromide was added dropwise. The yellow solution was stirred for 2 h at -78 °C. A precooled mixture of 1.28 g (1.00 eq., 6.16 mmol) 2,7-dimethylfluorenone **3a** in 40 mL Et_2O (dry) was carefully added to the solution of the metalated species and the mixture was stirred for 4 h at -78 °C. The reaction was quenched by the addition of 20 mL saturated NaCl solution (aq.). The phases were separated, and the organic layer was concentrated under reduced pressure, resulting in a red glass. The residue was dissolved in 120 mL glacial acid and was heated to 90 °C under an argon atmosphere. At 90 °C, 10 drops of conc. HCl were added and the mixture was heated to 117 °C for 20 h. The mixture was cooled to room temperature and was concentrated *in vacuo*. The residue was dissolved in 200 mL DCM and subsequently was washed sequentially with sat. $NaHCO_3$ solution (aq., 3 x 150 mL), water (3 x 100 mL), and 200 mL sat. NaCl solution (aq.). The organic layer was dried using $MgSO_4$, and the solvent was removed under reduced pressure. The residue was purified by column chromatography on silica (*n*Hex/DCM, 3:1 v/v), resulting in 970 mg (41%, 2.50 mmol) of **8a** as an off-white solid.

^{1}H-NMR (Acetone-d$_6$, 700 MHz, 298 K) δ (ppm) = 1.91 (s, 6H), 2.24 (s, 6H), 6.05 (s, 2H), 6.83 (d, 2H, J = 8.16 Hz), 6.86 (d, 2H, J = 8.16 Hz), 7.02 (s, 2H), 7.15 (d, 2H, J = 7.80 Hz), 7.70 (d, 2H, J = 7.80 Hz), 8.13 (s, 1H).

^{13}C-NMR (Acetone-d$_6$, 176 MHz, 298 K) δ (ppm) = 19.76, 20.72, 56.57, 114.06, 119.28, 123.67, 126.05, 127.62, 128.07, 128.13, 128.21, 136.69, 137.29, 137.48, 156.98.

HRMS-ASAP-TOF$^+$ *m/z* calculated for $C_{29}H_{25}N$ [M]$^+$ 387.1987, found [M]$^+$ 387.1985.

M.p. decomp. 260 °C

1.7. Synthesis of 2’,7’-Bis(adamantyl)-2,7-dimethyl-10*H*-spiro[acridine-9,9’-fluorene] 8b

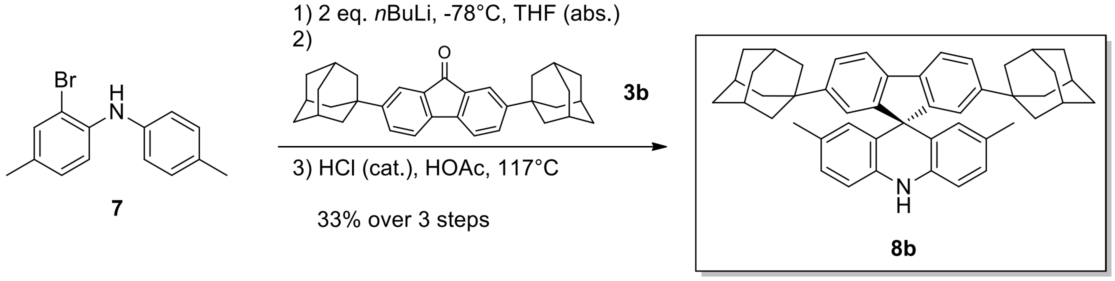

Under an argon atmosphere, 1.75 g (6.34 mmol) 2-bromo-4,4’-dimethyldiphenylamine **7** were dissolved in 20 mL THF (dry) and cooled to -78 °C. In a separate flask, 6.00 mL (2.5 M, 2.37 eq., 15.01 mmol) of a solution of *n*-butyl lithium in *n*-hexane were cooled to -78°C and the precooled solution of the aryl bromide was added dropwise. The yellow solution was stirred for 2 h at -78 °C. The solution of the metalated species was carefully added to a precooled mixture of 2.70 g (0.95 eq., 6.02 mmol) 2,7-bis(adamantyl)-fluorenone **3b** in 300 mL THF (dry). The resulting mixture was stirred for 17 h at -78 °C. Afterwards, the reaction was quenched by the addition of 50 mL saturated NaCl solution (aq.). The phases were separated, and the organic layer was concentrated under reduced pressure. The residue was dissolved in 160 mL glacial acid and was heated to 90 °C under an argon atmosphere. At 90 °C, 10 drops of conc. HCl were added and the mixture was heated to 117 °C for 7 h. The mixture was cooled to room temperature and was concentrated *in vacuo*. The residue was dissolved in 300 mL DCM and subsequently was washed sequentially with sat. $NaHCO_3$ solution (aq., 3 x 150 mL), water (3 x 100 mL), and 200 mL sat. NaCl solution (aq.). The organic layer was dried using $MgSO_4$, and the solvent was removed under reduced pressure. The residue was purified by column chromatography on silica (*n*Hex/DCM, 5:1 -> 3:1 v/v), resulting in 1.26 g (33%, 2.01 mmol) of **8b** as an off-white solid.

^{1}H-NMR (DMSO-d$_6$, 600 MHz, 298 K) δ (ppm) = 1.64 (s, 12H), 1.71 (s, 12H), 1.87 (s, 6H), 1.95 (s, 6H), 5.88 (s, 2H), 6.82 (d, 2H, J = 8.13 Hz), 6.86 (d, 2H, J = 8.13 Hz), 7.15 (s, 2H, J = 1.46 Hz), 7.32 (d, 2H, J = 8.07, 1.76 Hz), 7.71 (d, 2H, J = 8.07 Hz), 8.93 (s, 1H).

^{13}**C-NMR** (DMSO-d_6, 150 MHz, 298 K) δ (ppm) = 20.80, 28.66, 36.31, 36.49, 43.15, 57.18, 114.42, 119.87, 121.54, 124.33, 124.63, 126.90, 127.96, 128.51, 136.62, 138.21, 151.16, 155.74.

HRMS-ASAP-TOF$^+$ *m/z* calculated for $C_{47}H_{49}N$ $[M]^+$ 627.3865, found $[M]^+$ 627.3879.

M.p. decomp. 280 °C

1.8. Synthesis of 2-(4-bromophenyl)-pyrimidine 10a

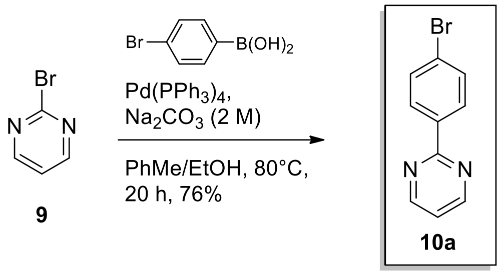

In an adaption of a literature procedure,[6] 801 mg (1.5 eq., 5.04 mmol) 2-bromopyrimidine were dissolved in 6 mL toluene and a solution of 1.51 g (3.25 eq., 10.92 mmol) sodium carbonate in 6 mL water was added. The mixture was purged with argon for 20 minutes, after which 116.5 mg (0.03 eq., 0.10 mmol) $Pd(PPh_3)_4$ were added. The mixture was heated to 50 °C and a degassed solution of 675 mg (1.00 eq., 3.36 mmol) 4-bromophenylboronic acid in 6 mL ethanol was added dropwise over the period of 2 h. The mixture was stirred for further 18 h at 50 °C and subsequently was diluted using 20 mL dichloromethane. The mixture was filtered through plugs of celite and silica, and afterwards was concentrated under reduced pressure. The residue was purified by column chromatography on silica (DCM/Hex, 1:1->3:1 v/v), resulting in 601 mg (76%, 2.56 mmol) of **10a** as an off-white solid.

^{1}**H-NMR** (CD_2Cl_2, 700 MHz, 298 K) δ (ppm) = 7.230 (t, 1H, J = 4.81 Hz), 7.630 (d, 2H, J = 8.63 Hz), 8.350 (d, 2H, J = 8.63 Hz), 8.790 (d, 2H, J = 4.81 Hz).

^{13}**C-NMR** (CD_2Cl_2, 175 MHz, 298 K) δ (ppm) = 119.43, 125.25, 129.65, 131.65, 136.73, 157.27, 163.57.

HRMS-ASAP-TOF$^+$ *m/z* calculated for $C_{10}H_8BrN_2$ $[M+H]^+$ 234.9871, found $[M+H]^+$ 234.9866.

M.p. 136-137 °C

1.9. Synthesis of 4,6-bis(1-adamantyl)-2-bromopyrimidine 11

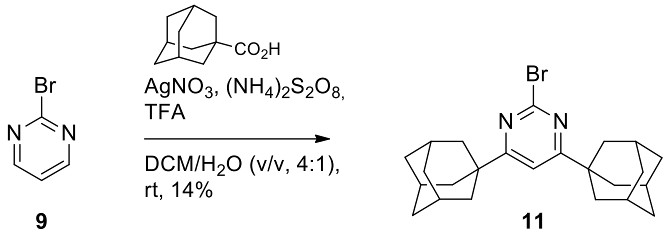

In modification of a literature procedure,[3] 1.10 g (6.92 mmol) 2-bromopyrimidine **9** and 7.48 g (6 eq., 41.51 mmol) 1-adamantylcarboxylic acid were dissolved in 40 mL DCM and 2.12 mL (4 eq., 27.67 mmol) trifluoroacetic acid diluted in 10 mL water were added. To the biphasic mixture, 2.11 g (1.8 eq., 12.45 mmol) $AgNO_3$ were added, and a solution of 9.47 g (6 eq., 41.51 mmol) $(NH_4)_2S_2O_8$ in 30 mL water was added dropwise over the period of 30 min. The mixture was stirred at room temperature for 24 h and was subsequently heated to 40 °C for further 3 h. The reaction mixture was poured onto crushed ice and the resulting mixture was made alkaline using a solution of NH_4OH (40%). After extraction with ethyl acetate (3 x 80 mL), the organic phase was dried using $MgSO_4$ and was concentrated under reduced pressure. The residue was purified by column chromatography (Hex/DCM, 3:1->1:1 v/v), yielding 405 mg (14%, 0.95 mmol) of **11** as a colorless solid. The obtained material was sufficiently pure to be used in the next step of the reaction sequence.

^{1}H-NMR (CD_2Cl_2, 700 MHz, 298 K) δ (ppm) = 1.79 (m, 12H), 1.94 (s, 12H), 2.10 (s, 6H), 7.14 (s, 1H).

^{13}C-NMR (CD_2Cl_2, 175 MHz, 298 K) δ (ppm) = 28.43, 36.43, 39.52, 40.90, 110.56, 152.67, 180.94.

HRMS-ASAP-TOF$^+$ *m/z* calculated for $C_{24}H_{32}BrN_2$ $[M+H]^+$ 427.1749, found $[M+H]^+$ 427.1748.

M.p. 200-203 °C

1.10. Synthesis of 4,6-bis(1-adamantyl)-2-(4-bromophenyl)pyrimidine 10b

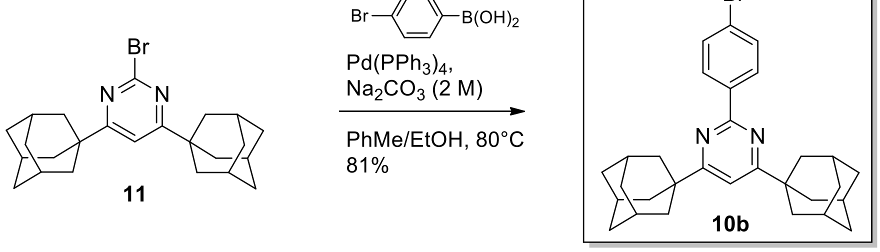

In adaption of a literature procedure,[6] 306 mg (1.2 eq., 0.72 mmol) 4,6-bis(1-adamantyl)-2-bromopyrimidine **11** were dissolved in 6 mL toluene and a solution of 268 mg (3.25 eq., 1.94 mmol)

sodium carbonate in 6 mL water were added. The mixture was purged with argon for 20 minutes, after which 21 mg (0.03 eq., 0.02 mmol) $Pd(PPh_3)_4$ were added and the mixture was heated to 50 °C. A degassed solution of 120 mg (1.00 eq., 0.60 mmol) 4-bromophenylboronic acid in 6 ml ethanol were added dropwise over the period of 1.5 h. The mixture was stirred at 50 °C for 18 h, was cooled to room temperature and was diluted using 20 mL dichloromethane. The mixture was filtered through plugs of celite and silica, and was concentrated under reduced pressure. The residue was purified by column chromatography on silica (Hex/DCM, 3:1 v/v), yielding 244 mg (81%, 0.48 mmol) of **10b** as a colorless solid.

^{1}H-NMR (CD_2Cl_2, 700 MHz, 298 K) δ (ppm) = 1.83 (m, 12H), 2.05 (m, 12H), 2.13 (m, 6H), 7.11 (s, 1H), 7.60 (d, 2H, J = 8.43 Hz), 8.45 (d, 2H, J = 8.43 Hz).

^{13}C-NMR (CD_2Cl_2, 175 MHz, 298 K) δ (ppm) = 28.77, 36.69, 39.37, 41.26, 109.29, 124.37, 129.78, 131.28, 138.12, 161.59, 177.31.

HRMS-ASAP-TOF$^+$ *m/z* calculated for $C_{30}H_{36}BrN_2$ $[M+H]^+$ 503.2062, found $[M+H]^+$ 503.2073.

M.p. 249-251 °C

1.11. Syntheses of Donor-Acceptor compounds 1a-c

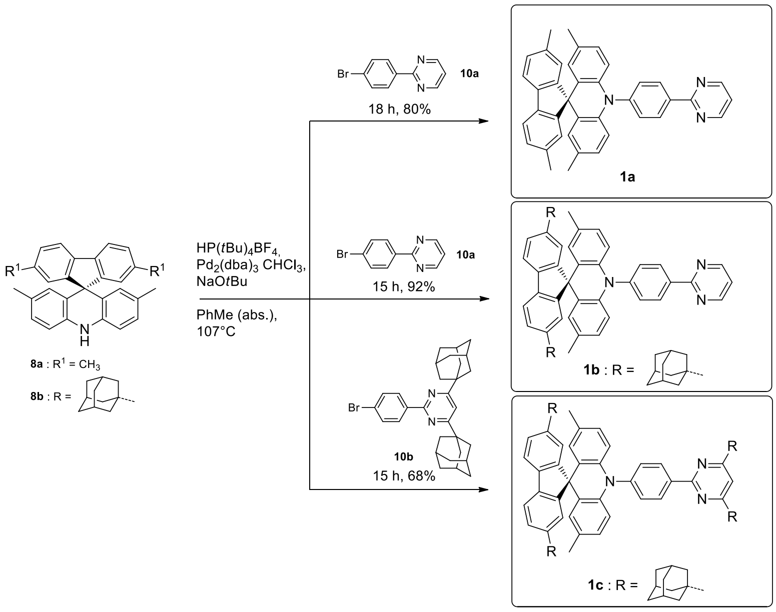

General procedure

Under an argon atmosphere, 1 eq. of the bromide **10a-b** and 1.05 eq. of the acridine component **8a-b** were dissolved in toluene (0.1 M, abs.). To this solution, 0.1 eq. $HP(tBu)_4BF_3$ were added and the mixture was purged with argon for 30 min. Afterwards, 0.04 eq. $Pd_2(dba)_3 \cdot CHCl_3$ were added and the

mixture was heated to 107 °C. At this temperature, 2.5 eq. NaO*t*Bu were added and the mixture was stirred until completion of the reaction. The mixture was cooled to room temperature and was filtered through plugs of celite and silica using DCM as eluent. The solvent was removed under reduced pressure and the residue was purified by column chromatography on silica (DCM/Hex, 1:1 → 3:1 v/v). After removal of the solvent *in vacuo*, the remaining solid was thoroughly washed using cold *n*-pentane and was dried under high vacuum. Crystals for X-ray analysis were grown by slow evaporation of solvent CD_2Cl_2 in an NMR tube.

1.11.1. Synthesis of 1a

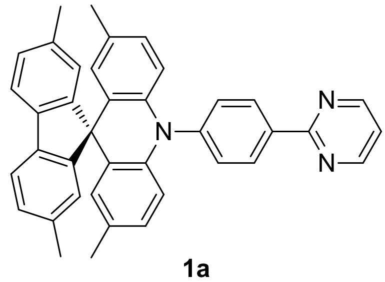

1a

Following the general procedure, 84 mg (0.36 mmol) 2-(4-bromophenyl)-pyrimidine **10a** and 145 mg (1.05 eq., 0.38 mmol) acridine **8a** gave 155 mg (80%, 0.29 mmol) of **1a** as a colorless solid.

^{1}H-NMR (CD_2Cl_2, 700 MHz, 298 K) δ (ppm) = 1.96 (s, 6H), 2.35 (s, 6H), 6.17 (s, 2H), 6.35 (d, 2H, *J* = 8.40 Hz), 6.75 (d, 2H *J* = 8.40 Hz), 7.20 (s, 2H), 7.21 (d, 2H, *J* = 7.68 Hz), 7.29 (t, 1H, *J* = 4.78 Hz), 7.62 (d, 2H, *J* = 8.16 Hz), 7.69 (d, 2H, *J* = 7.68 Hz), 8.80 (d, 2H, *J* = 8.16 Hz), 8.89 (d, 2H, *J* = 4.78 Hz).

^{13}C-NMR (CD_2Cl_2, 175 MHz, 298 K) δ (ppm) = 20.01, 21.40, 56.43, 114.57, 119.34, 119.43, 124.81, 125.97, 127.81, 127.84, 128.34, 129.41, 130.72, 131.36, 136.71, 137.67, 137.82, 139.06, 143.82, 156.96, 157.39, 163.88.

HRMS-ASAP-TOF^{+} *m/z* calculated for $C_{39}H_{32}N_3$ $[M+H]^+$ 542.2596, found $[M+H]^+$ 542.2584.

M.p. 311 °C

1.11.2. Synthesis of 1b

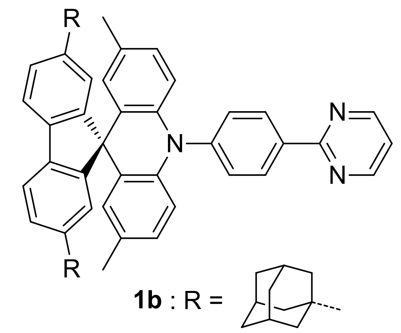

Following the general procedure, 80 mg (0.34 mmol) 2-(4-bromophenyl)-pyrimidine **10a** and 224 mg (1.05 eq., 0.36 mmol) acridine **8b** gave 245 mg (92%, 0.31 mmol) of **1b** as a colorless solid.

^{1}H-NMR (CD_2Cl_2, 600 MHz, 298 K) δ (ppm) = 1.77 (m, 12H), 1.91 (s, 12H), 1.98 (s, 6H), 2.08 (s, 6H), 6.24 (s, 2H), 6.42 (d, 2H, J = 8.41 Hz), 6.77 (d, 2H, J = 8.41 Hz), 7.29 (t, 1H, J = 4.79 Hz), 7.42 (d, 2H, J = 8.03, 1.05 Hz), 7.51 (s, 2H), 7.66 (d, 2H), 7.72 (d, 2H, J = 8.03 Hz), 8.81 (d, 2H, J = 8.20 Hz), 8.89 (d, 2H, J = 4.80 Hz).

^{13}C-NMR (CD_2Cl_2, 150 MHz, 298 K) δ (ppm) = 20.09, 29.11, 36.47, 36.69, 43.29, 57.31, 114.38, 119.18, 119.44, 121.75, 124.32, 126.22, 126.96, 127.67, 129.62, 130.67, 131.26, 136.82, 137.59, 139.58, 143.79, 151.54, 155.12, 157.39, 163.86.

HRMS-ASAP-TOF$^+$ *m/z* calculated for $C_{34}H_{23}N_4$ $[M+H]^+$ 487.1923, found $[M+H]^+$ 487.1916.

M.p. 309-313 °C

1.11.3. Synthesis of 1c

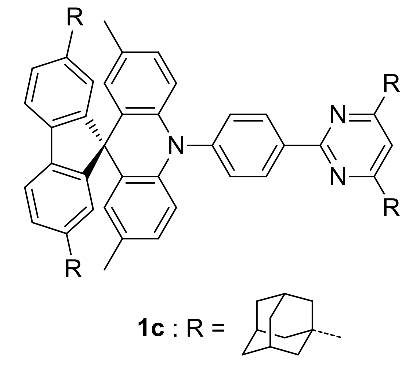

Following the general procedure, 120 mg (0.24 mmol) 4,6-bis(1-adamantyl)-2-(4-bromophenyl)-pyrimidine **10b** and 162 mg (1.08 eq., 0.26 mmol) acridine **8b** gave 170 mg (68%, 0.16 mmol) of **1c** as a colorless solid.

^{1}H-NMR (CD_2Cl_2, 700 MHz, 298 K) δ (ppm) = 1.78 (m, 12H), 1.87 (m, 12H), 1.91 (m, 12H), 1.98 (s, 6H), 2.13 (m, 24H), 6.22 (s, 2H, *J* = 1.95 Hz), 6.41 (d, 2H, *J* = 8.50 Hz), 6.76 (d, 2H, *J* = 8.50 Hz), 7.18 (s, 1H), 7.42 (d, 2H, *J* = 8.05, 1.79 Hz), 7.50 (s, 2H, *J* = 1.85 Hz), 7.61 (d, 2H, *J* = 8.40 Hz), 7.72 (d, 2H, *J* = 8.05 Hz), 8.89 (d, 2H, *J* = 8.40 Hz).

^{13}C-NMR (CD_2Cl_2, 175 MHz, 298 K) δ (ppm) = 20.07, 28.81, 29.12, 36.46, 36.69, 36.74, 39.48, 41.35, 43.26, 57.30, 109.31, 114.47, 119.15, 121.75, 124.31, 125.96, 126.98, 127.64, 129.46, 130.73, 130.94, 136.80, 139.72, 143.18, 151.55, 155.40, 161.93, 177.43.

HRMS-ASAP-TOF$^+$ *m/z* calculated for $C_{77}H_{84}N_3$ $[M+H]^+$ 1050.6665, found $[M+H]^+$ 1050.6665.

M.p. 299 °C

2. NMR spectra

2.1. 2,7-dimethylfluorenone 3a

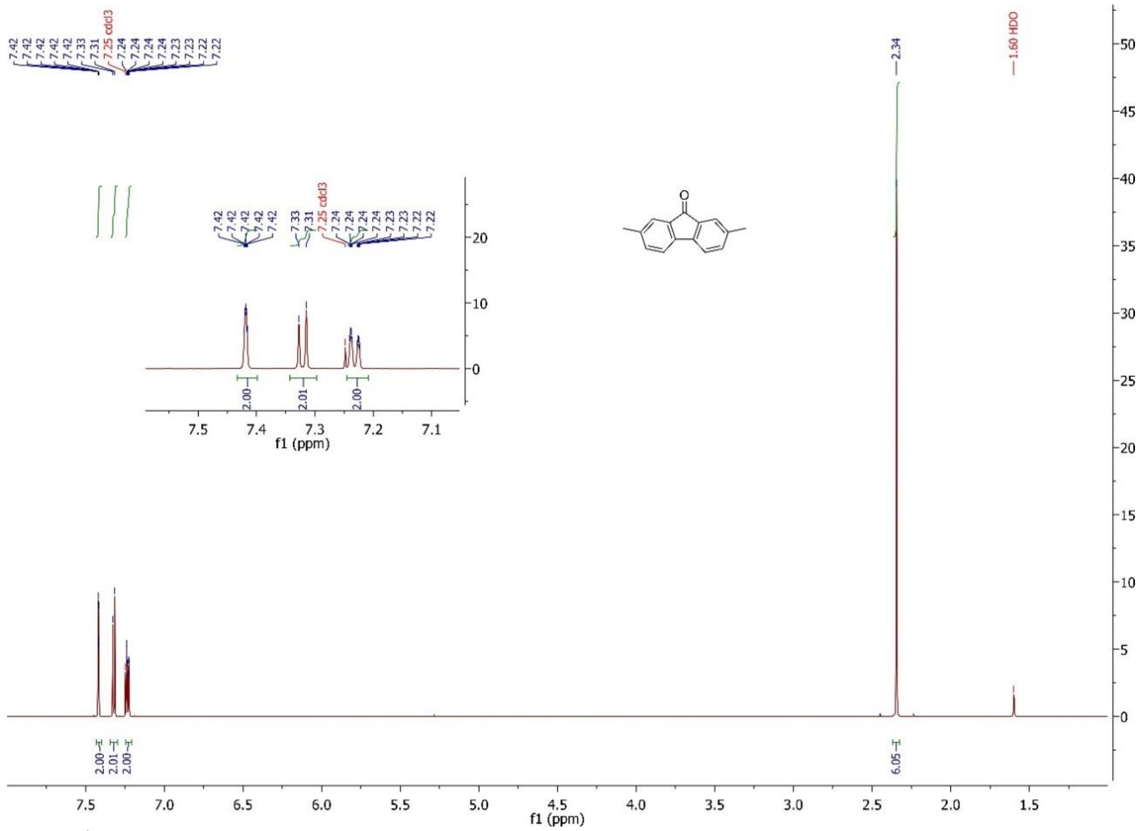

Figure S1: ^{1}H-NMR spectrum of **3a** ($CDCl_3$, 600 MHz, 298 K).

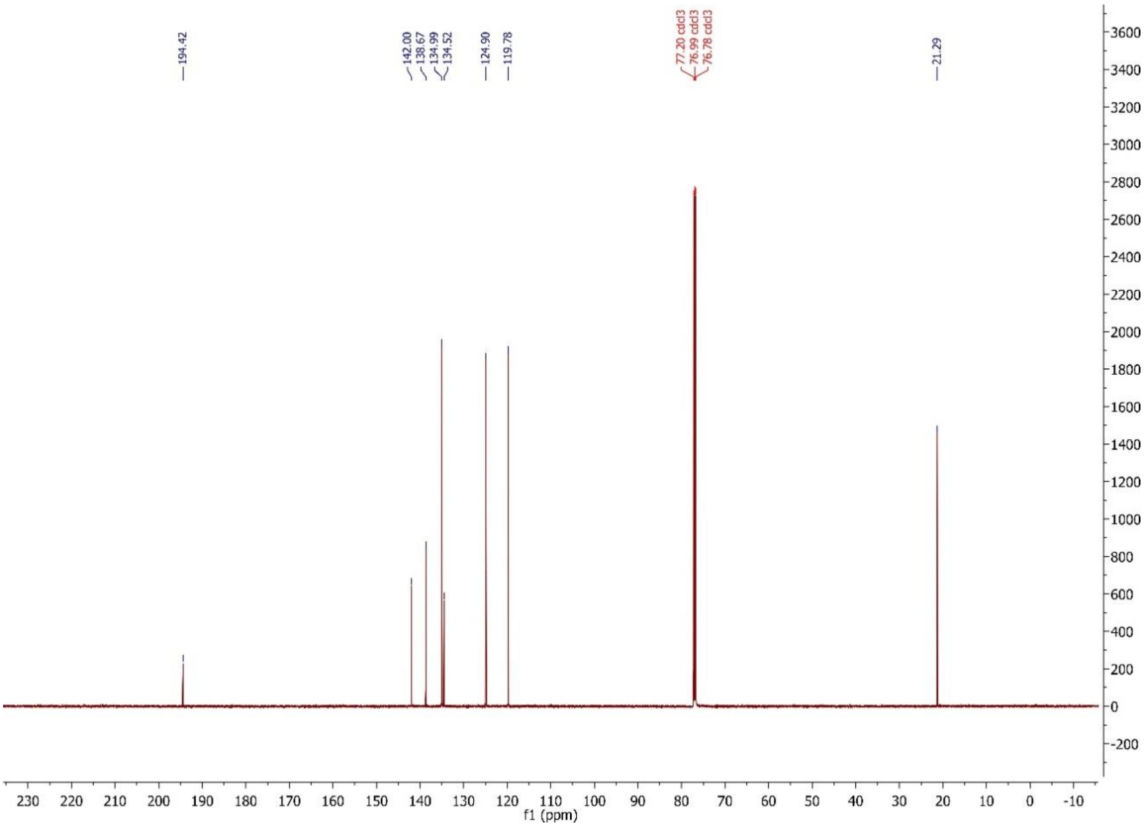

Figure S2: ^{13}C-NMR spectrum of **3a** ($CDCl_3$, 150 MHz, 298 K).

2.2. 2,7-bis-(1-adamantyl)-9*H*-fluorene 5

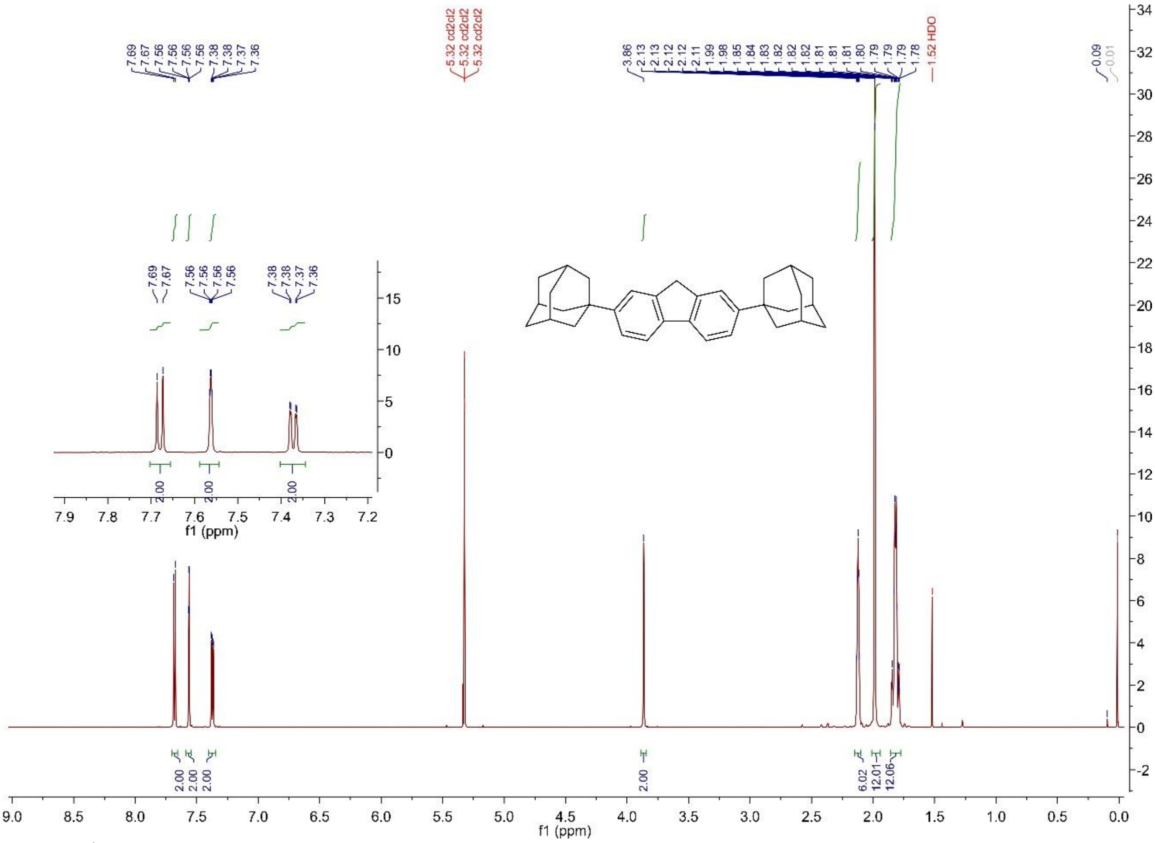

Figure S3: ^{1}H-NMR spectrum of **5** (CD_2Cl_2, 400 MHz, 298 K).

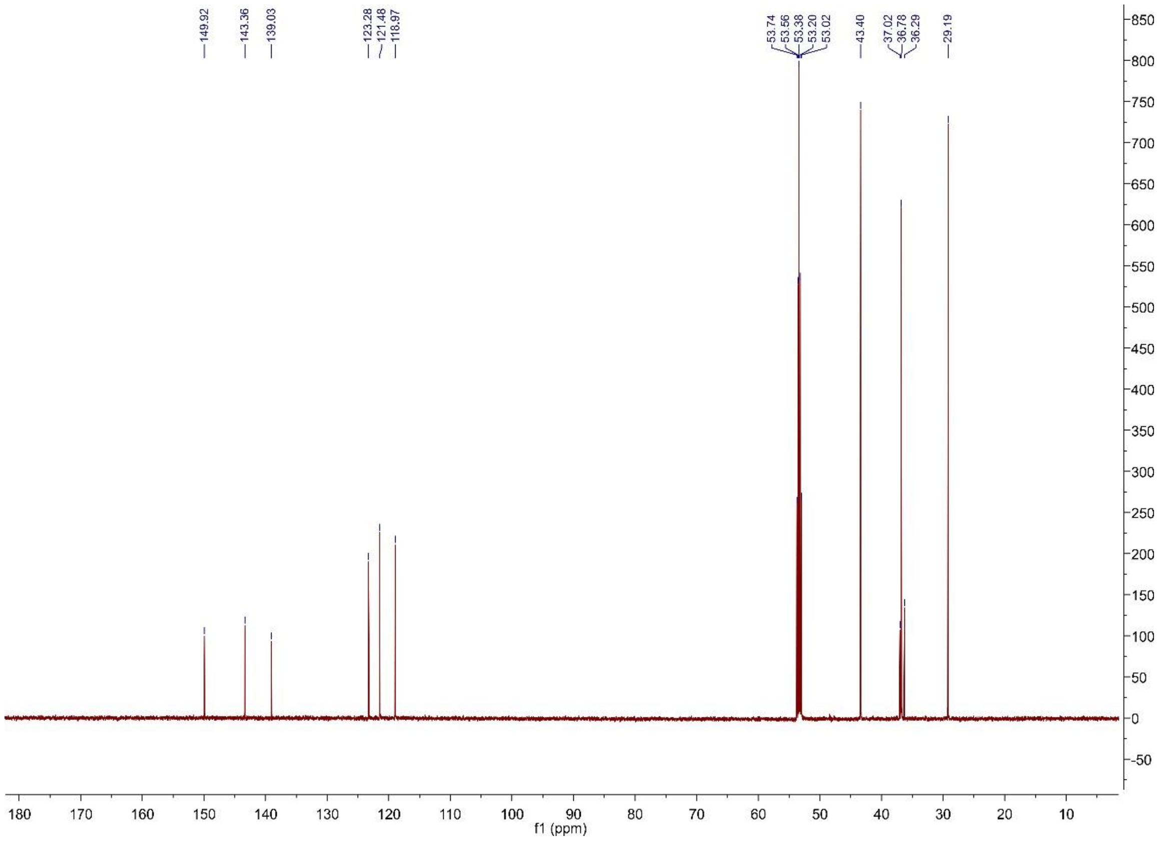

Figure S4: ^{13}C-NMR spectrum of **5** (CD_2Cl_2, 150 MHz, 298 K).

2.3. 2,7-bis-(1-adamantyl)-fluorenone 3b

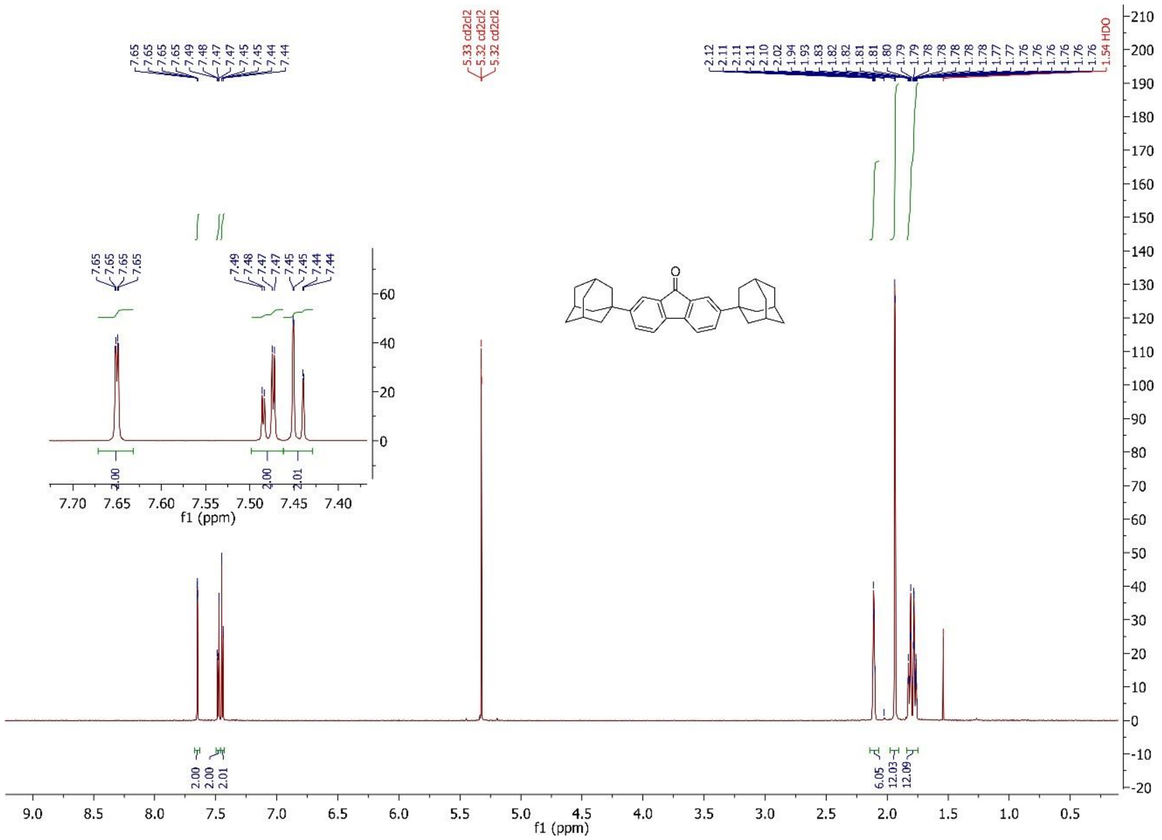

Figure S5: ^{1}H-NMR spectrum of **3b** (CD_2Cl_2, 400 MHz, 298 K).

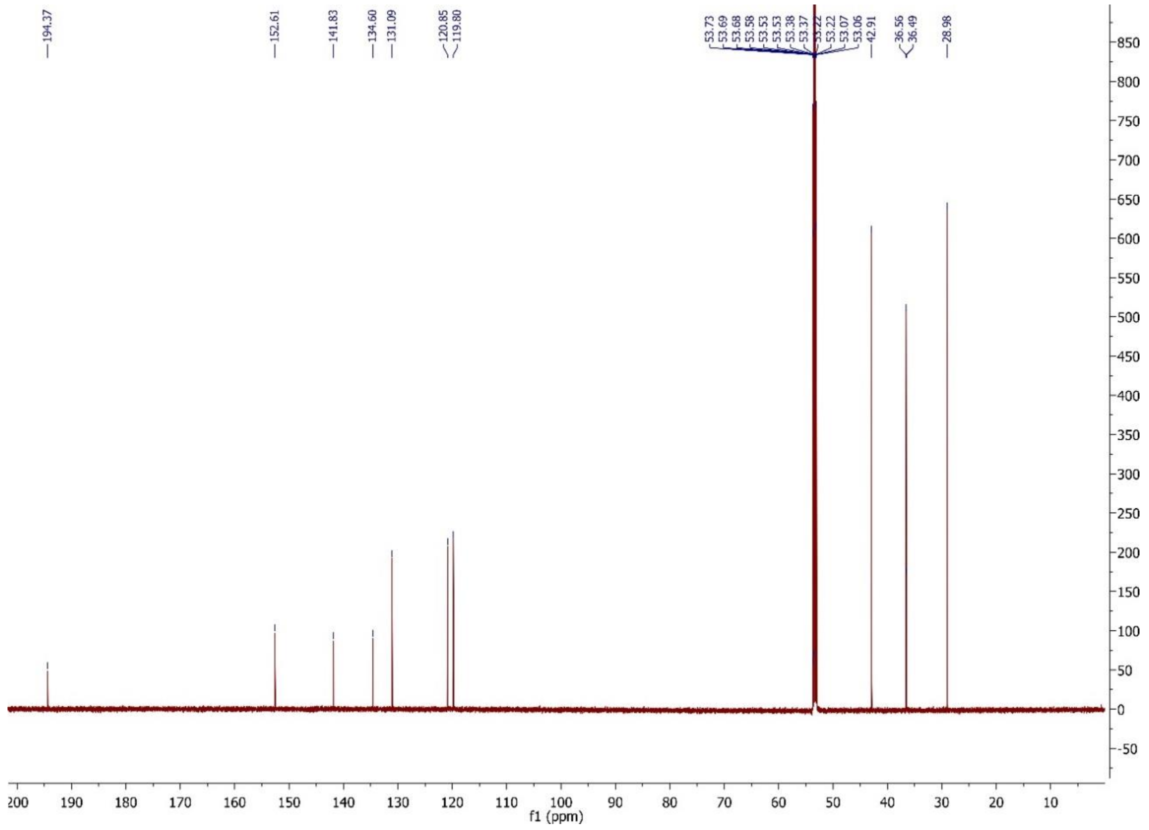

Figure S6: ^{13}C-NMR spectrum of **3b** (CD_2Cl_2, 150 MHz, 298 K).

2,2'-Tetramethyl-10*H*-spiro[acridine-9,9'-fluorene] 8a

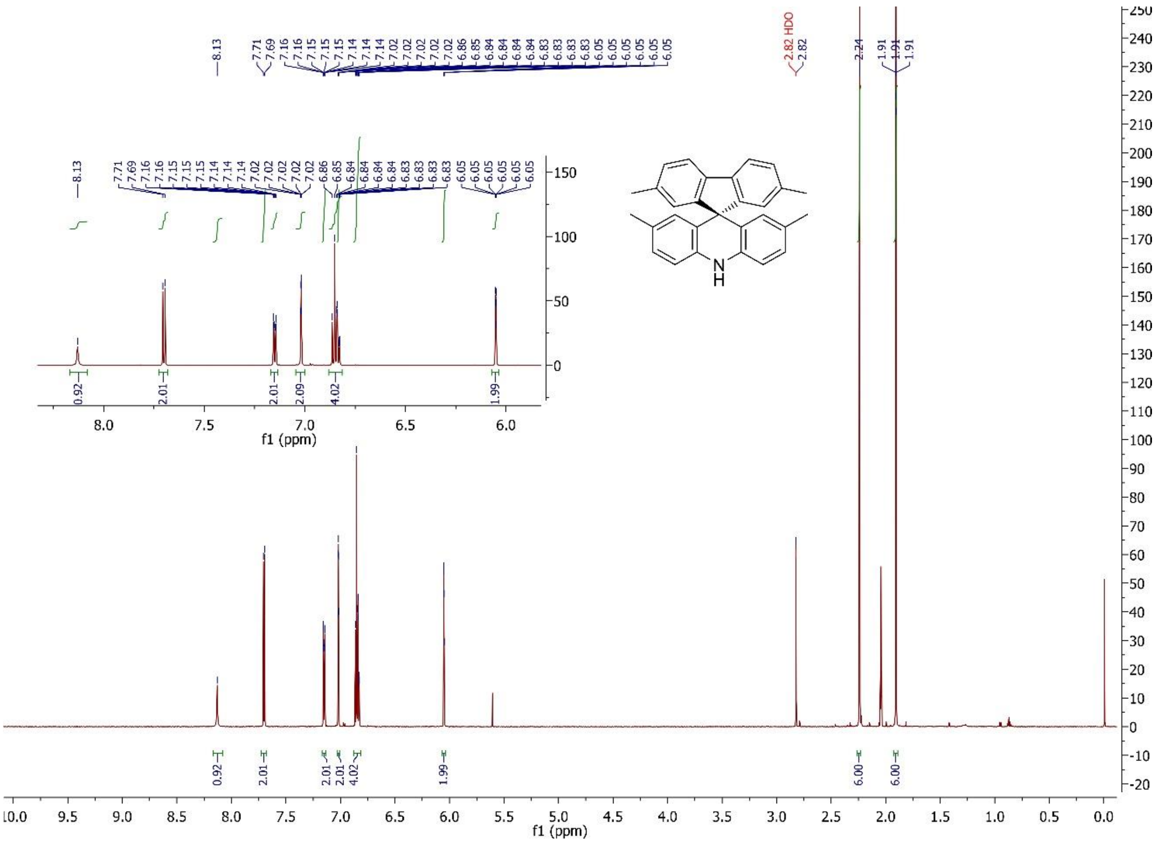

Figure S7: ^{1}H-NMR spectrum of **8a** (Acetone-d_6, 700 MHz, 298 K).

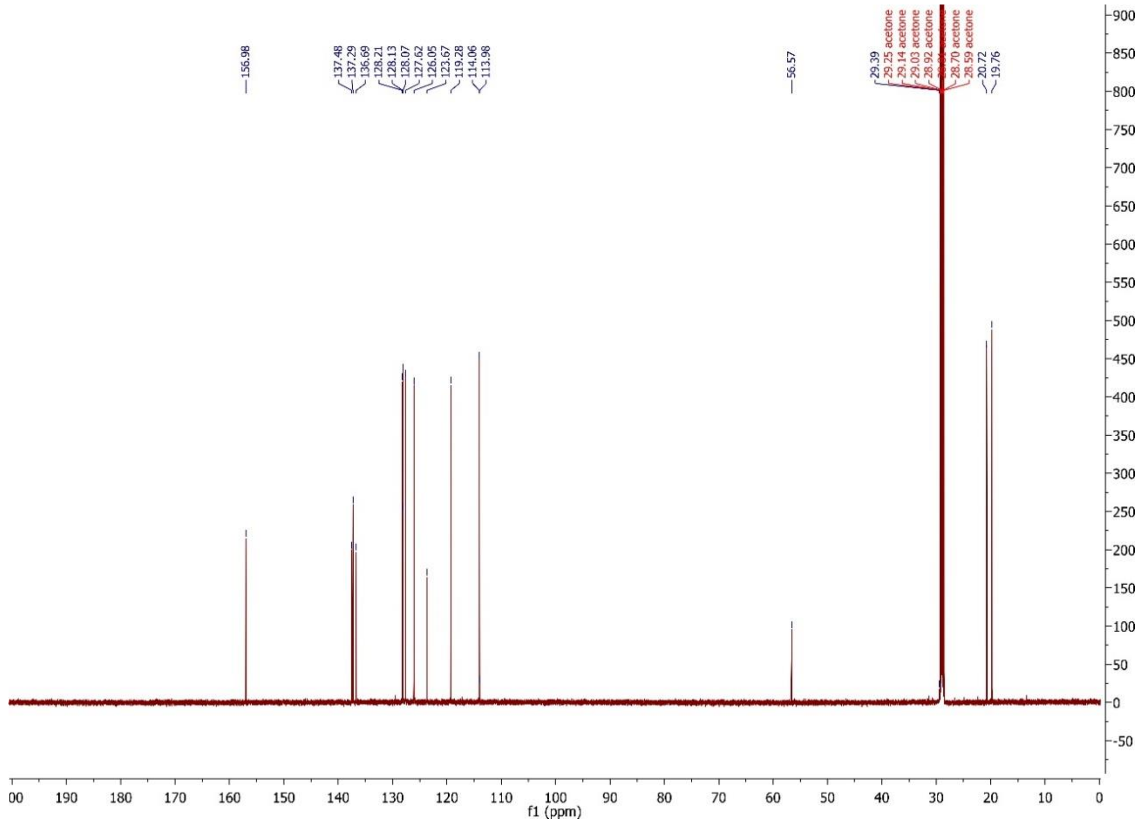

Figure S8: ^{13}C-NMR spectrum of **8a** (Acetone-d_6, 176 MHz, 298 K).

2.4. 2’,7’-Bis(adamantyl)-2,7-dimethyl-10*H*-spiro[acridine-9,9’-fluorene] 8b

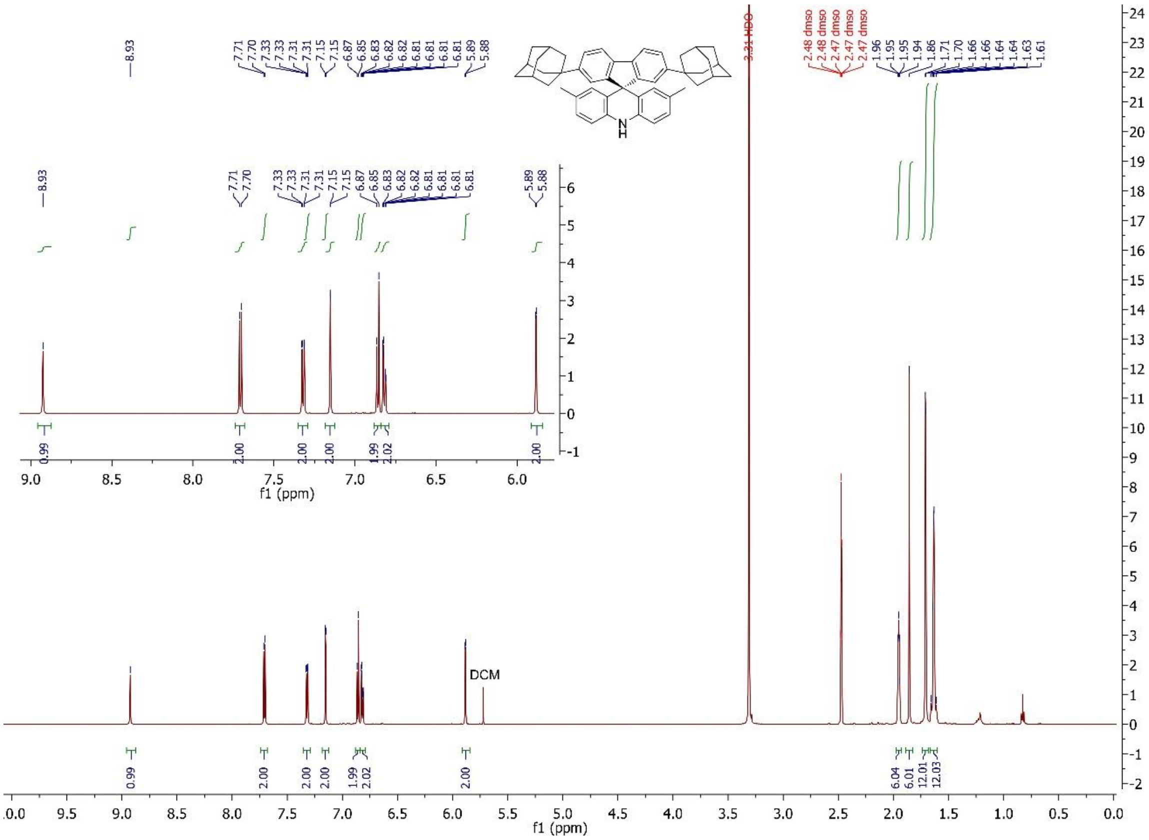

Figure S9: ^{1}H-NMR spectrum of **8b** (DMSO-d_6, 600 MHz, 298 K).

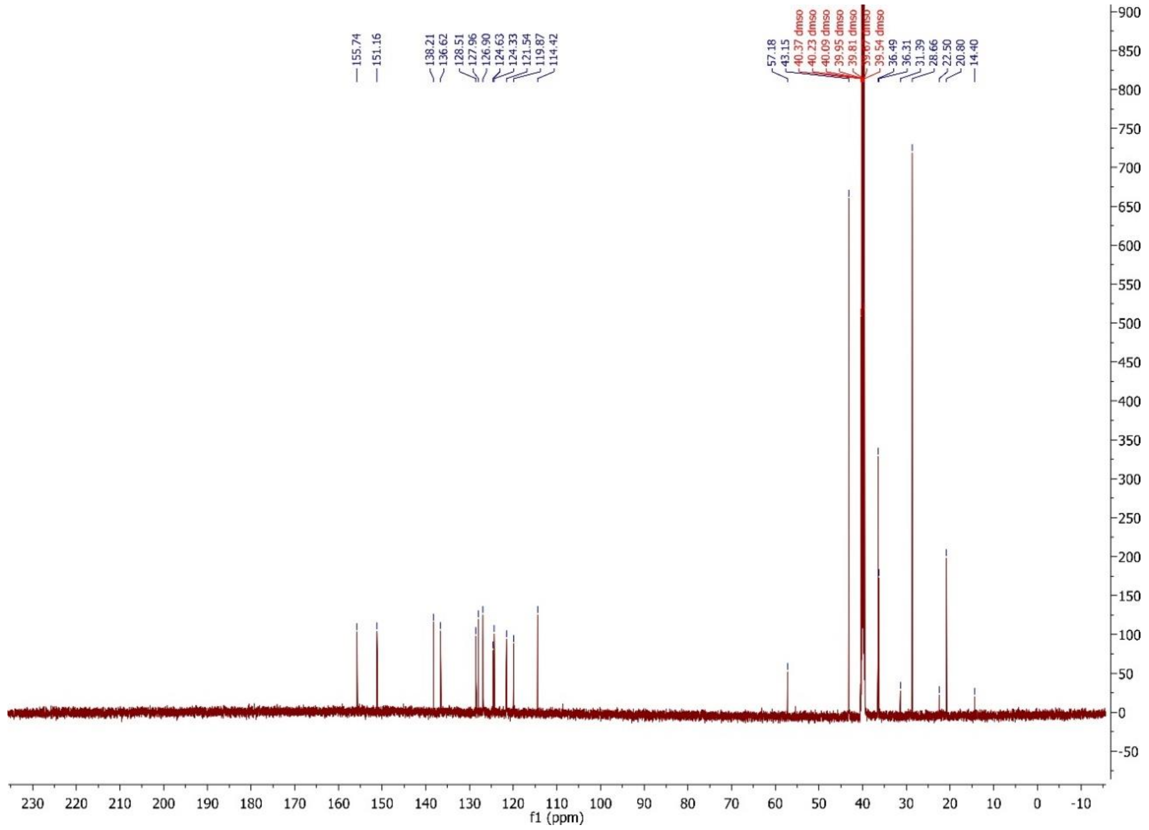

Figure S10: ^{13}C-NMR spectrum of **8b** (DMSO-d_6, 150 MHz, 298 K).

2.5. 2-(4-bromophenyl)-pyrimidine 10a

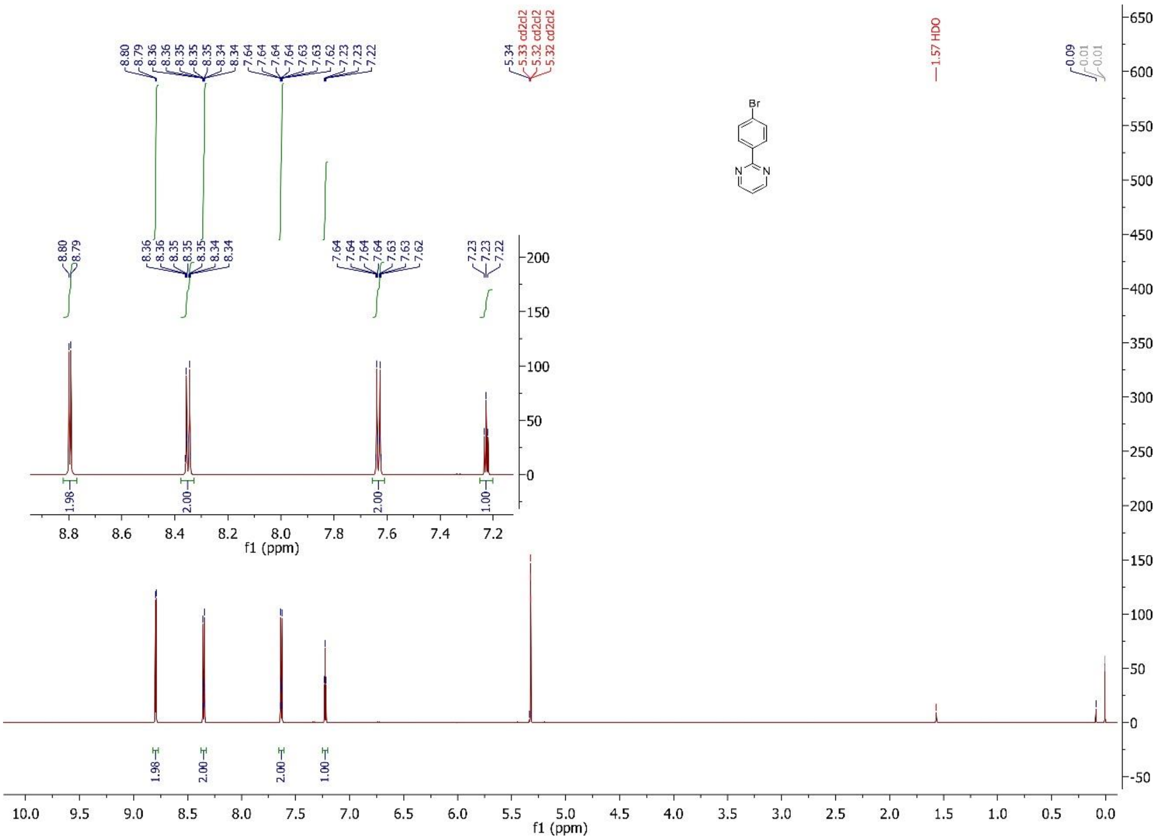

Figure S11: ^{1}H-NMR spectrum of **10a** (CD_2Cl_2, 700 MHz, 298 K).

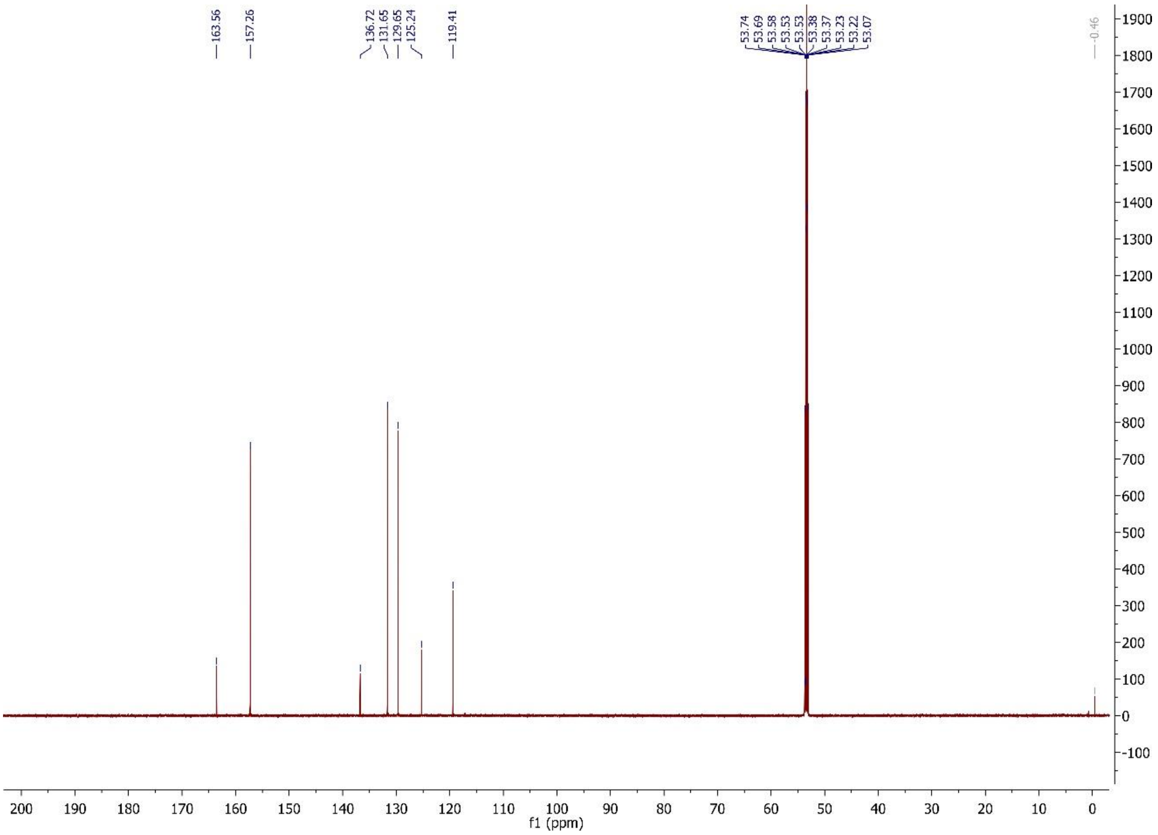

Figure S12: ^{13}C-NMR spectrum of **10a** (CD_2Cl_2, 175 MHz, 298 K).

2.6. 4,6-bis(1-adamantyl)-2-bromopyrimidine 11

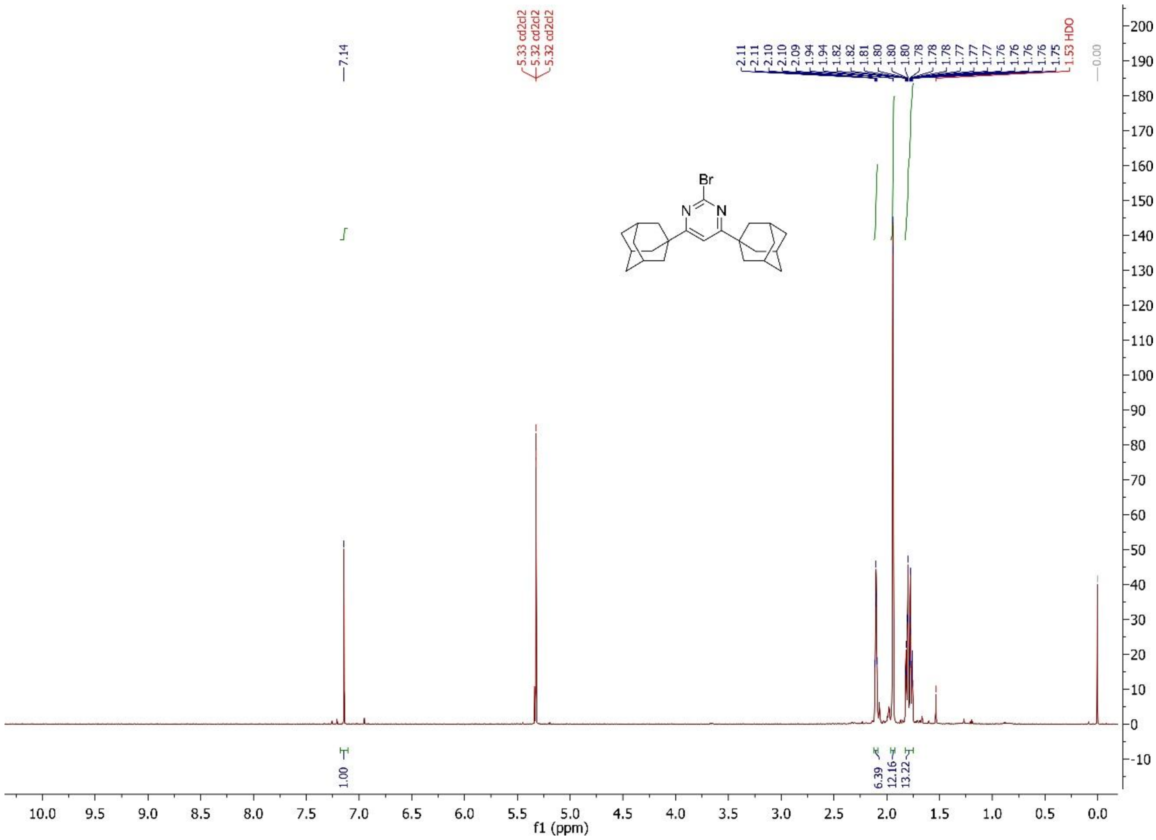

Figure S13: ^{1}H-NMR spectrum of **11** (CD_2Cl_2, 700 MHz, 298 K).

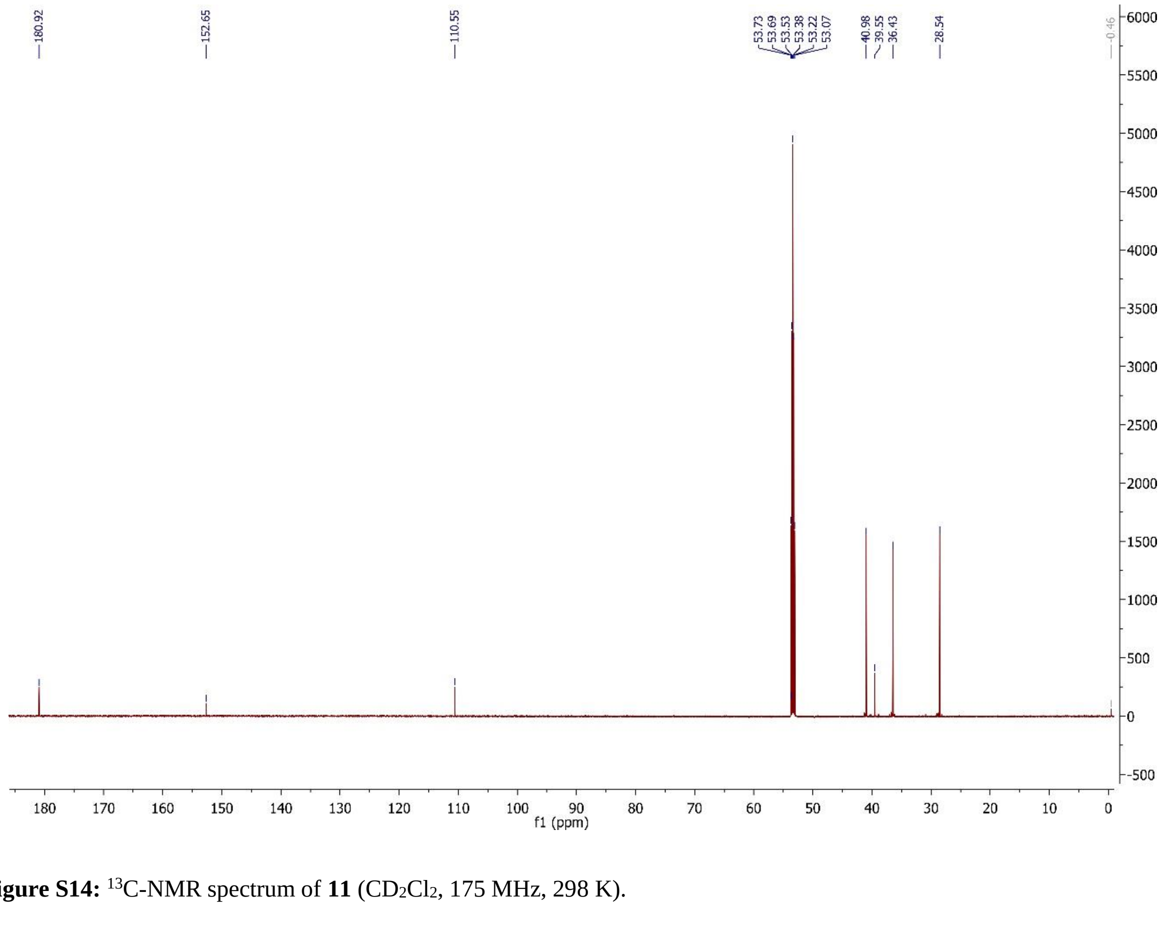

Figure S14: ^{13}C-NMR spectrum of **11** (CD_2Cl_2, 175 MHz, 298 K).

2.7. 4,6-bis(1-adamantyl)-2-(4-bromophenyl)pyrimidine 10b

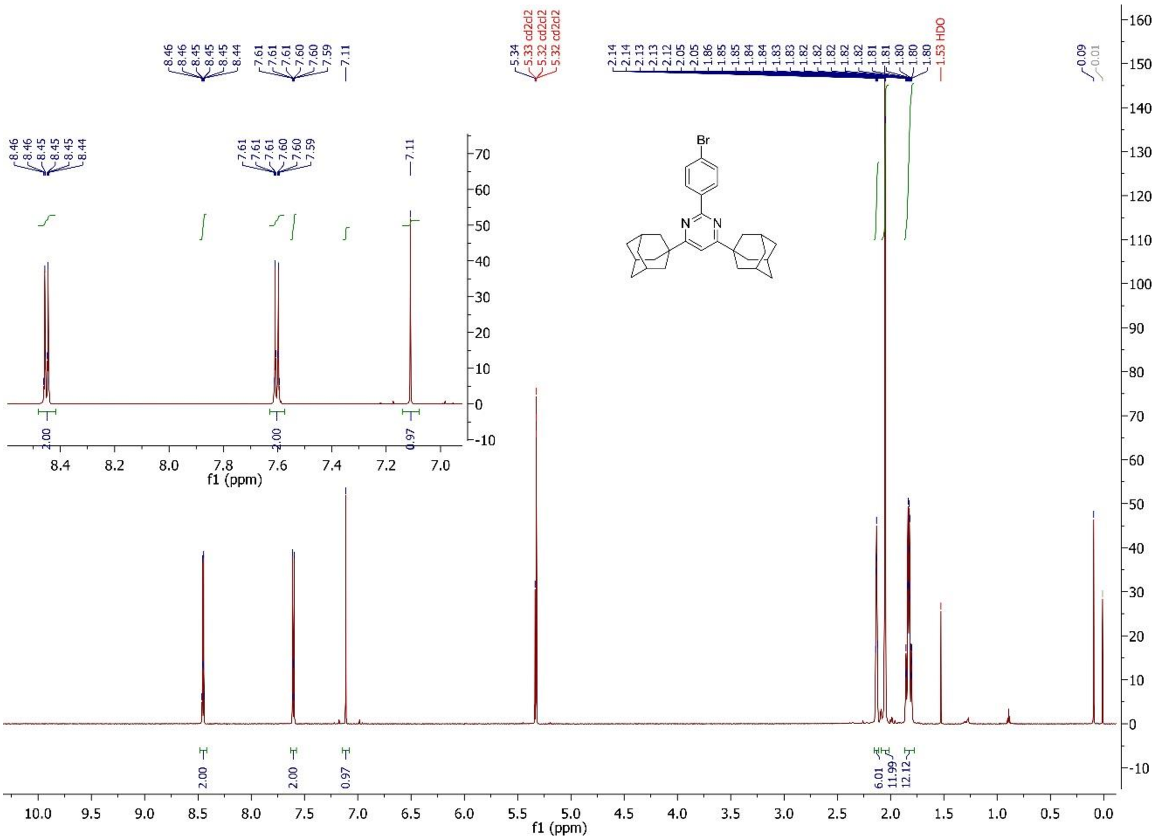

Figure S15: ^{1}H-NMR spectrum of **10b** (CD_2Cl_2, 700 MHz, 298 K).

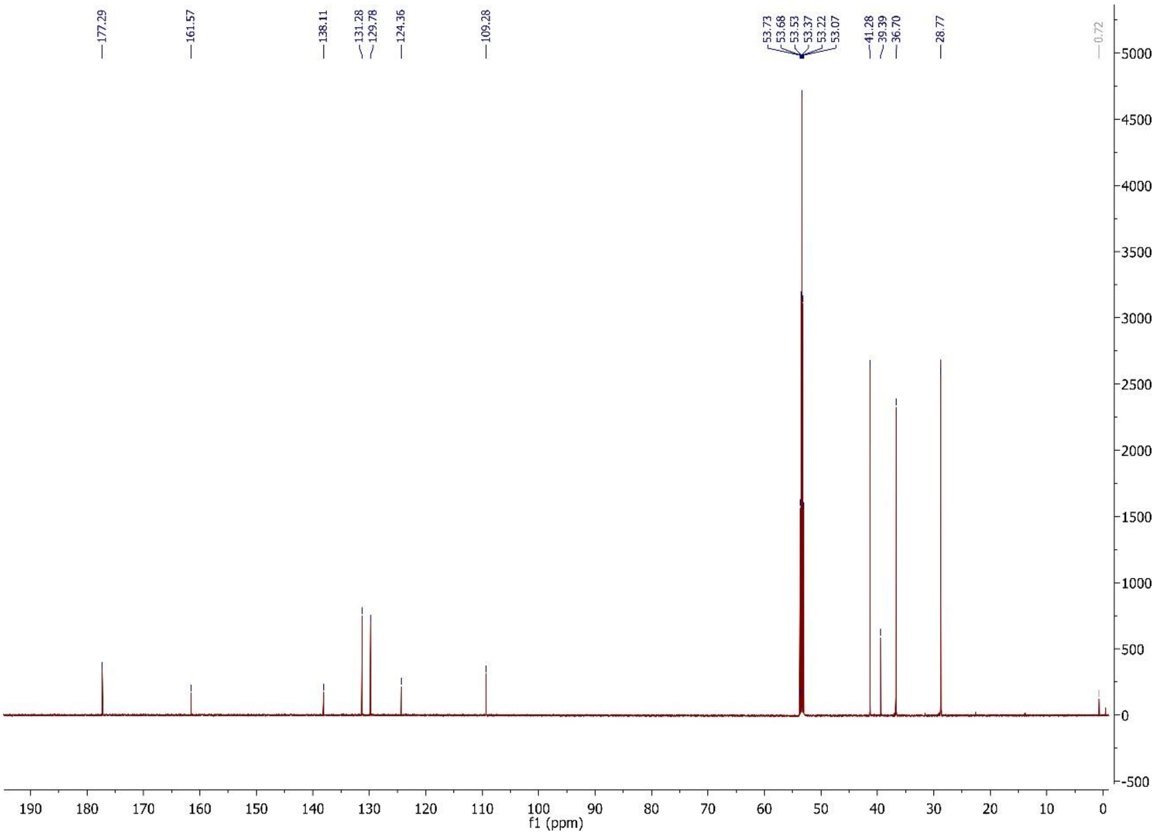

Figure S16: ^{13}C-NMR spectrum of **10b** (CD_2Cl_2, 175 MHz, 298 K).

2.8. Donor-acceptor compound 1a

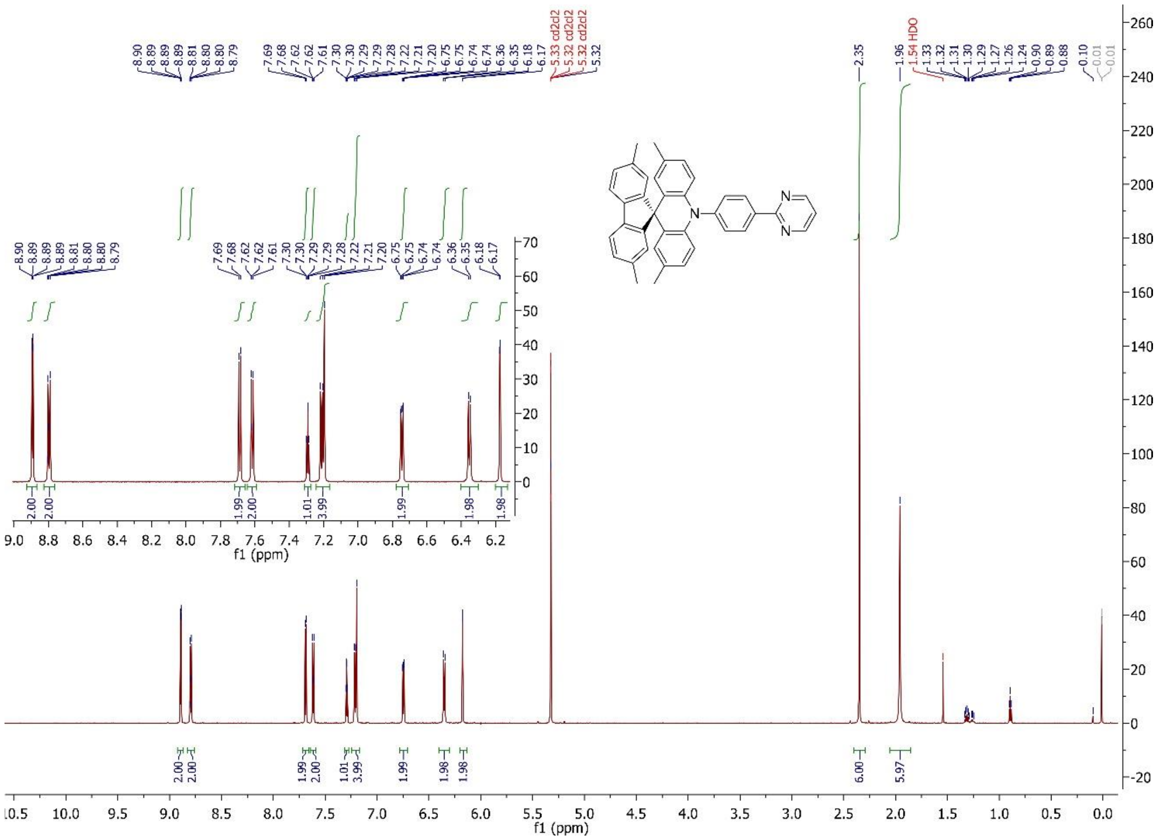

Figure S17: ^{1}H-NMR spectrum of **1a** (CD_2Cl_2, 700 MHz, 298 K).

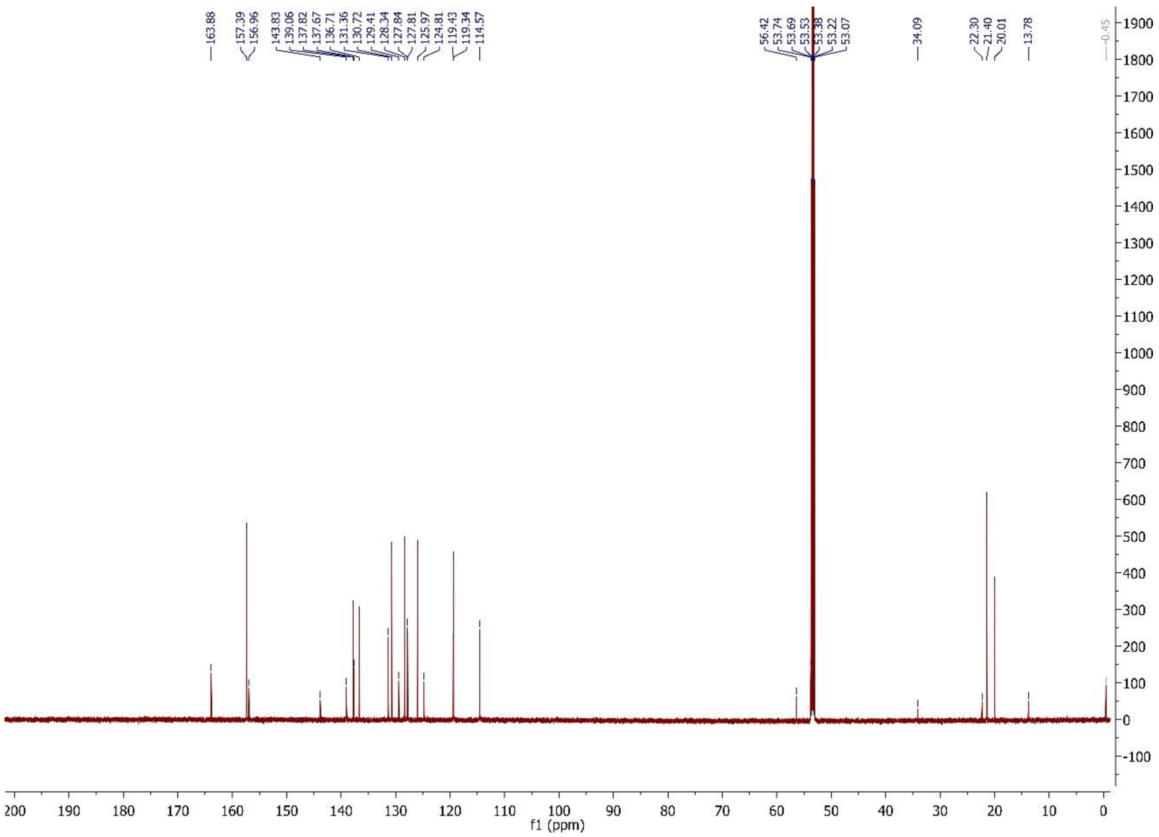

Figure S18: ^{13}C-NMR spectrum of **1a** (CD_2Cl_2, 175 MHz, 298 K).

2.9. Donor-acceptor compound 1b

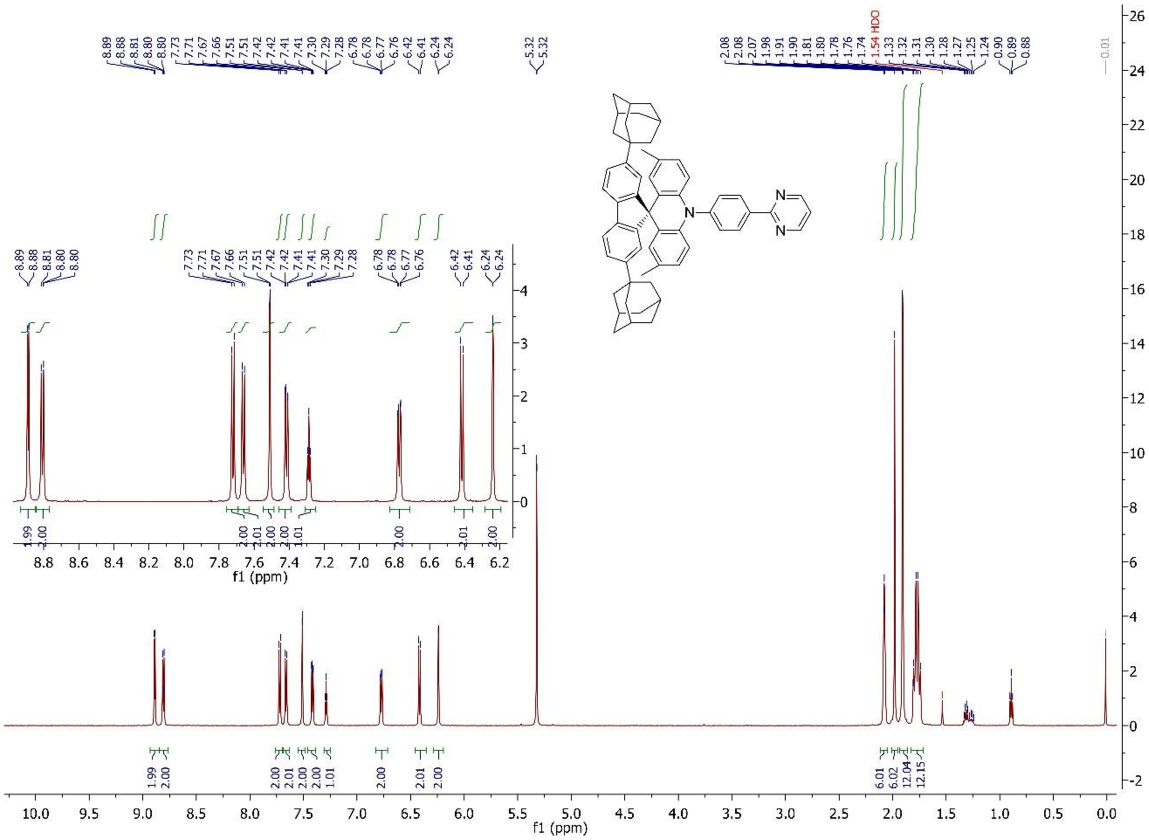

Figure S19: ^{1}H-NMR spectrum of **1b** (CD_2Cl_2, 600 MHz, 298 K).

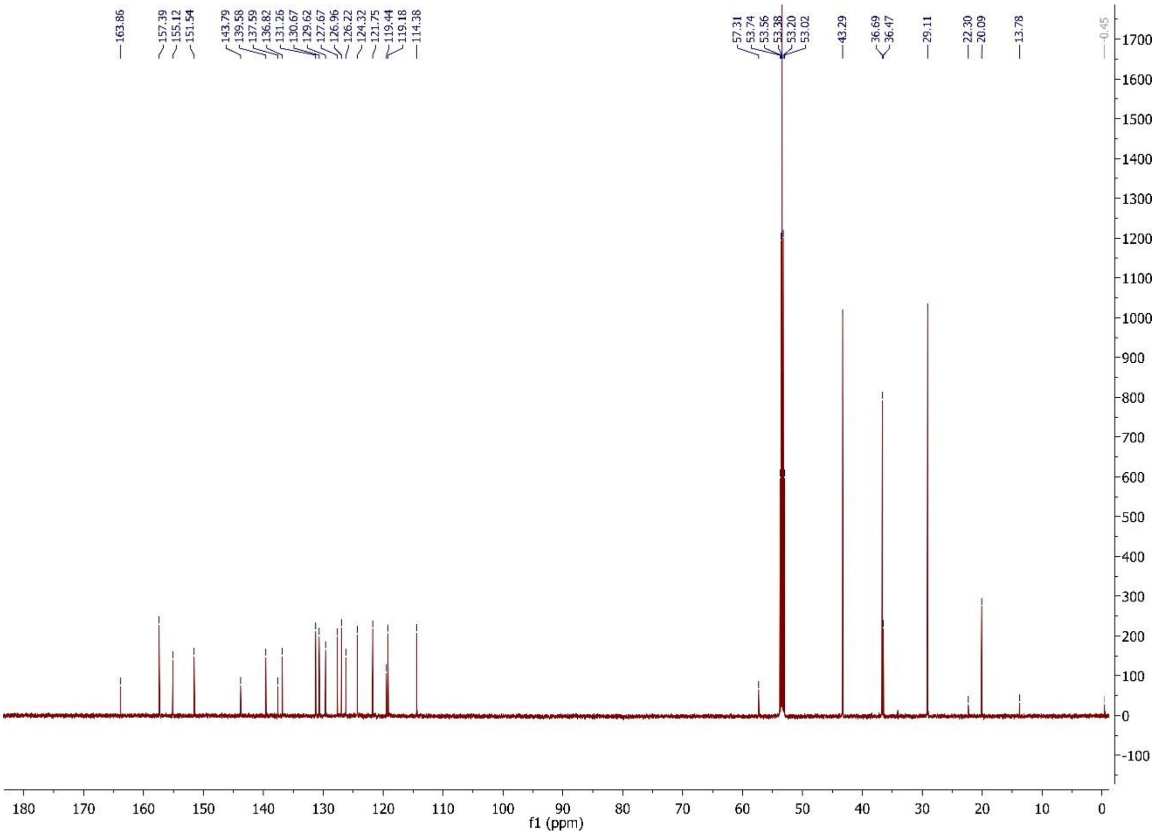

Figure S20: ^{13}C-NMR spectrum of **1b** (CD_2Cl_2, 150 MHz, 298 K).

2.10. Donor-acceptor compound 1c

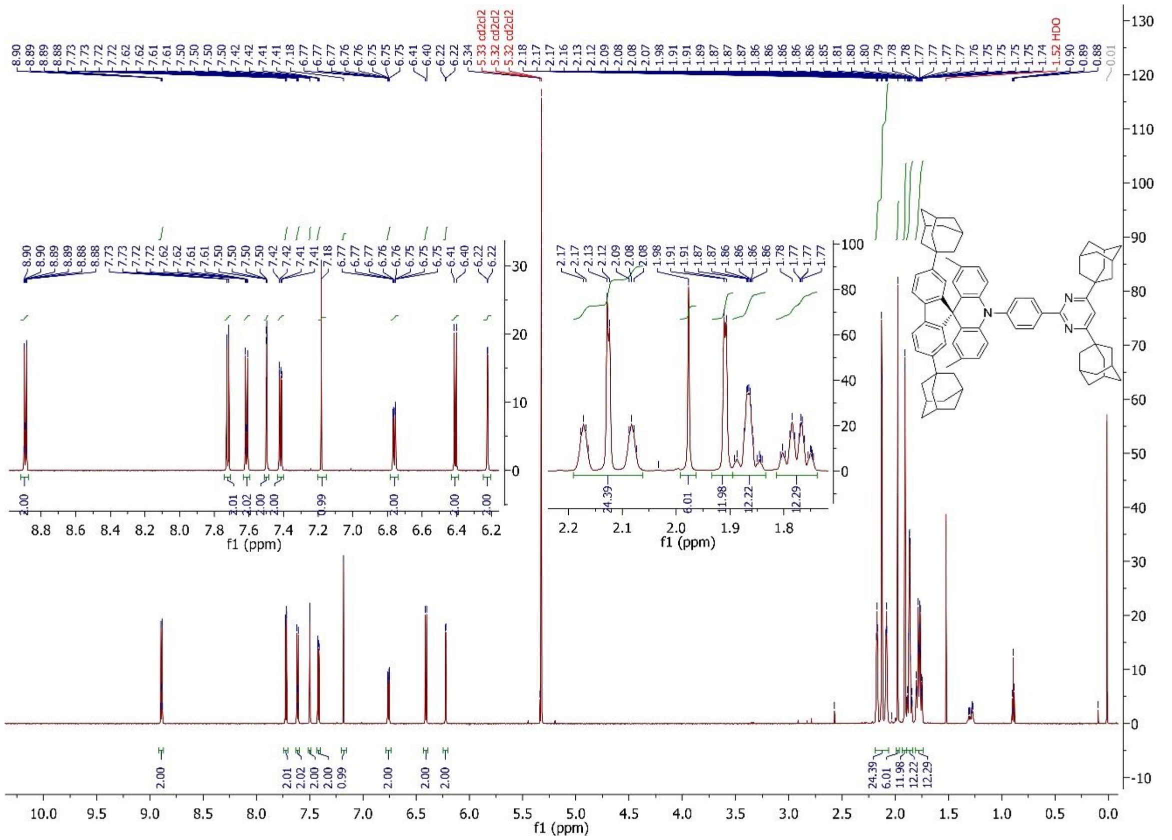

Figure S21: ^{1}H-NMR spectrum of **1c** (CD_2Cl_2, 700 MHz, 298 K).

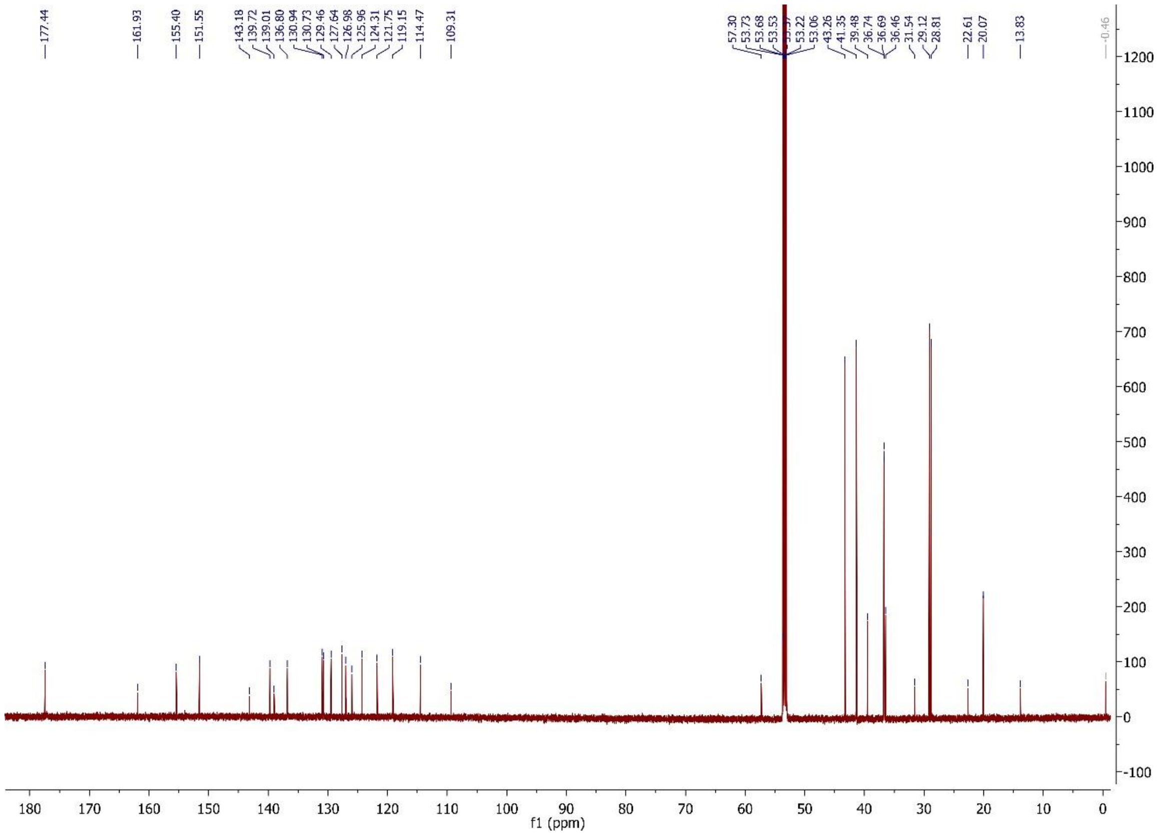

Figure S22: ^{13}C-NMR spectrum of **1c** (CD_2Cl_2, 175 MHz, 298 K).

3. VT-NMR

3.1. Donor-acceptor compound 1a

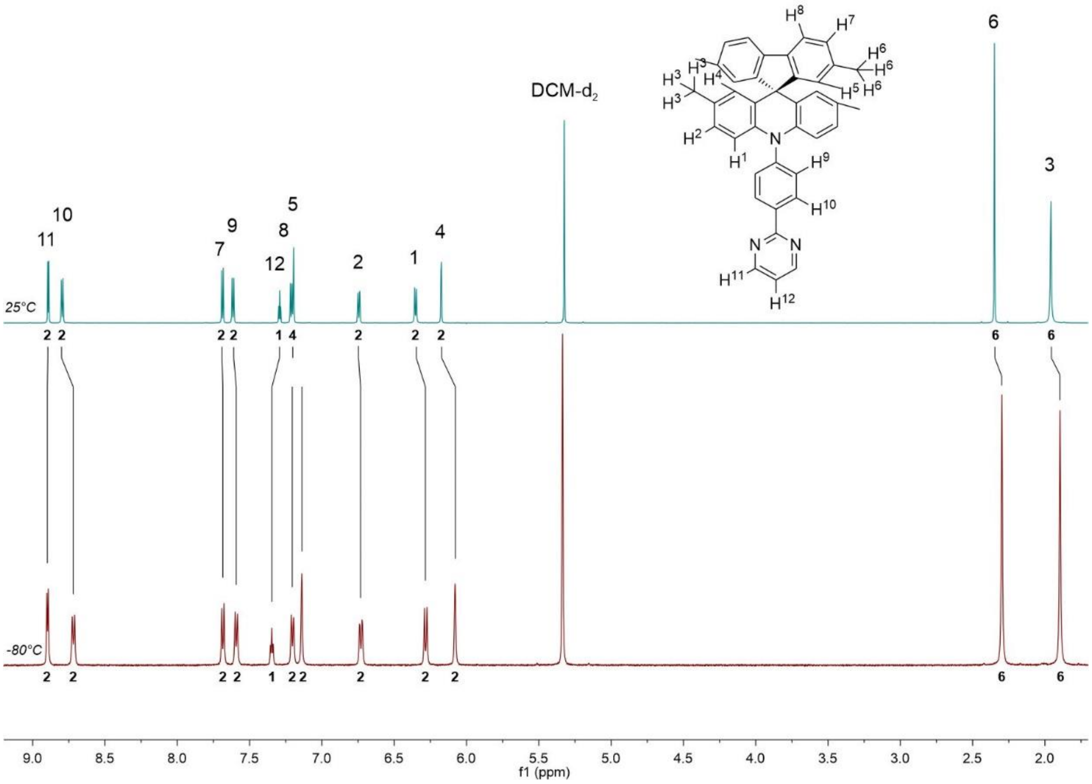

Figure S23. ^{1}H-NMR spectra of compound **1a** at 25 °C (top) and -80 °C (bottom) (CD_2Cl_2, 400 MHz).

3.2. Donor-acceptor compound 1b

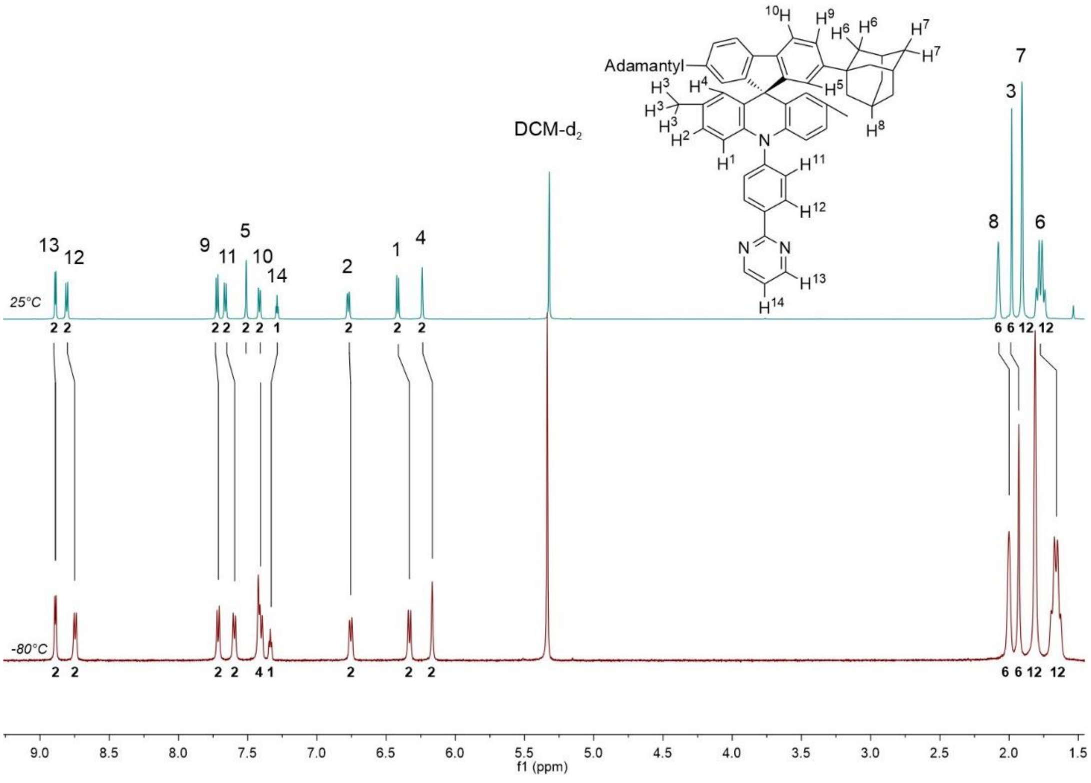

Figure S24. ^{1}H-NMR spectra of compound **1b** at 25 °C (top) and -80 °C (bottom) (CD_2Cl_2, 400 MHz).

3.3. Donor-acceptor compound 1c

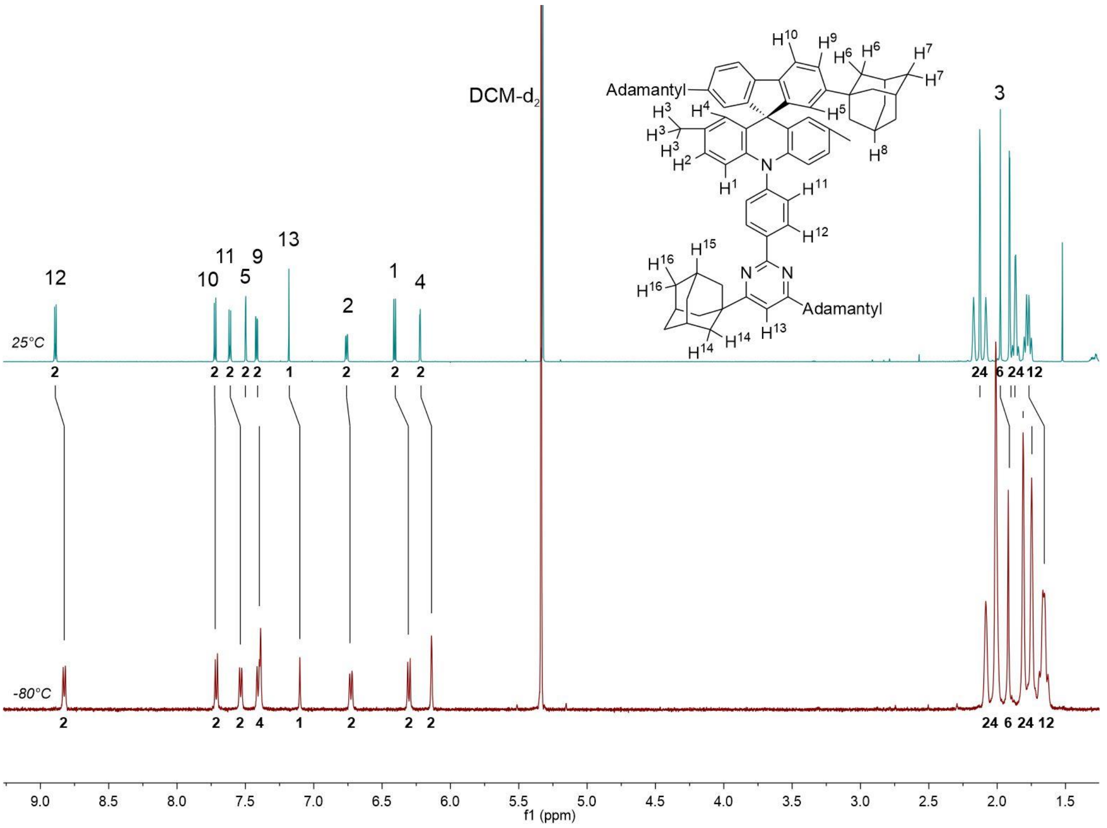

Figure S25. ^{1}H-NMR spectra of compound **1c** at 25 °C (top) and -80 °C (bottom) (CD_2Cl_2, 400 MHz).

4. Single Crystal X-Ray Analysis

Crystal data and experimental details are listed in Table S1A. Crystal **1a** was twinned by a 180° rotation about the real axis *x*, i.e. the twin law ($\bar{1}$ 0 0 0 $\bar{1}$ 0 0.2 0 1), deconvoluted using CELL_NOW 2008/4 program (G.M.Sheldrick, Bruker AXS, 2008). Component contributions were refined to 0.716(3) and 0.284(3). Crystal **1c**·3CD_2Cl_2 was an unsystematic 3-component twin, deconvoluted similarly. Only the reflections from the major component were integrated and used, the minor components (differing from the latter by 6° and 164° rotations) were ignored.

Structure **1c**·3CD_2Cl_2 contains infinite channels (totaling 648 Å^3 per unit cell, or 19% of the crystal volume) occupied by disordered CD_2Cl_2, which was masked using OLEX2 SMTBX program based on the algorithm of Rees *et al.*.[7] The calculated integral electron density in the channel (199 *e* per unit cell) corresponds to *ca*. 5 molecules of CD_2Cl_2, i.e. 2.5 molecules per formula (or asymmetric) unit. Alternatively, a model with five partly occupied and partly overlapping positions (totaling 2.15 molecules per asymmetric unit with substantial diffuse residual electron density) could be refined at atomic resolution, albeit with a higher R_1=0.118. Besides, the unit cell contains one enclosed void,

containing one CD_2Cl_2 molecule whose chlorine atoms are related by an inversion center and the CD_2 group disordered between two positions related by this center.

Table S1A. Crystal data and experimental details

Compound	**1a**	**1a**·CD_2Cl_2	**1b**·¾CD_2Cl_2	**1c**·3CD_2Cl_2
Formula	$C_{39}H_{31}N_3$	$C_{39}H_{31}N_3$·CD_2Cl_2	$C_{57}H_{55}N_3$·¾CD_2Cl_2	$C_{77}H_{83}N_3$·3CD_2Cl_2
CCDC deposition no.	2017592	2017593	2017594	2017595
$D_{calc.}$/ g cm^{-3}	1.250	1.305	1.251	1.288
μ(Mo-$K\alpha$)/mm^{-1}	0.07	0.24	0.16	0.30
Formula weight	541.67	628.60	847.24	1311.27
T/K	120	120	120	120
Crystal System	monoclinic	triclinic	triclinic	triclinic
Space Group	$C2/c$ (no.15)	$P\bar{1}$ (no. 2)	$P\bar{1}$ (no. 2)	$P\bar{1}$ (no. 2)
a/Å	14.938(2)	13.6745(5)	19.556(2)	10.9925(14)
b/Å	9.8016(14)	14.1101(5)	21.485(3)	16.709(2)
c/Å	39.344(5)	19.0600(7)	22.122(3)	19.701(3)
α/°	90	100.6759(14)	89.885(3)	104.867(3)
β/°	92.209(4)	110.3176(14)	79.230(3)	100.320(3)
γ/°	90	103.8628(14)	80.444(3)	97.559(3)
V/Å^3	5756(2)	3199.5(2)	9000.0(18)	3380.3(7)
Z	8	4	8	2
$2\theta_{max}$/°	50.3	50	50.3	50.3
Reflections total	36128	51037	116298	45036
unique	5165	11253	32145	12046
with $I\geq2\sigma(I)$	4334	7748	13856	5823
R_{int}	0.074	0.057	0.147	0.111
Parameters, restraints	389, 0	858, 26	2286, 286	753, 792
$\Delta\rho_{max,min}$ /eÅ^3	0.44, -0.36	0.97, -0.79	0.75, -0.72	0.50, -0.57
Goodness of fit	1.188	1.029	0.996	1.052
R_1, wR_2 (all data)	0.109, 0.278	0.097, 0.157	0.204, 0.217	0.195, 0.317
R_1, wR_2 [$I\geq2\sigma(I)$]	0.097, 0.266	0.061, 0.137	0.077, 0.167	0.109, 0.276

Table S1B. Summary of structural information derived from single crystal structure analyses of compounds **1a-c**: dihedral angle C–N–C–C between phenylene bridge and the acridine N atom plane (τ), dihedral angle between phenylene bridge and the pyrimidine ring (φ), folding along the N···C(spiro) vector of the acridine unit, as the interplanar angle between arene rings (θ), tilt angle of the *N*-phenylene-pyrimidine vector with respect to the long axis of the fluorene moiety (ψ). The acridine folding angle ω, used in calculations (see next section), is measured as half the difference between the two N···C(spiro)–C(fluorene) angles.

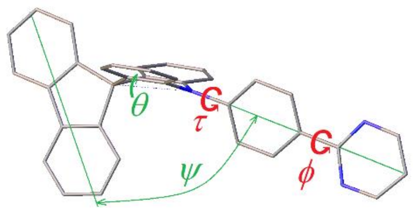

	**1a**	**1a**·CD_2Cl_2		**1b**·3/4 CD_2Cl_2				**1c**·3CD_2Cl_2
		A	*B*	*A*	*B*	*C*	*D*	
τ, °	89.5	76.6	83.5	82.8	73.4	82.2	89.2	89.5
φ, °	18.6	11.2	6.2	2.5	5.6	2.9	1.1	11.9
θ, °	19.2	21.0	26.9	25.9	30.1	18.2	30.8	12.2(24.8)*
ω, °	24.4	24.9	27.1	27.0	29.6	25.8	28.6	23.9
ψ, °	64.0	49.6	49.8	61.9	40.7	63.3	45.9	70.5

* Disordered in 2:1 ratio

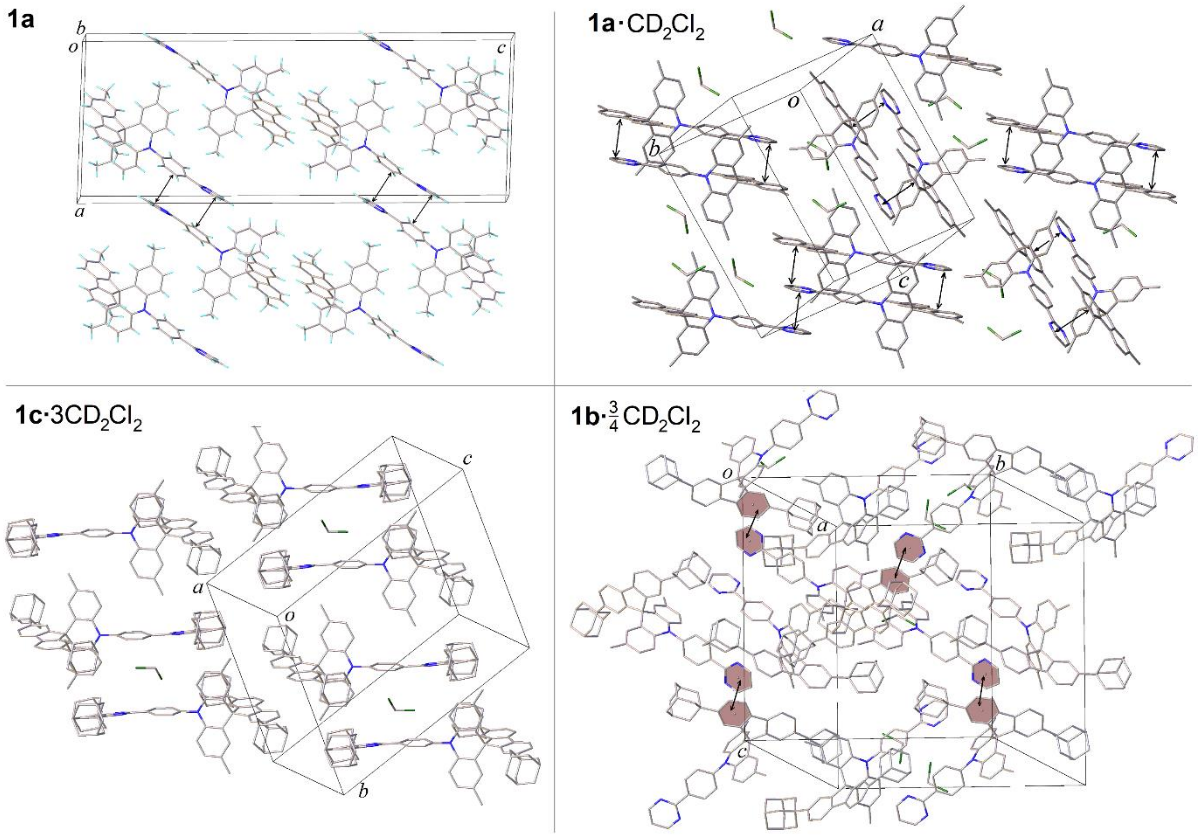

Figure S26. Crystal packing patterns of **1a**, **1a**·CD_2Cl_2, **1b**·¾CD_2Cl_2 and **1c**·3CD_2Cl_2. Arrows indicate π-π stacking contacts. H atoms (except in **1a**) and disorder are omitted.

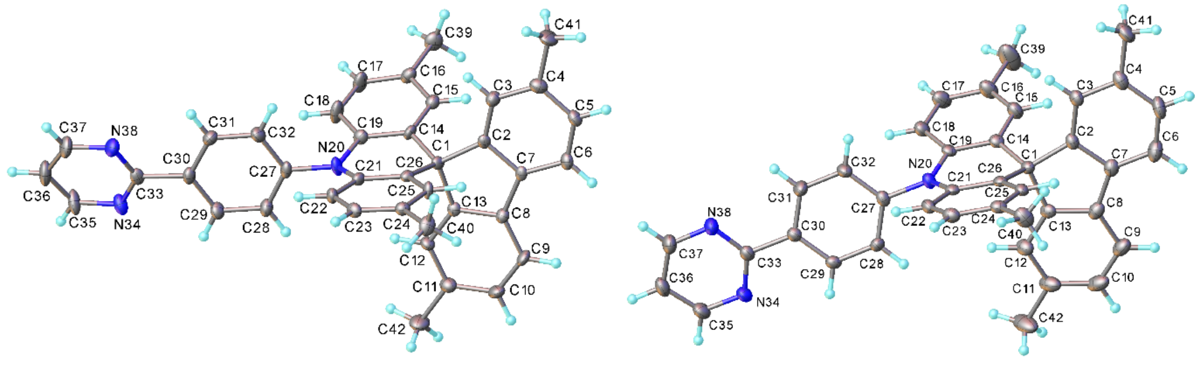

Figure S27. Molecular structure of **1a** in pure (left) and **1a**·CD_2Cl_2 (right) crystal forms. Thermal ellipsoids are drawn at the 50% probability level. Two independent molecules in the structure **1a**·CD_2Cl_2 have similar conformations.

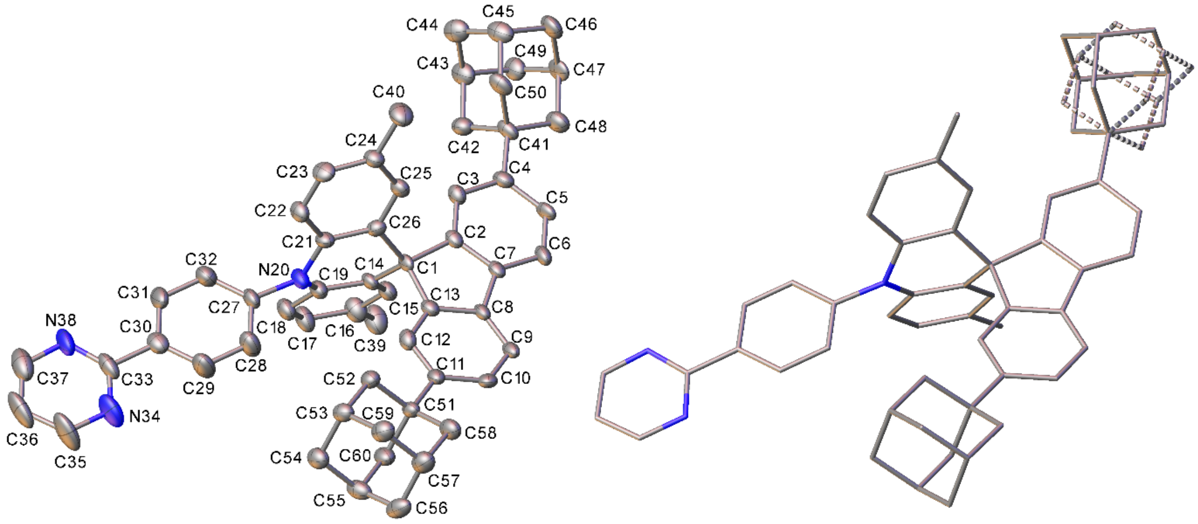

Figure S28. One of the four independent molecules (molecule *D*) in the crystal of **1b**·¾CD2Cl2 (left, thermal ellipsoids are drawn at the 50% probability level) and the rotational disorder of an adamantyl group in it (right, 0.661(3):0.339(3) occupancy ratio). H atoms are omitted for clarity.

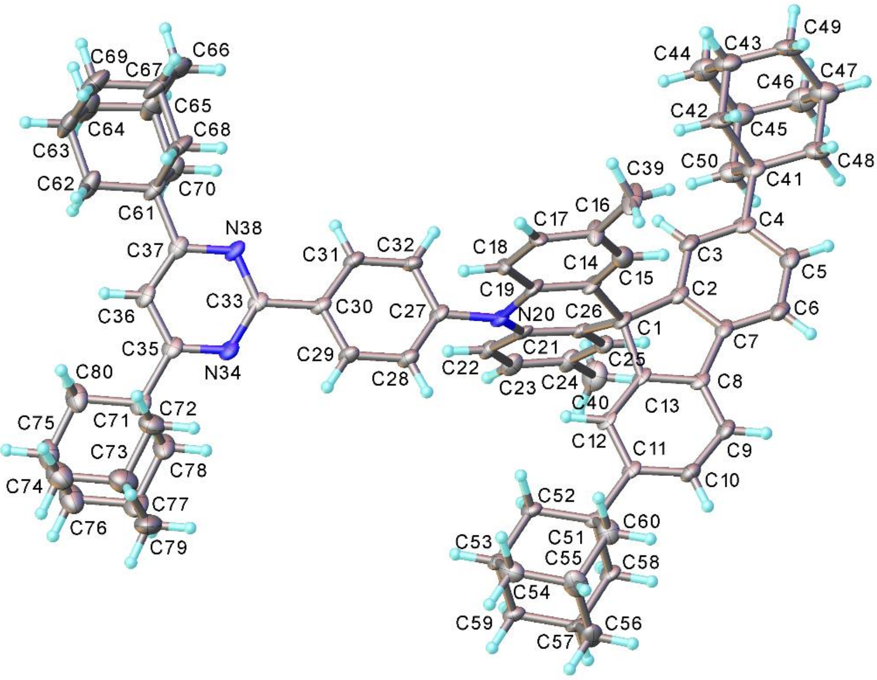

Figure S29. Molecular structure of **1c** in the crystal of **1c**·3CD2Cl2.Thermal ellipsoids are drawn at the 50% probability level.

5. Calculations

Geometry optimizations were performed with the Gaussian 16 package.[8] Ground state (S_0) geometries for **1a** were fully optimized without symmetry constraints with HF, BLYP[9-10], B3LYP, CAM-B3LYP[11] and BMK[12-14] to determine which SCF method is appropriate for modeling ground state geometries by comparing with experimental geometries of **1a**. All fully optimized S_0 geometries were found to be true minima based on no imaginary frequencies found and their Gibbs free energies determined from frequency calculations. None of these model chemistries reproduced the X-ray geometries with folded acridines. A computationally intensive post-SCF method, second order Møller-Plesset (MP2), gave a minimum in excellent agreement with experimental data. Best geometry fittings using the Olex2 package[15] revealed a root mean square (rms) error of only 0.305 Å between the geometries from MP2 and experimental whereas a rms error of 1.076 Å was determined from CAM-B3LYP and experimental.

The popular B3LYP functional (and indeed many other pure/hybrid density functional theory (DFT) methods with zero/low Hartree-Fock (HF) wave contributions) is known to significantly underestimate charge transfer (CT) energies with respect to local excitation (LE) energies.[16] The Coulomb-attenuating method (CAM-B3LYP) addresses this discrepancy and has been employed in many computational studies investigating the CT energies of donor-acceptor molecules.[17-21]

Singlet excited state (S_1) geometries were optimized using the TD OPT command (TD-DFT) from fully optimized S_0 geometries as starting geometries at CAM-B3LYP/6-31G(d). The larger HF contribution in CAM-B3LYP means that all computed transition energies are generally overestimated in time-dependent density functional theory (TD-DFT) calculations at CAM-B3LYP.[22] The parameter μ in CAM-B3LYP determines the balance of DFT to HF exchange at the intermediate point in the long-range exchange interaction.[11] If $\mu = 0$, the long-range-corrected (LC) DFT calculation corresponds to the pure (non-LC) DFT calculation, and conversely $\mu = \infty$ corresponds to the standard HF calculation.[23] The parameter μ in CAM-B3LYP is 0.33 and, to lower the HF contribution, this parameter is adjusted to 0.27 for TD-DFT computations here and elsewhere for direct comparison with experimental emission data.[24-29] The predicted absorption spectra were produced visually from six lowest singlet and triplet states on optimized ground state (S_0) geometries determined by TD-DFT. No oscillator strengths could be obtained for triplet state transitions within the TD-DFT setup.

The six lowest singlet and six lowest triplet transitions were predicted from TD-DFT on the optimized S_1 geometries with the state-specific corrected linear response polarization continuum model (cLR-PCM).[30-31] Natural transition orbital (NTO) calculations were performed on the optimized S_1 geometries to visualize the hole and particle orbitals. The NTO figures were generated using the Gabedit package.[32] The %CT values were derived by i) defining the atoms for donor and acceptor units, ii) calculating the %donor and %acceptor values in each molecular orbital using electronic structure calculations and iii) calculating the %CT from NTO orbitals using the TD-DFT generated data and the %donor and

%acceptor values with GaussSum software.[33] Spin-orbit coupling matrix elements (SOCME) were obtained from TD-DFT computations at CAM-B3LYP with def2/J TZVP as basis set for the five lowest singlet and five lowest triplet transitions using the Orca package.[34]

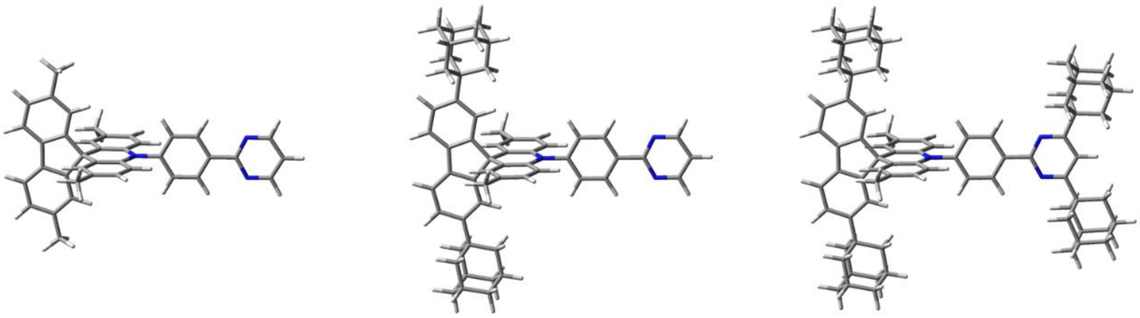

1a **1b** **1c**

Figure S30. The most stable optimized S_0 ground state geometries for **1a-1c** at CAM-B3LYP/6-31G(d).

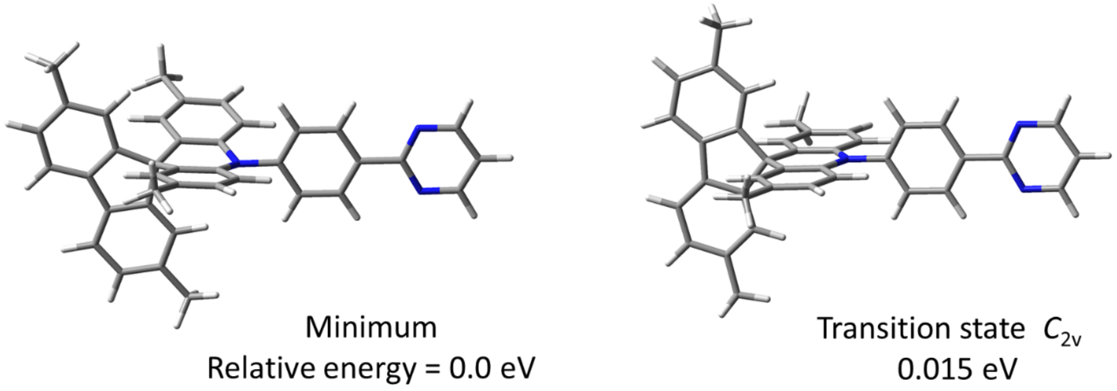

Figure S31. The most stable optimized S_0 ground state geometry and a transition state with C_{2v} symmetry for **1a** at MP2/6-31G(d). Folding angle at MP2/6-31G(d) is ω = 27.0° in agreement with ω = 24.4-27.0° found in X-ray geometries of **1a**.

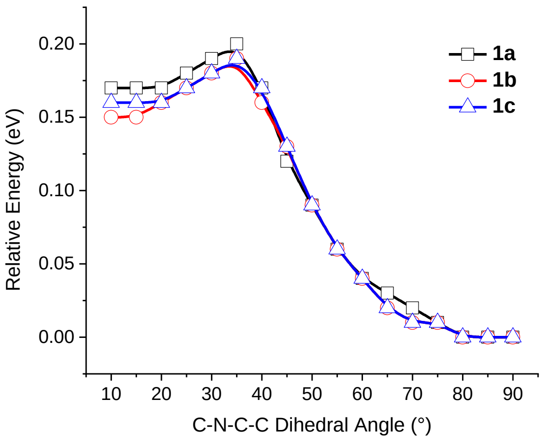

Figure S32. Graph showing relative energies with respect to fixed C-N-C-C dihedral angles related to D-A bond rotation for **1a-1c** at CAM-B3LYP/6-31G(d).

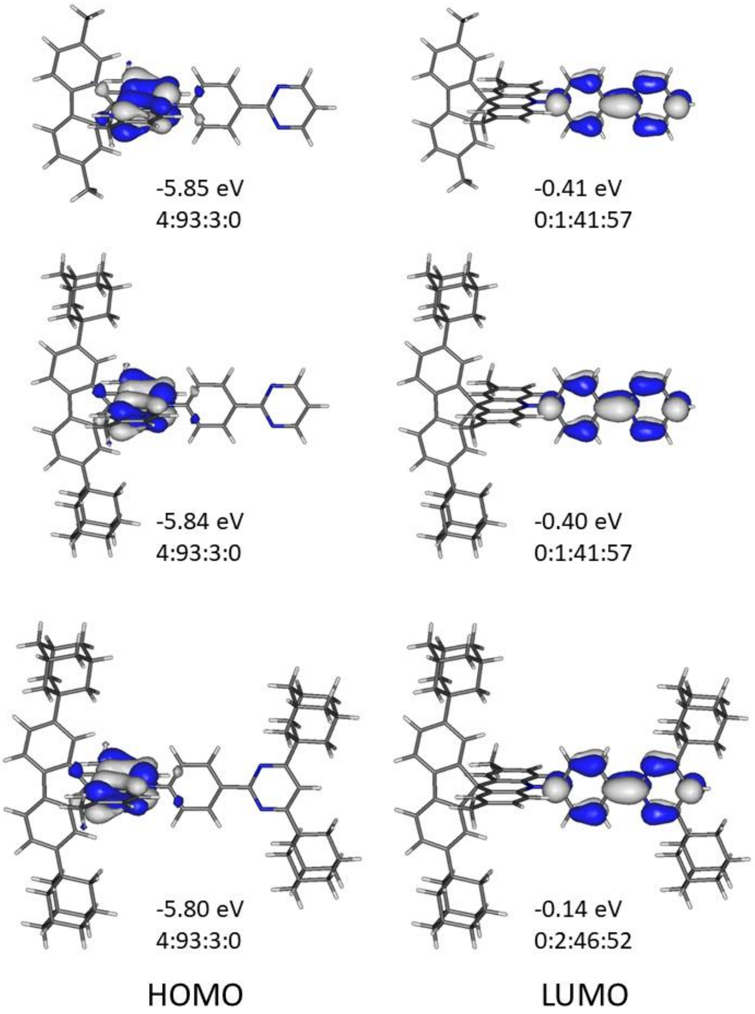

Figure S33. Frontier orbitals of **1a-1c** with MO energies and %MO orbital contributions from fluorenyl:acridine:phenylene:pyrimidyl groups. Contours in MOs are drawn at ±0.04 $(e/bohr^3)^{1/2}$.

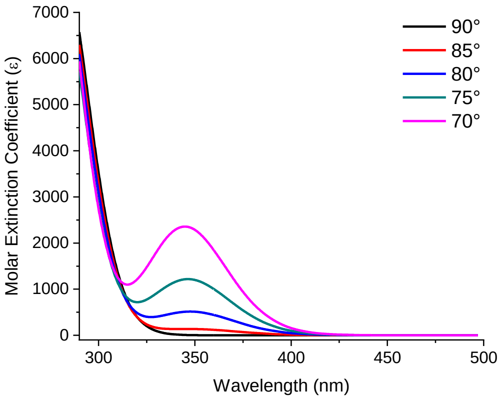

Figure S34. Simulated absorption spectra from S_0 geometries of **1a** with fixed C-N-C-C dihedral angles between 70 and 90°. The gaussian bands are simulated using a half-height width of 0.25 eV. The coefficients are calculated from the oscillator strengths times 240000.

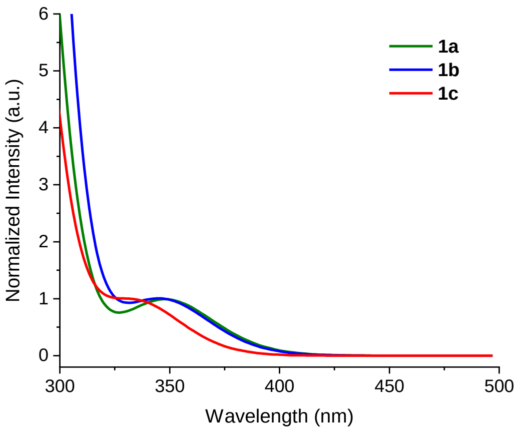

Figure S35. Simulated absorption spectra from S_0 geometries of **1a**-**1c** with C-N-C-C dihedral angles (τ) at 80°. The gaussian bands are simulated using a half-height width of 0.25 eV.

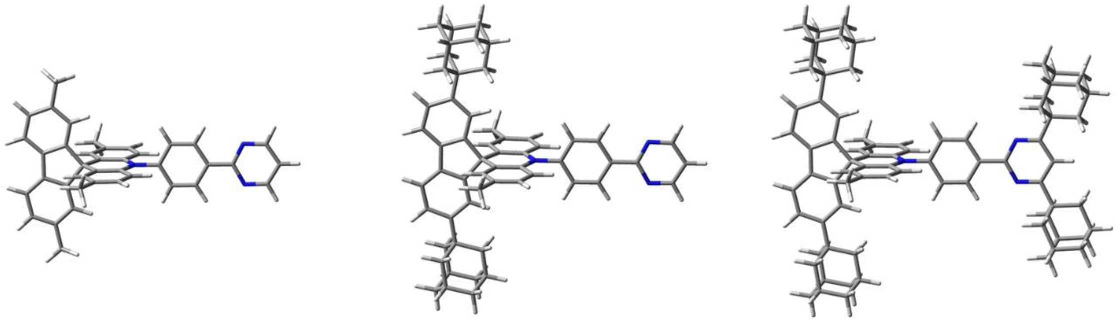

1a **1b** **1c**

Figure S36. Optimized S_1 excited state geometries for **1a**-**1c** at CAM-B3LYP/6-31G(d).

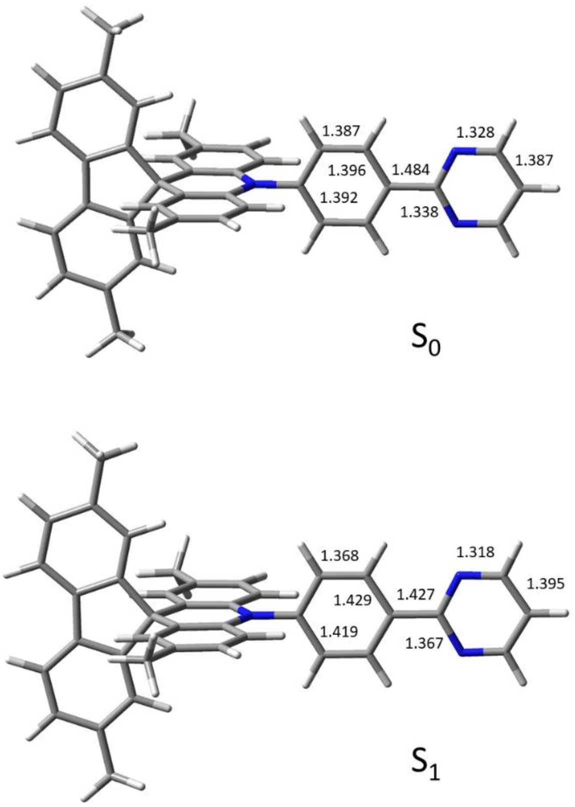

Figure S37. Significant bond length changes in Å between S_0 and S_1 optimized geometries for **1a** at CAM-B3LYP/6-31G(d). The DA N-C bond lengths are 1.429 and 1.434 Å in S_0 and S_1 respectively.

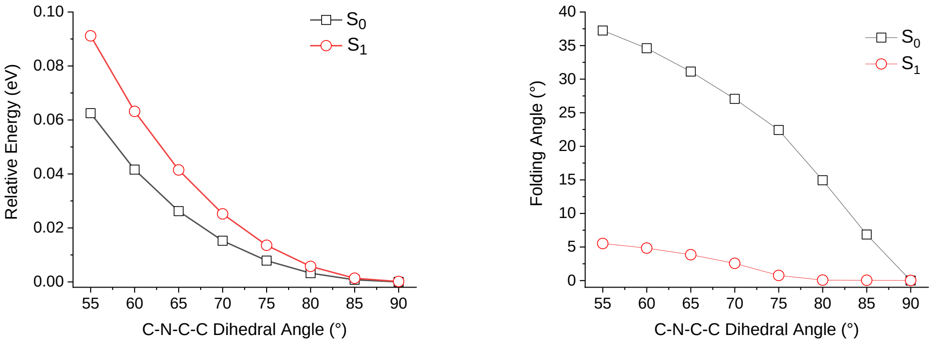

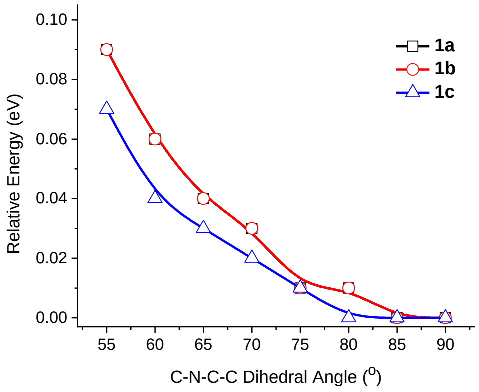

Figure S38. Plots of relative energy and acridine folding angle against the fixed C-N-C-C dihedral angle (τ) related to D-A bond rotation for **1a** in the S_0 ground and S_1 excited states at CAM-B3LYP/6-31G(d) (upper), and comparison of energies as the D-A bond is rotated for **1a**, **1b**, and **1c** in the S_1 state (lower, with energies for **1a** and **1b** coinciding).

Table S2. Experimental values of relative polarities and dielectric constants of solvents and computed excited state energies (S_1 and T_1) from TD-DFT on optimized S_1 geometries of **1a-c** using the cLR-PCM method with the corresponding solvent.

Solvent	Relative polarity index	Dielectric constant ε	S_1 eV	1a T_1 eV	S_1-T_1 eV	S_1 eV	1b T_1 eV	S_1-T_1 eV	S_1 eV	1c T_1 eV	S_1-T_1 eV
**Argon**	0.0	1.43	2.96	2.59	0.37	2.96	2.59	0.37	3.10	2.63	0.47
**Toluene**	2.4	2.57	2.82	2.59	0.23	2.82	2.59	0.23	2.99	2.63	0.36
**DCM**	3.1	8.93	2.61	2.59	0.02	2.61	2.59	0.02	2.80	2.63	0.17
**o-DCB**	2.7	9.99	2.60	2.59	0.01	2.61	2.59	0.02	2.79	2.63	0.17
**Acetonitrile**	5.8	36.71	2.53	2.53	0.00	2.53	2.53	0.00	2.71	2.63	0.08

Table S3. Energies in eV and nature of the excited states from TD-DFT computations on optimized S_1 geometries of **1a-c** in DCM. %CT values are listed in parentheses. ΔE_{ST} = S_1 energy – T_1 energy and ΔE_{TT} = T_2 energy – T_1 energy.

	T_1 ^{3}A	T_2 ^{3}CT	S_1 ^{1}CT	T_3 ^{3}Fl	ΔE_{ST}	ΔE_{TT}
**1a**	2.59 (8)	2.60 (96)	2.61 (96)	2.99 (0)	0.02	0.01
**1b**	2.59 (8)	2.60 (96)	2.61 (96)	2.99 (0)	0.02	0.01
**1c**	2.63 (1)	2.79 (95)	2.80 (95)	3.00 (0)	0.17	0.16

Table S4. Calculated spin orbit coupling matrix elements (SOCME), $<S_1|\hat{H}_{so}|T_1>$ and $<S_1|\hat{H}_{so}|T_2>$ in cm^{-1} for optimized S_1 geometries of **1a-c**.

	$<S_1\|\hat{H}_{s_o}\|T_1>$ cm^{-1}	Nature of T_1	%CT of T_1	$<S_1\|\hat{H}_{s_o}\|T_2>$ cm^{-1}	Nature of T_2	%CT of T_2
**1a**	0.68	^{3}A	8	0.00	^{3}CT	96
**1b**	0.70	^{3}A	8	0.00	^{3}CT	96
**1c**	0.66	^{3}A	1	0.00	^{3}CT	95

Cartesian coordinates of 1a-1c at S_0 ground states with total energies

1a

Total electronic energy (CAM-B3LYP/6-31G(d)) = -1668.18612 a.u.

Gibbs free energy (CAM-B3LYP/6-31G(d)) = -1667.66005 a.u.

Number of imaginary frequencies = 0

73

N -0.827276 0.000019 -0.000434
C -0.138335 -1.221791 0.017738
C -0.138368 1.221822 -0.020026
C -0.848452 -2.433664 0.035889
C 1.259098 -1.262553 0.018169
C 1.259068 1.262615 -0.020688
C -0.848512 2.433665 -0.039291
C -0.185864 -3.645373 0.053891
H -1.930831 -2.420358 0.035768
C 1.896257 -2.505121 0.036656
C 1.896190 2.505182 -0.040618
H -1.930890 2.420339 -0.038932
C -0.185958 3.645369 -0.058658
C 1.209063 -3.708729 0.054696
H -0.768015 -4.563381 0.067648
H 2.982732 -2.519999 0.036900
C 1.208966 3.708756 -0.059839
H 2.982664 2.520086 -0.040976
H -0.768133 4.563351 -0.073217
C 2.116207 0.000059 -0.000103
C 3.093399 0.018253 1.181187
C 3.096840 -0.017965 -1.178473
C 2.799218 0.035103 2.531205
C 4.421074 0.011528 0.737669
C 2.806462 -0.034947 -2.529407
C 4.423120 -0.011365 -0.731188
C 3.835176 0.048679 3.470455
H 1.764670 0.036517 2.865872
C 5.460307 0.021564 1.658472
C 3.845003 -0.048611 -3.465635
H 1.772808 -0.036416 -2.866837
C 5.465064 -0.021857 -1.649068
C 5.156215 0.039158 3.016124
H 6.496208 0.012245 1.331465
C 5.164859 -0.039509 -3.007488
H 6.499982 -0.012982 -1.318960
H 5.963757 0.044258 3.743318
H 5.974361 -0.045116 -3.732476
C -2.256401 -0.000006 -0.000512
C -2.956422 -0.019010 -1.204448
C -2.956459 0.018970 1.203386
C -4.343489 -0.019069 -1.205268
H -2.401775 -0.033730 -2.136968
C -4.343535 0.018987 1.204161
H -2.401835 0.033709 2.135918
C -5.049327 -0.000050 -0.000562
H -4.897362 -0.033775 -2.136163
H -4.897437 0.033677 2.135038
C -6.533499 -0.000069 -0.000590
N -7.150854 -0.018926 -1.187485
N -7.150899 0.018772 1.186283
C -8.479278 -0.018776 -1.176563
C -8.479322 0.018591 1.175311
C -9.215400 -0.000102 -0.000640
H -8.970093 -0.034208 -2.147535
H -8.970174 0.034011 2.146264
H -10.298783 -0.000115 -0.000660
C 3.525906 0.092203 4.944897
H 3.213311 1.095940 5.255749
H 2.712436 -0.593195 5.202307
H 4.398689 -0.180396 5.544516
C 3.540060 -0.091769 -4.940990
H 4.416301 0.175060 -5.538105
H 3.222378 -1.093926 -5.251777
H 2.731470 0.598176 -5.201735
C 1.930661 5.030796 -0.081457
H 1.676491 5.613051 -0.974272
H 1.670777 5.644996 0.787979
H 3.014750 4.889782 -0.075493
C 1.930792 -5.030778 0.074509
H 3.014883 -4.889682 0.071728
H 1.674319 -5.615539 0.965012
H 1.673235 -5.642568 -0.797330

1b

Total electronic energy (CAM-B3LYP/6-31G(d)) = -2368.20301 a.u.

Gibbs free energy (CAM-B3LYP/6-31G(d)) = -2367.29086 a.u.

Number of imaginary frequencies = 0

115

N 0.171714 1.858194 0.000050
C 0.096709 1.173275 -1.221960
C 0.096774 1.173409 1.222120
C 0.162253 1.880431 -2.433942
C -0.044151 -0.217065 -1.262511
C -0.044000 -0.216951 1.262768
C 0.162379 1.880634 2.434049
C 0.089598 1.221810 -3.645786
H 0.271224 2.957320 -2.420640
C -0.114643 -0.850262 -2.505228
C -0.114419 -0.850059 2.505546
H 0.271309 2.957525 2.420699
C 0.089820 1.222083 3.645946
C -0.051404 -0.165985 -3.708991
H 0.143719 1.801430 -4.563918
H -0.223811 -1.931232 -2.519759
C -0.051123 -0.165697 3.709270
H -0.223550 -1.931028 2.520158
H 0.143958 1.801780 4.564029
C -0.118839 -1.070997 0.000197
C -1.378351 -1.945367 0.000206
C 0.972577 -2.148336 0.000156
C -2.700260 -1.531655 0.000178
C -1.048150 -3.301325 0.000198
C 2.345785 -1.967223 0.000103
C 0.414886 -3.427636 0.000165
C -3.732945 -2.473834 0.000166
H -2.918572 -0.470080 0.000160
C -2.059572 -4.255197 0.000248
C 3.201741 -3.072437 0.000008
H 2.742884 -0.958774 0.000136
C 1.247860 -4.540693 0.000067
C -3.381726 -3.832791 0.000235
H -1.826891 -5.316386 0.000288
C 2.622855 -4.351096 -0.000012
H 0.836819 -5.546337 0.000027
H -4.164291 -4.585119 0.000224
H 3.264886 -5.226411 -0.000076
C -0.130209 -0.883145 5.031462
H 0.769300 -0.715521 5.634397
H -0.984293 -0.538835 5.625396
H -0.238172 -1.961847 4.890282
C -0.130626 -0.883509 -5.031134
H -0.984713 -0.539163 -5.625041
H 0.768862 -0.715999 -5.634137
H -0.238672 -1.962193 -4.889884
C -5.209070 -2.077072 0.000038
C -5.914317 -2.646137 1.255987
C -5.914130 -2.646352 -1.255924
C -5.414910 -0.548168 -0.000132
C -7.409396 -2.296065 1.254593
H -5.430301 -2.244002 2.154780
H -5.791353 -3.734551 1.293812
C -7.409219 -2.296290 -1.254814
H -5.429977 -2.244361 -2.154709
H -5.791142 -3.734773 -1.293546
C -6.908349 -0.189502 -0.000267
H -4.933344 -0.107889 -0.882026
H -4.933410 -0.107669 0.881700
C -7.576932 -0.770820 1.252820
C -8.071272 -2.884588 -0.000103
H -7.878304 -2.718590 2.151423
C -7.576764 -0.771042 -1.253337
H -7.877986 -2.718992 -2.151634
H -7.011770 0.902163 -0.000367
H -8.641846 -0.506222 1.270037
H -7.124861 -0.341033 2.155659
H -7.973428 -3.977814 0.000007
H -9.145422 -2.659863 -0.000197
H -8.641674 -0.506446 -1.270734
H -7.124576 -0.341414 -2.156194
C 4.723999 -2.934468 -0.000059
C 5.321262 -3.615940 -1.256044
C 5.321372 -3.616066 1.255794
C 5.188626 -1.463463 -0.000007
C 6.854232 -3.527090 -1.254874
H 4.913196 -3.136901 -2.154819
H 5.013682 -4.667206 -1.293807
C 6.854346 -3.527216 1.254498
H 4.913392 -3.137125 2.154661
H 5.013792 -4.667338 1.293475
C 6.721501 -1.365832 -0.000070
H 4.789688 -0.947195 0.881935
H 4.789605 -0.947097 -0.881854
C 7.280475 -2.053077 -1.253250
C 7.405815 -4.220143 -0.000248
H 7.243701 -4.023706 -2.151751
C 7.280589 -2.053203 1.252989
H 7.243885 -4.023932 2.151289
H 7.010337 -0.308001 -0.000035
H 8.374961 -1.974713 -1.270683
H 6.908479 -1.552272 -2.156039
H 7.122318 -5.280494 -0.000287
H 8.502576 -4.182558 -0.000295
H 8.375076 -1.974848 1.270330
H 6.908677 -1.552493 2.155865

C	0.321492	3.279385	-0.000004
C	1.592425	3.849088	-0.000211
C	-0.802356	4.102166	0.000184
C	1.738967	5.228398	-0.000224
H	2.461657	3.199565	-0.000355
C	-0.657393	5.481637	0.000185
H	-1.788184	3.648737	0.000340
C	0.614975	6.056952	-0.000015
H	2.723029	5.681370	-0.000380
H	-1.525066	6.130252	0.000339
C	0.770943	7.532879	0.000002
N	2.016286	8.022128	-0.000275
N	-0.344629	8.271623	0.000129
C	2.144996	9.344339	-0.000362
C	-0.194131	9.591535	0.000038
C	1.052792	10.199975	-0.000200
H	3.162291	9.730398	-0.000556
H	-1.108263	10.181699	0.000172
H	1.166648	11.277359	-0.000266

1c

Total electronic energy (CAM-B3LYP/6-31G(d)) = -3146.82047 a.u.

Gibbs free energy (CAM-B3LYP/6-31G(d)) = -3145.47021 a.u.

Number of imaginary frequencies = 0

163

N	0.959720	-0.000350	-0.000079
C	1.648783	-0.000333	-1.221691
C	1.648723	-0.000057	1.221561
C	0.938623	0.000111	-2.433815
C	3.046338	-0.000302	-1.262642
C	3.046276	0.000202	1.262581
C	0.938500	-0.000584	2.433644
C	1.601127	0.000277	-3.645677
H	-0.143729	0.000734	-2.420002
C	3.683356	-0.000162	-2.505432
C	3.683229	0.000305	2.505404
H	-0.143852	-0.001530	2.419771
C	1.600941	-0.000500	3.645540
C	2.996090	-0.000182	-3.709134
H	1.018897	0.000986	-4.563780
H	4.769839	0.000105	-2.520092
C	2.995900	0.000320	3.709070
H	4.769712	0.000212	2.520117
H	1.018662	-0.001310	4.563612
C	3.903669	-0.000148	-0.000009
C	4.883020	-1.179877	0.000220
C	4.883337	1.179313	-0.000193
C	4.583766	-2.532168	0.000443
C	6.205755	-0.734785	0.000178
C	4.584444	2.531686	-0.000435
C	6.205952	0.733865	-0.000075
C	5.610566	-3.480703	0.000624
H	3.544654	-2.840046	0.000487
C	7.242443	-1.661186	0.000359
C	5.611494	3.479943	-0.000554
H	3.545413	2.839847	-0.000529
C	7.242888	1.659991	-0.000197
C	6.934526	-3.014720	0.000579
H	8.279940	-1.338860	0.000328
C	6.935333	3.013606	-0.000429
H	8.280299	1.337389	-0.000110
H	7.750992	-3.730148	0.000722
H	7.751993	3.728812	-0.000519
C	3.717550	0.003688	5.031350
H	3.436171	-0.859114	5.645321
H	4.801232	-0.028562	4.890575
H	3.485497	0.902401	5.614013
C	3.717804	-0.002854	-5.031381
H	4.801539	0.027087	-4.890496
H	3.484013	-0.900140	-5.615523
H	3.438159	0.861515	-5.643960
C	5.340251	-4.985169	0.000863
C	5.966767	-5.639815	1.256814
C	5.966733	-5.640201	-1.254904
C	3.834196	-5.318755	0.000936
C	5.743440	-7.159102	1.255876
H	5.525646	-5.191056	2.155602
H	7.041031	-5.425860	1.294287
C	5.743404	-7.159485	-1.253495
H	5.525588	-5.191713	-2.153817
H	7.040996	-5.426260	-1.292474
C	3.602109	-6.837102	0.001173
H	3.354910	-4.876087	-0.880985
H	3.354928	-4.875812	0.882730
C	4.237676	-7.454142	1.254351
C	6.385313	-7.769556	0.001275
H	6.203954	-7.590609	2.152782

C	4.237640	-7.454526	-1.251835
H	6.203890	-7.591270	-2.150282
H	2.522974	-7.031804	0.001217
H	4.063508	-8.537529	1.271941
H	3.771563	-7.039462	2.157118
H	7.466458	-7.580192	0.001232
H	6.251595	-8.858796	0.001442
H	4.063471	-8.537919	-1.269091
H	3.771499	-7.040122	-2.154715
C	5.341541	4.984472	-0.000804
C	5.968230	5.638991	-1.256739
C	5.968105	5.639380	1.254990
C	3.835555	5.318352	-0.000931
C	5.745204	7.158321	-1.255806
H	5.527046	5.190318	-2.155539
H	7.042452	5.424825	-1.294179
C	5.745077	7.158709	1.253568
H	5.526834	5.190984	2.153886
H	7.042322	5.425229	1.292608
C	3.603766	6.836745	-0.001177
H	3.356147	4.875769	0.880970
H	3.356239	4.875500	-0.882744
C	4.239498	7.453659	-1.254334
C	6.387152	7.768649	-0.001181
H	6.205832	7.589741	-2.152697
C	4.239371	7.454047	1.251852
H	6.205616	7.590405	2.150372
H	2.524669	7.031656	-0.001263
H	4.065542	8.537080	-1.271931
H	3.773334	7.039069	-2.157116
H	7.468260	7.579071	-0.001095
H	6.253651	8.857916	-0.001356
H	4.065413	8.537474	1.269096
H	3.773117	7.039739	2.154717
C	-0.469812	-0.000228	-0.000093
C	-1.170324	1.203314	0.000643
C	-1.170539	-1.203644	-0.000821
C	-2.557586	1.203502	0.000641
H	-0.615955	2.136239	0.001207
C	-2.557803	-1.203584	-0.000821
H	-0.616335	-2.136668	-0.001389
C	-3.265373	0.000023	-0.000090
H	-3.110909	2.134651	0.001191
H	-3.111290	-2.134634	-0.001370
C	-4.752951	0.000168	-0.000074
N	-5.365687	1.184069	0.000347
N	-5.365939	-1.183607	-0.000481
C	-6.701116	1.195102	0.000371
C	-6.701369	-1.194357	-0.000451
C	-7.417051	0.000450	-0.000008
C	-7.352146	2.568574	0.000843
C	-7.352691	-2.567692	-0.000850
H	-8.496424	0.000566	0.000051
C	-6.891463	3.347431	-1.254371
C	-8.890956	2.511262	0.000927

C	-6.891294	3.346689	1.256449
C	-6.892028	-3.346660	1.254295
C	-8.891490	-2.510063	-0.000763
C	-6.892138	-3.345888	-1.256521
H	-7.210169	2.808127	-2.155946
H	-5.797858	3.381903	-1.271415
C	-7.479487	4.764954	-1.252874
H	-9.247940	1.965402	0.883726
H	-9.248058	1.965939	-0.882156
C	-9.481863	3.929905	0.001390
H	-5.797688	3.381152	1.273369
H	-7.209878	2.806857	2.157752
C	-7.479316	4.764214	1.255869
H	-7.210518	-2.807305	2.155916
H	-5.798429	-3.381360	1.271218
C	-7.480346	-4.764062	1.252849
H	-9.248464	-1.964131	-0.883522
H	-9.248380	-1.964667	0.882361
C	-9.482692	-3.928582	-0.001172
H	-5.798540	-3.380580	-1.273559
H	-7.210709	-2.805974	-2.157778
C	-7.480459	-4.763290	-1.255892
H	-7.138447	5.295307	-2.149829
C	-9.012372	4.682395	-1.251843
C	-7.006369	5.513742	0.001686
H	-10.575950	3.859684	0.001444
C	-9.012201	4.681654	1.255002
H	-7.138152	5.294036	2.153090
H	-7.139311	-5.294495	2.149758
C	-9.013215	-4.681185	1.251997
C	-7.007528	-5.512934	-0.001773
H	-10.576765	-3.858134	-0.001102
C	-9.013327	-4.680410	-1.254848
H	-7.139509	-5.293174	-2.153158
H	-9.363044	4.167226	-2.155200
H	-9.446119	5.690054	-1.269237
H	-5.912241	5.594106	0.001635
H	-7.403478	6.536649	0.002015
H	-9.445948	5.689302	1.273053
H	-9.362745	4.165950	2.158104
H	-9.363673	-4.165955	2.155402
H	-9.447170	-5.688754	1.269428
H	-5.913417	-5.593528	-0.001847
H	-7.404852	-6.535758	-0.002070
H	-9.447285	-5.687968	-1.272865
H	-9.363868	-4.164620	-2.157903

6. Photophysical properties

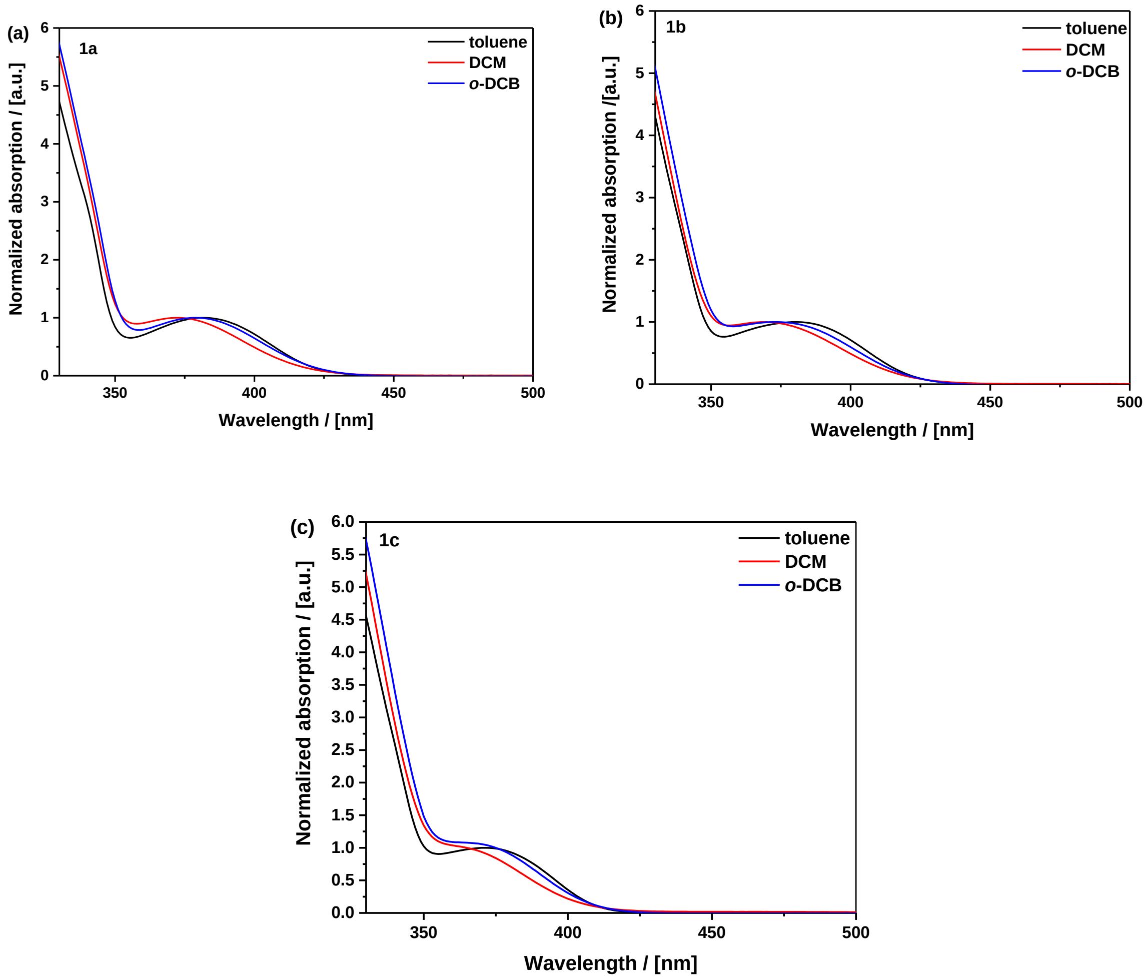

Figure S39. Absorption spectra of 5% (w/w) solutions of (a) **1a**, (b) **1b** and (c) **1c** in toluene, DCM and *o*-DCB.

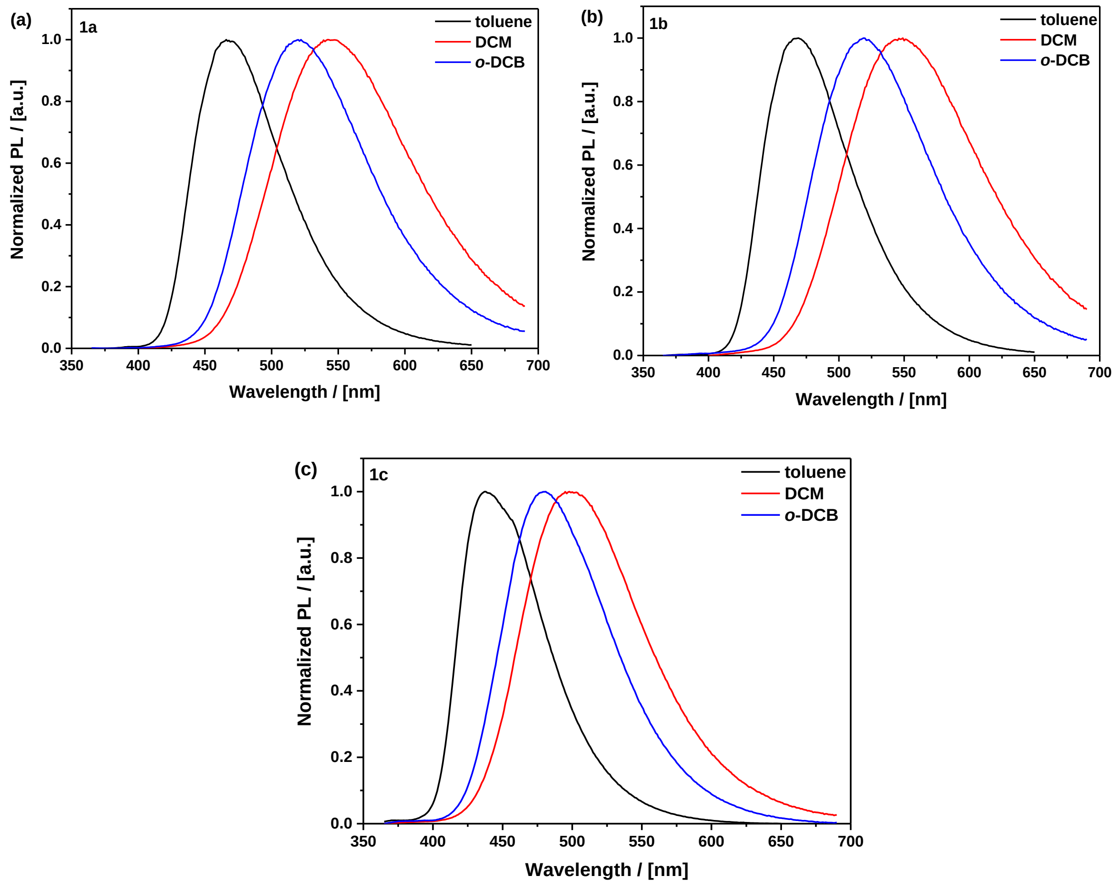

Figure S40. Photoluminescence spectra of 5% (w/w) solutions of (a) **1a**, (b) **1b** and (c) **1c** in toluene, DCM and *o*-DCB.

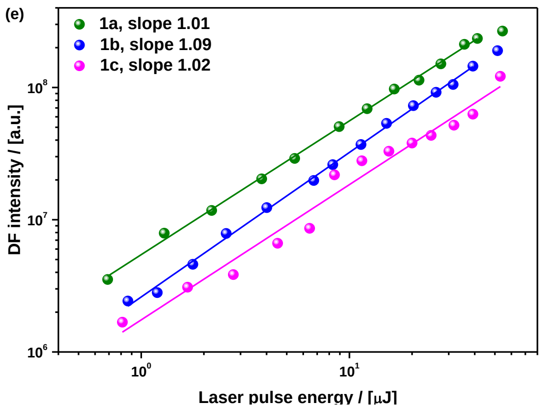

Figure S41. DF intensity dependence on the laser pulse energy (delay time / integration time: **1a** (1 μs / 400 ns), **1b** (2 μs / 800 ns), **1c** (3 μs / 20 μs) of 5% (w/w) Zeonex films of **1a**, **1b** and **1c**, recorded at room temperature.

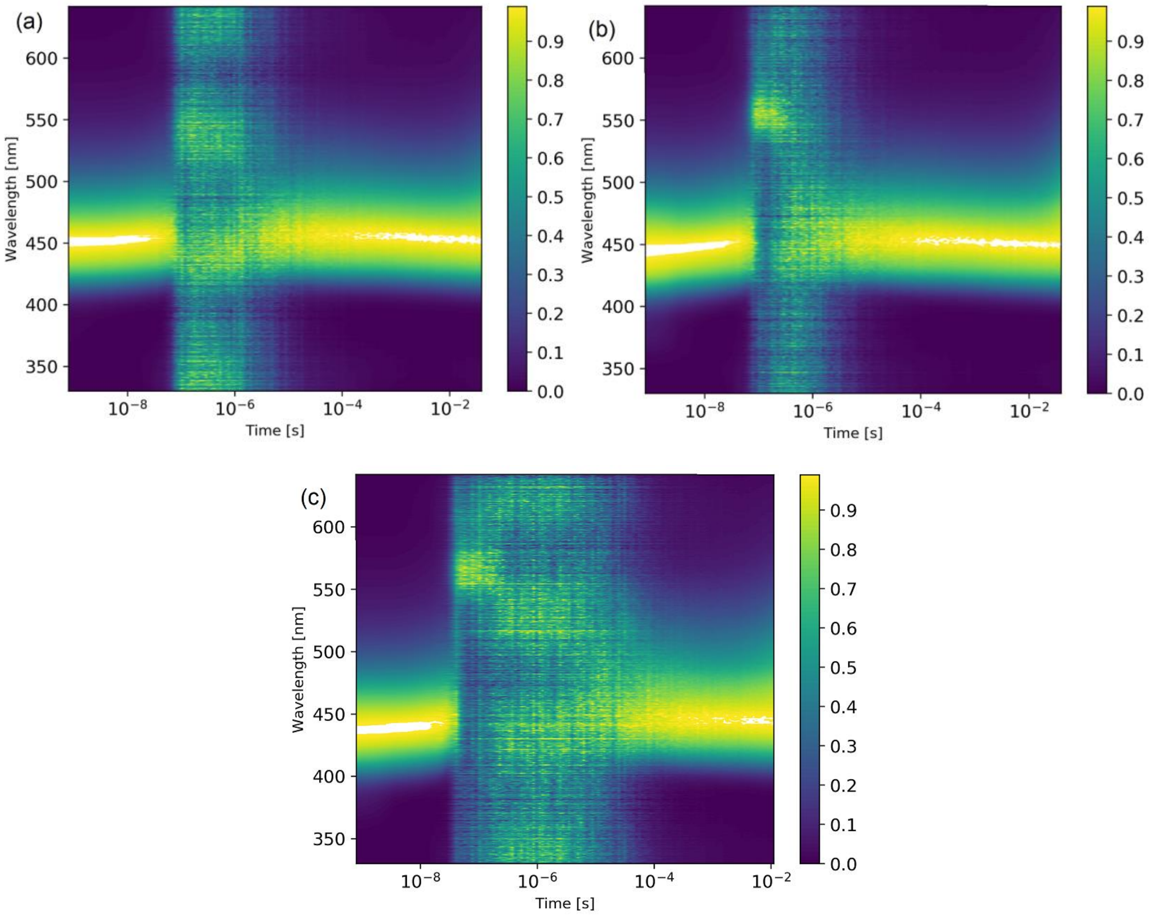

Figure S42. Maps of normalized emission spectra of 5% Zeonex films of (a) **1a**, (b) **1b** and (c) **1c** (excitation 355 nm, Nd-YAG laser, room temperature).

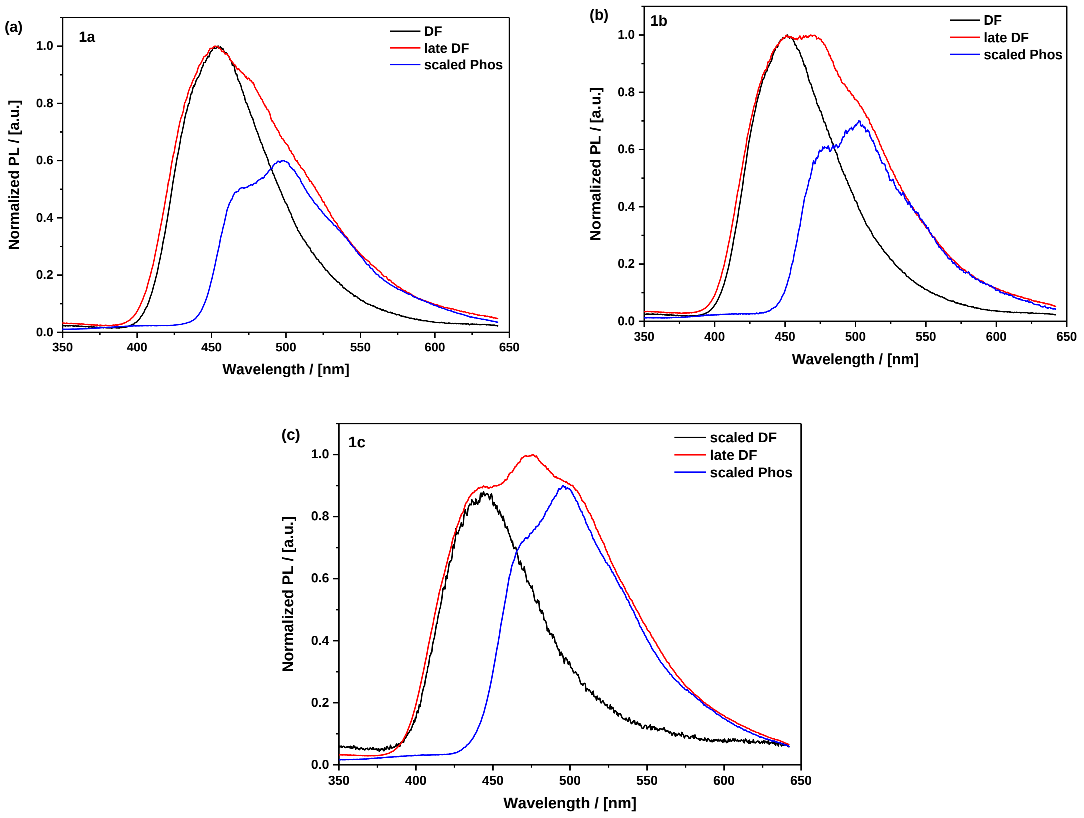

Figure S43. Comparison of the room temperature early DF (delay 4 μs), late DF (delay 40 ms) and 80 K phosphorescence (delay 10 ms) of 5% Zeonex films of (a) **1a**; (b) **1b** and (c) **1c** (excitation 355 nm, Nd-YAG laser).

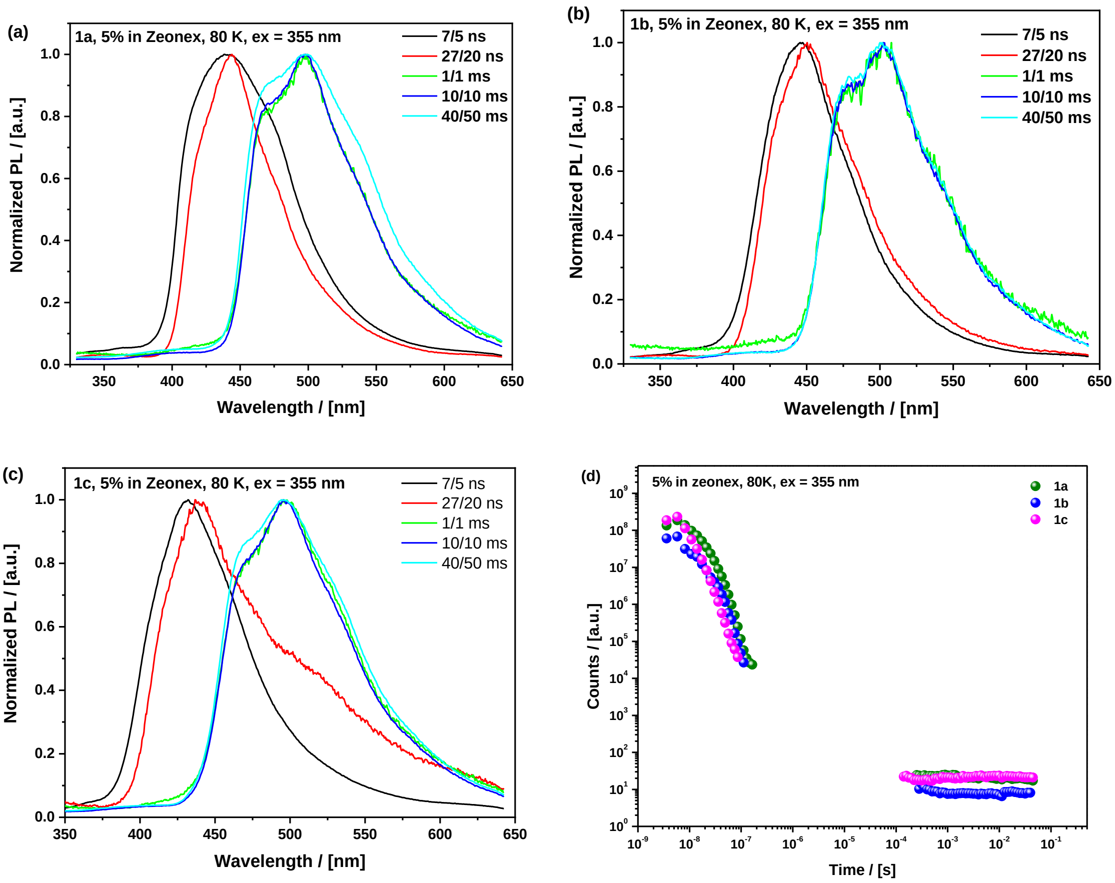

Figure S44. Time-resolved photoluminescence spectra of 5% Zeonex films of (a) **1a**; (b) **1b** and (c) **1c** recorded at 80 K (excitation 355 nm, Nd-YAG laser). (d) Decay curves of 5% Zeonex films of **1a**, **1b** and **1c** recorded at 80 K.

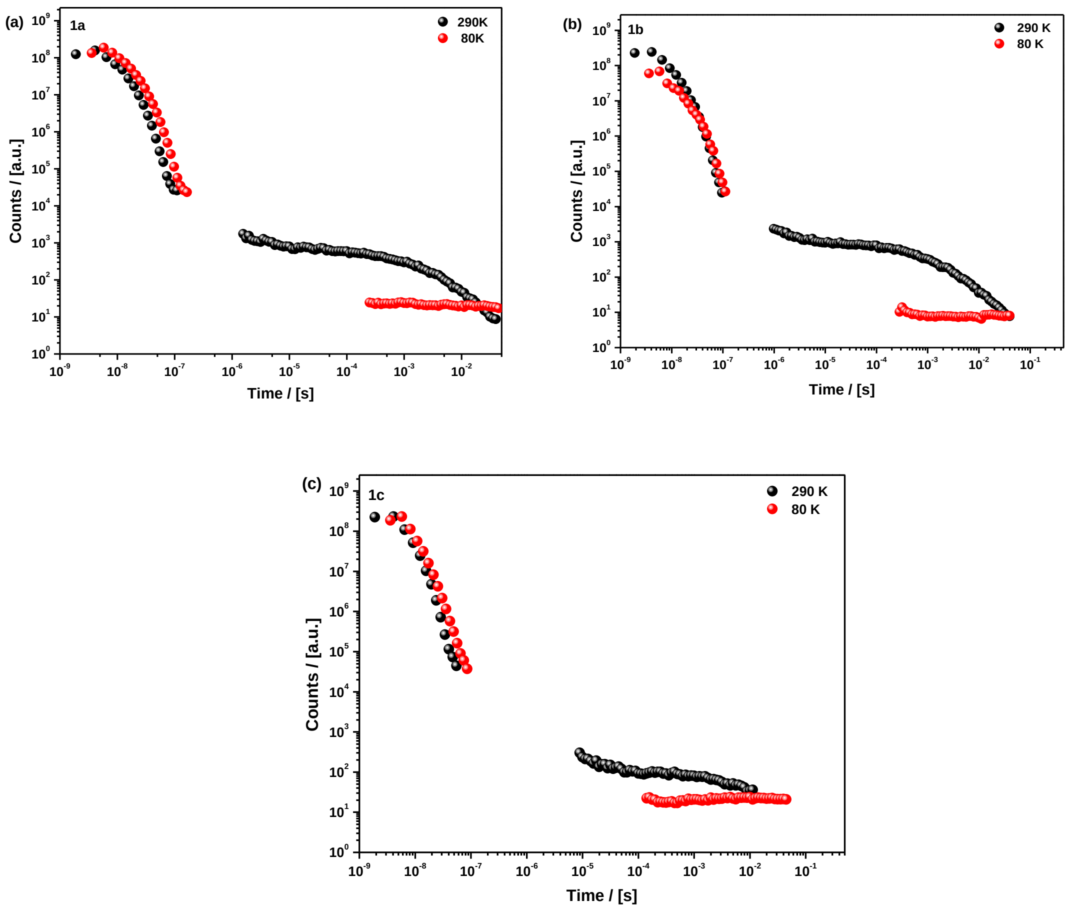

Figure S45. Decay curves of 5% Zeonex films of (a) **1a**, (b) **1b** and (c) **1c** recorded at 290 K and 80 K.

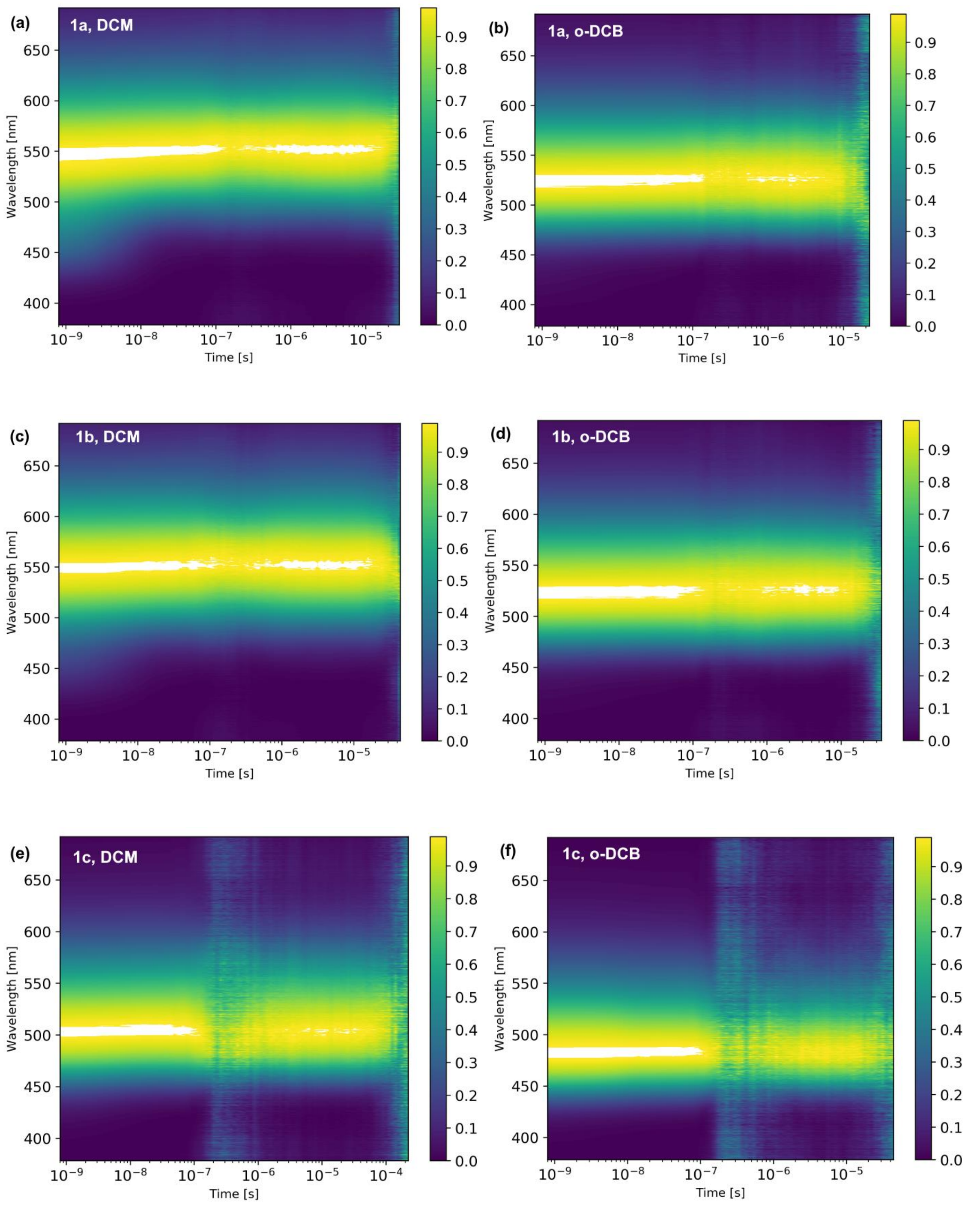

Figure S46. Maps of normalized emission spectra of (a, c, e) DCM and (b, d, f) *o*-DCB solutions of **1a**, **1b** and **1c** (excitation 355 nm, Nd-YAG laser, room temperature).

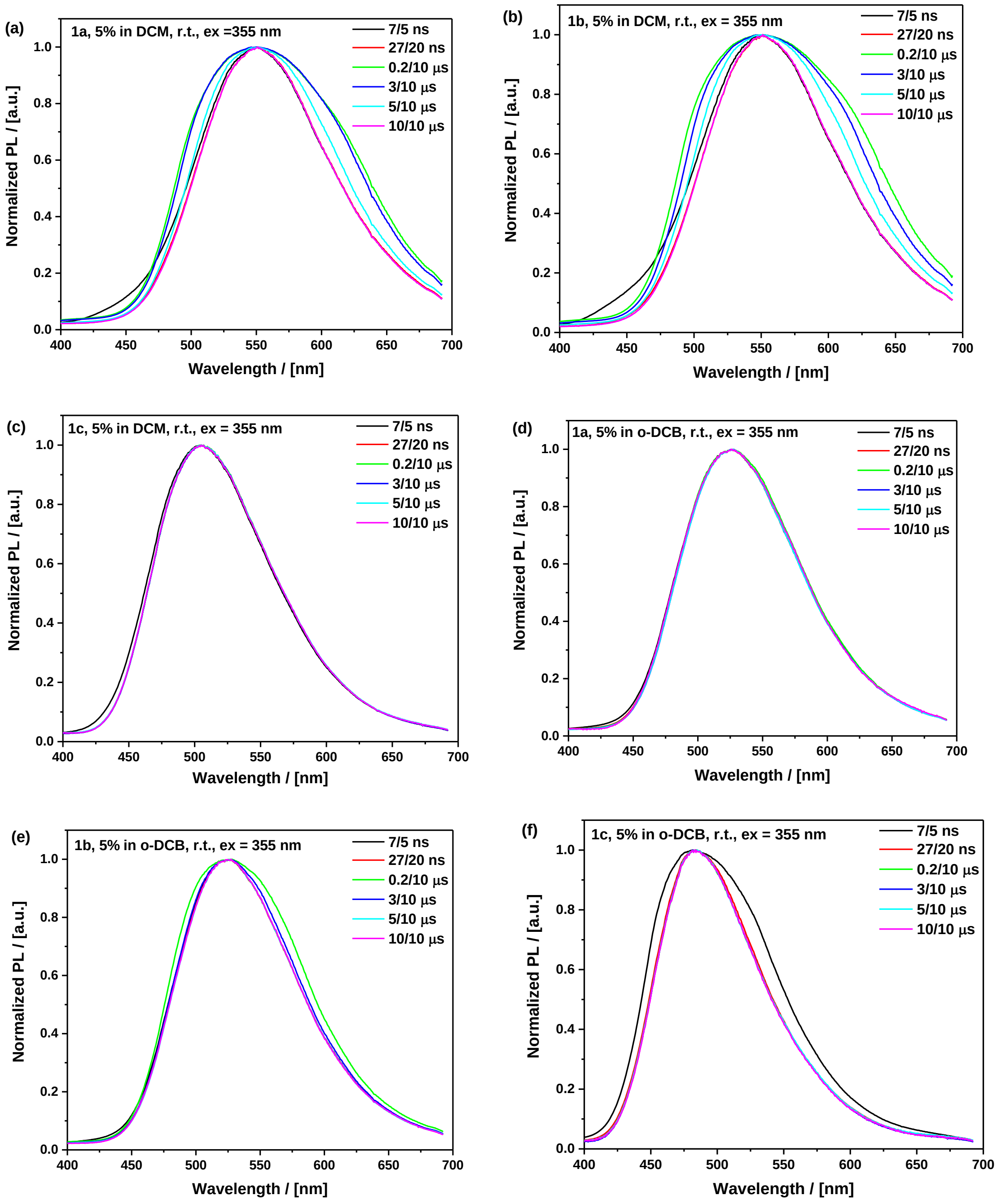

Figure S47. Time-resolved photoluminescence spectra of 5% DCM solutions of (a) **1a**; (b) **1b**; (c) **1c** and 5% *o*-DCB solutions of (d) **1a**; (e) **1b**; (f) **1c** recorded at 80 K (excitation 355 nm, Nd-YAG laser).

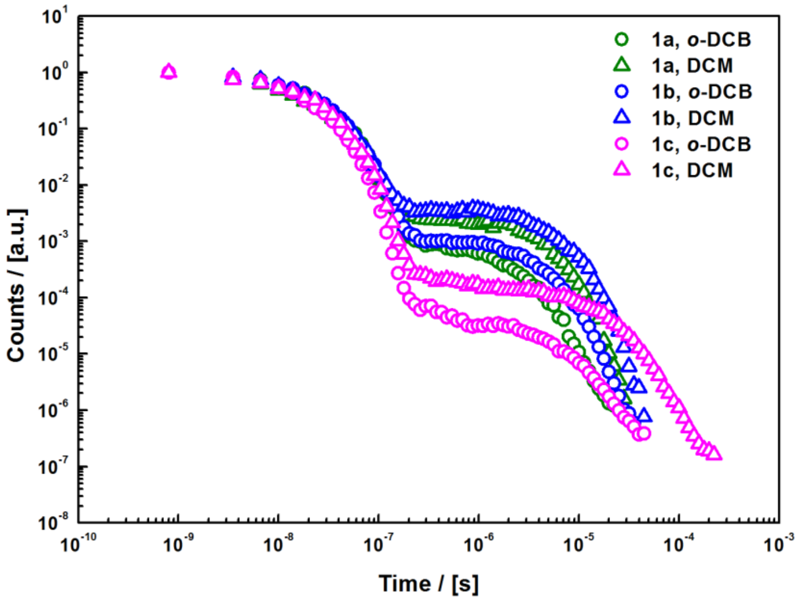

Figure S48. Decay curves of 5% solutions of **1a**, **1b** and **1c** in DCM and *o*-DCB.

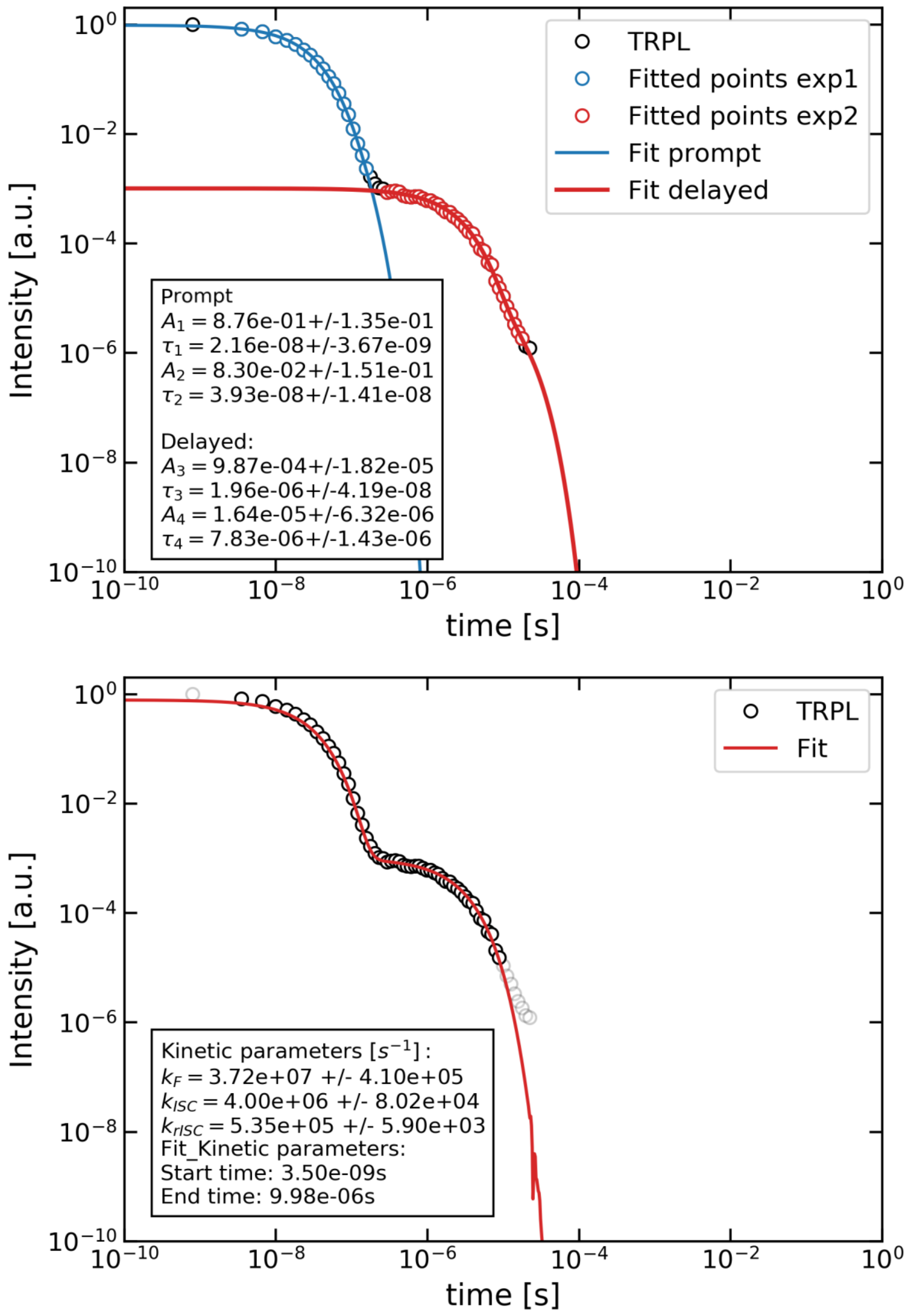

Figure S49. Emission decay fitting for **1a** in o-DCB (top biexponential, bottom kinetic)

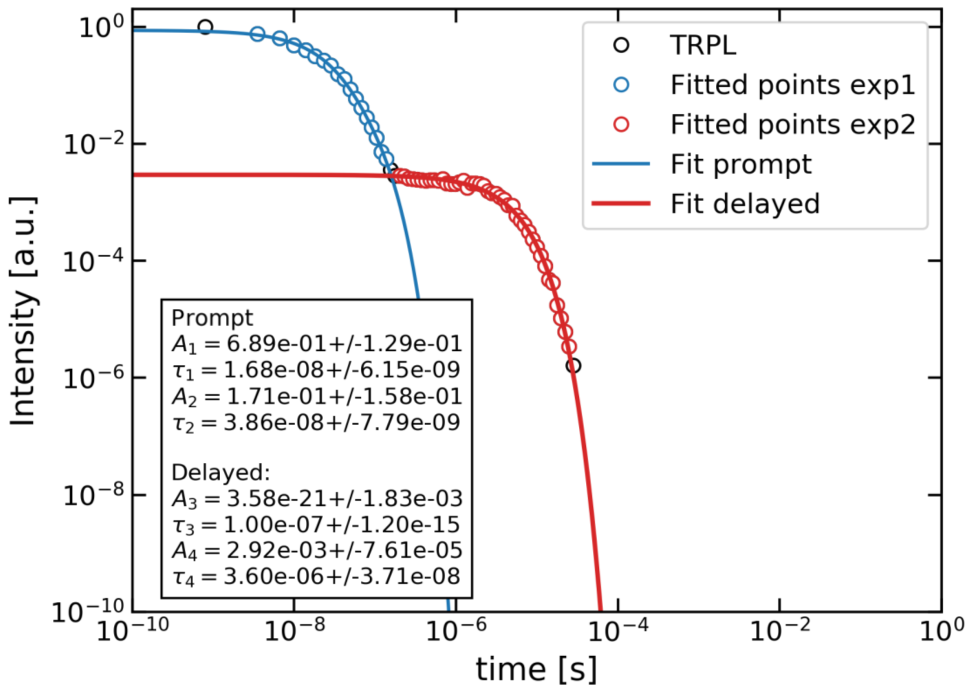

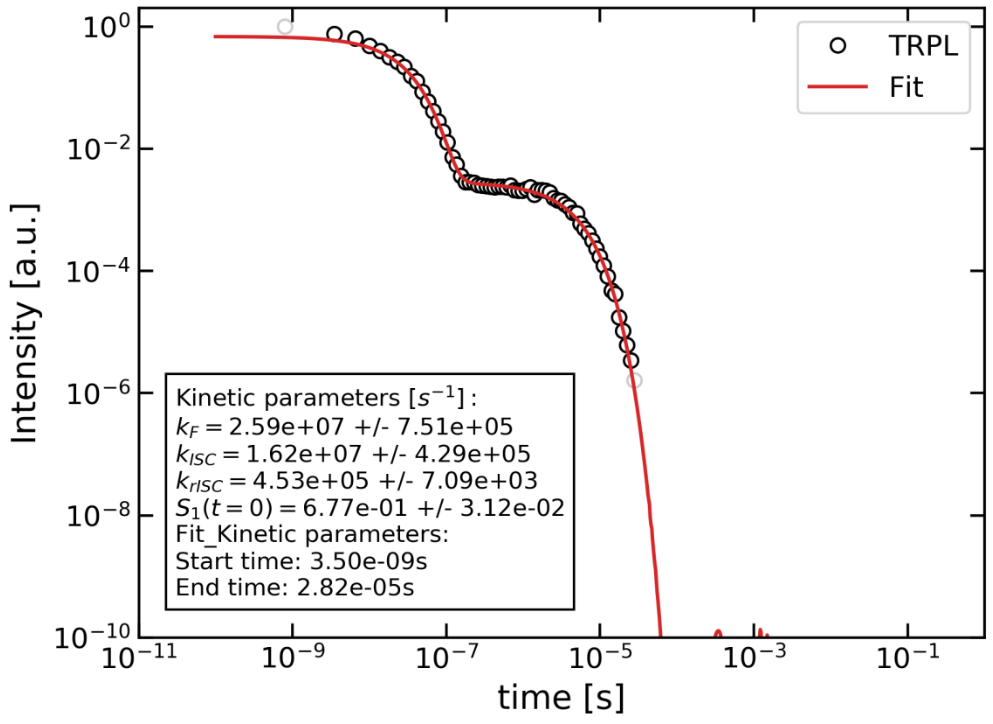

Figure S50. Emission decay fitting for **1a** in DCM (top biexponential, bottom kinetic)

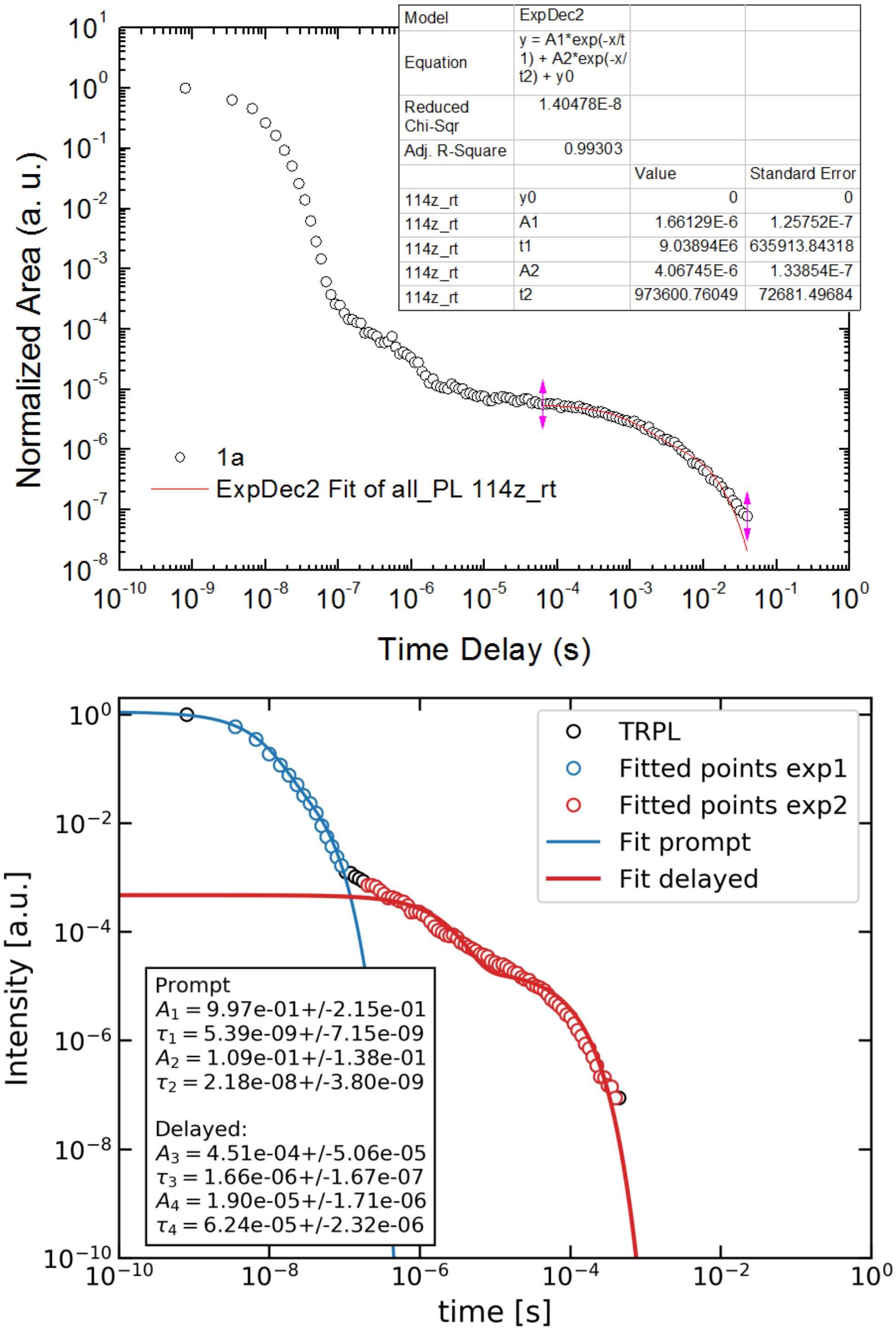

Figure S51- Emission decay fitting for **1a** in Zeonex (top) and neat film (bottom)

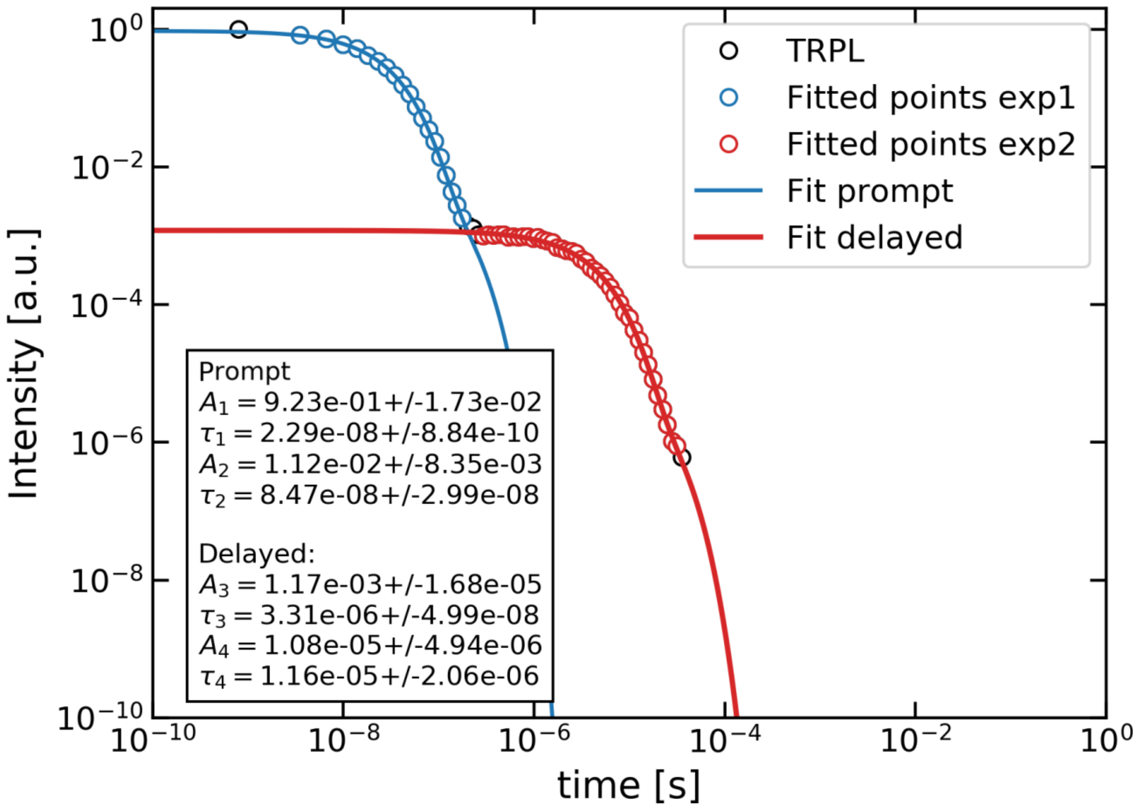

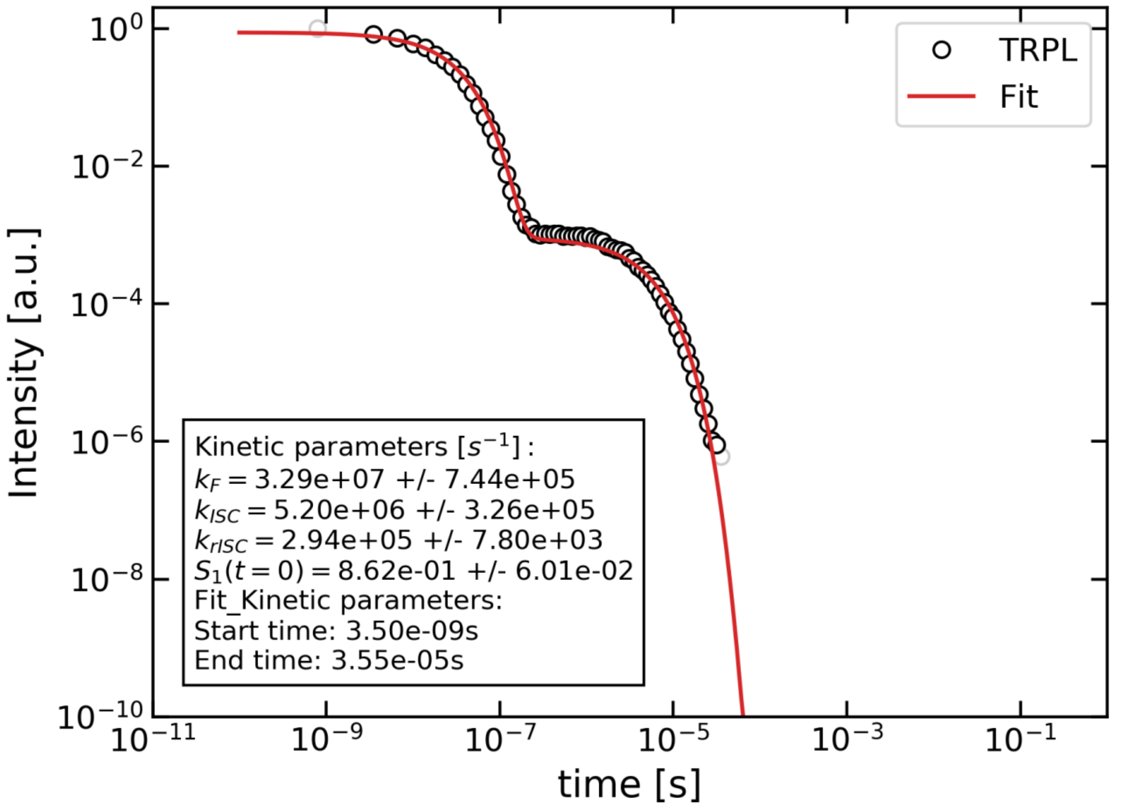

Figure S52. Emission decay fitting for **1b** in o-DCB (top biexponential, bottom kinetic)

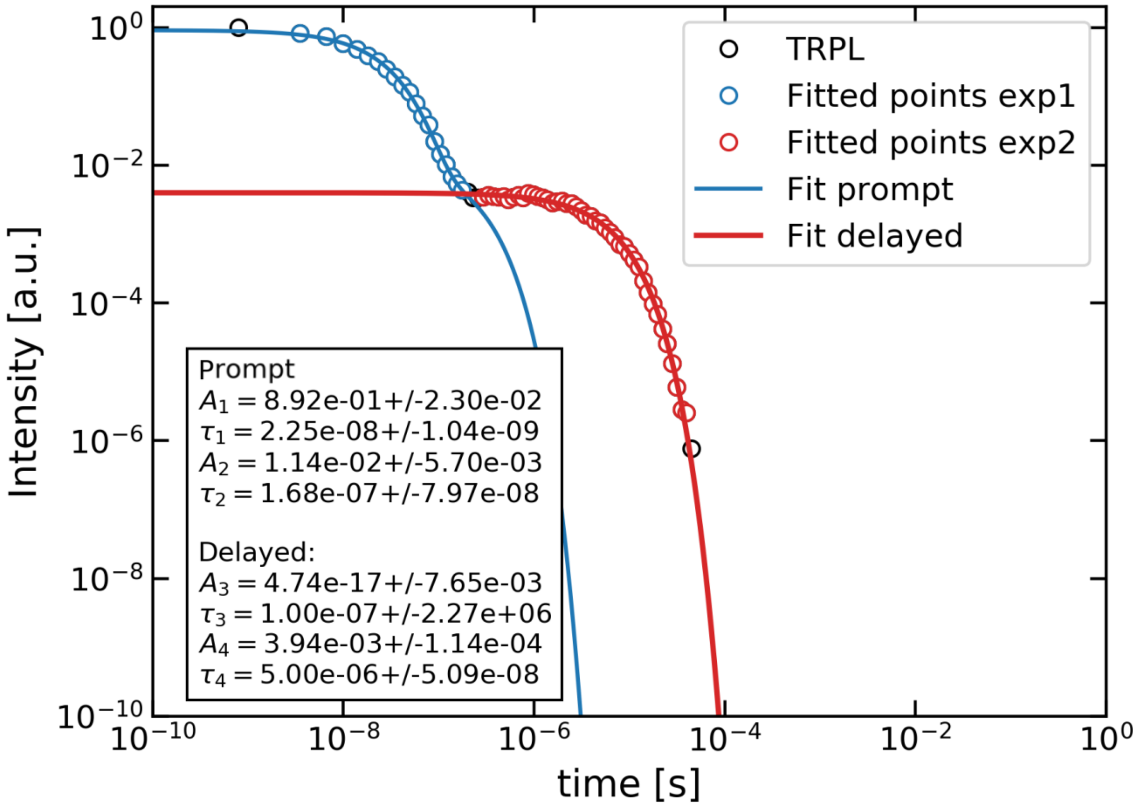

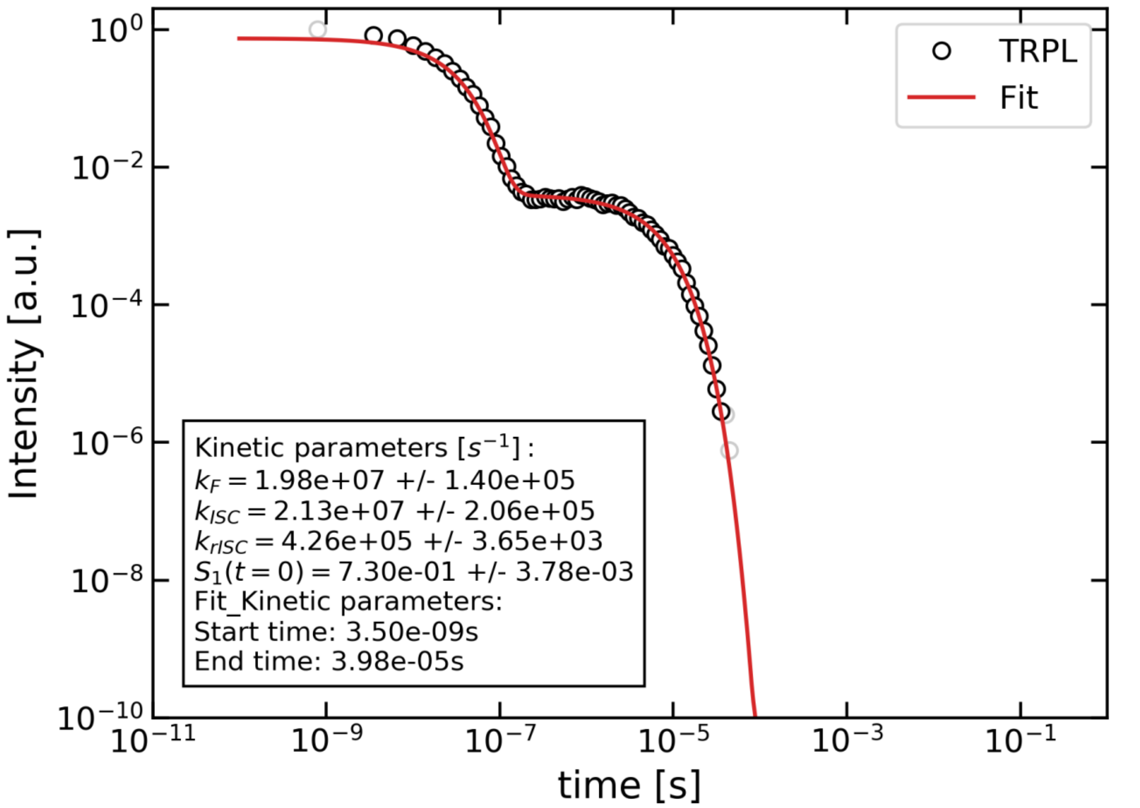

Figure S53. Emission decay fitting for **1b** in DCM (top biexponential, bottom kinetic)

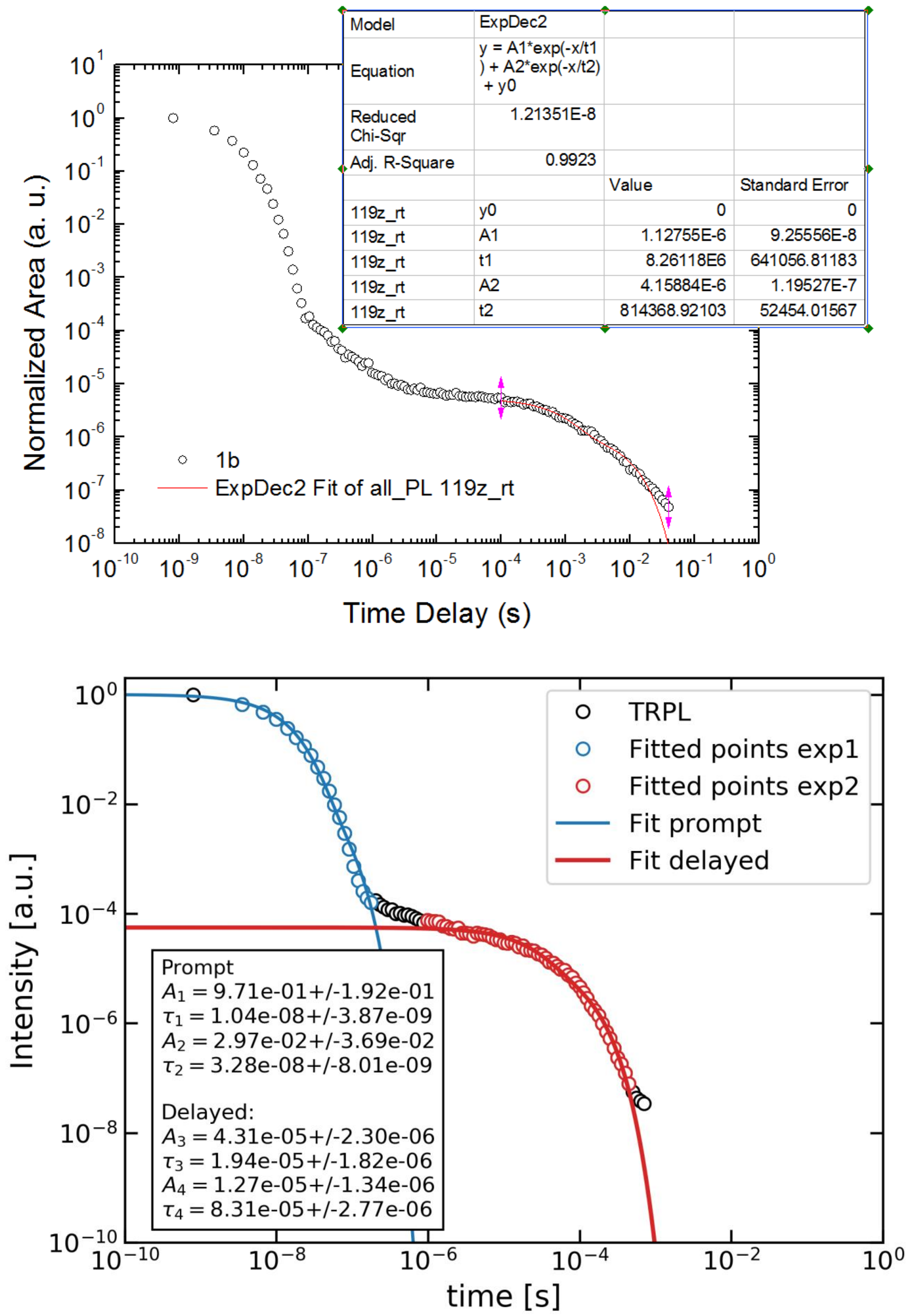

Figure S54. Emission decay fitting for **1b** in Zeonex (top) and neat film (bottom)

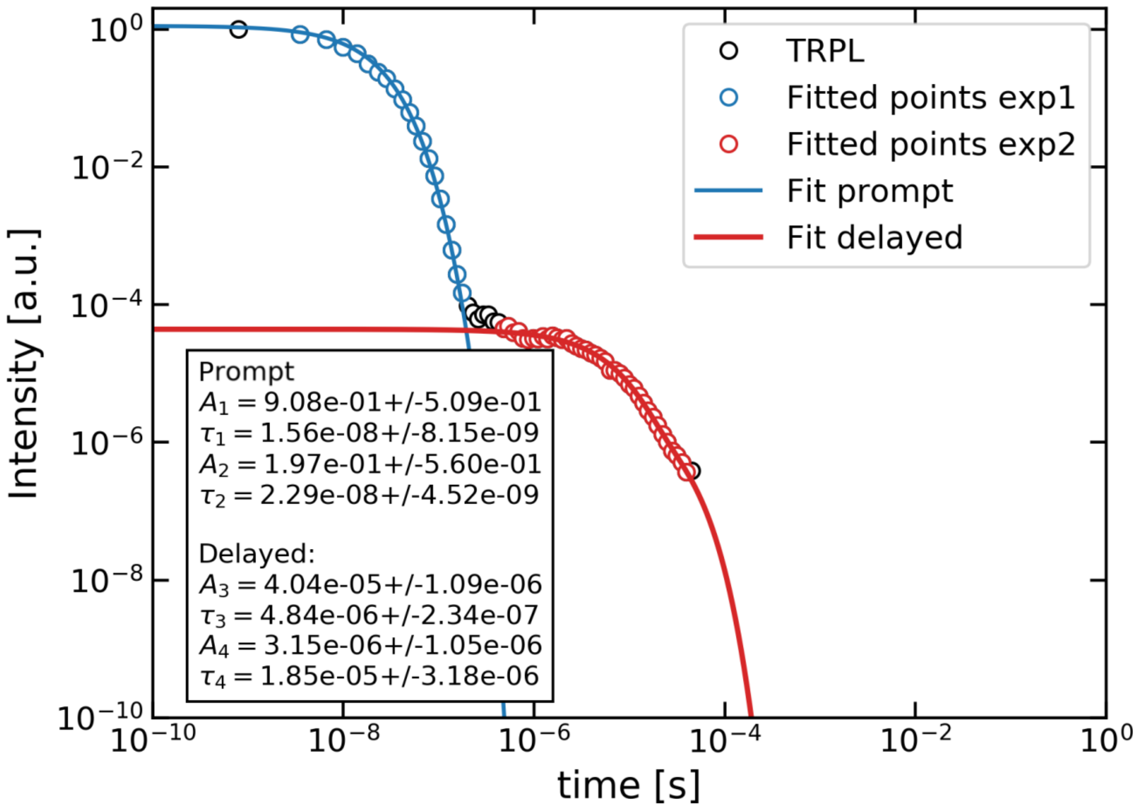

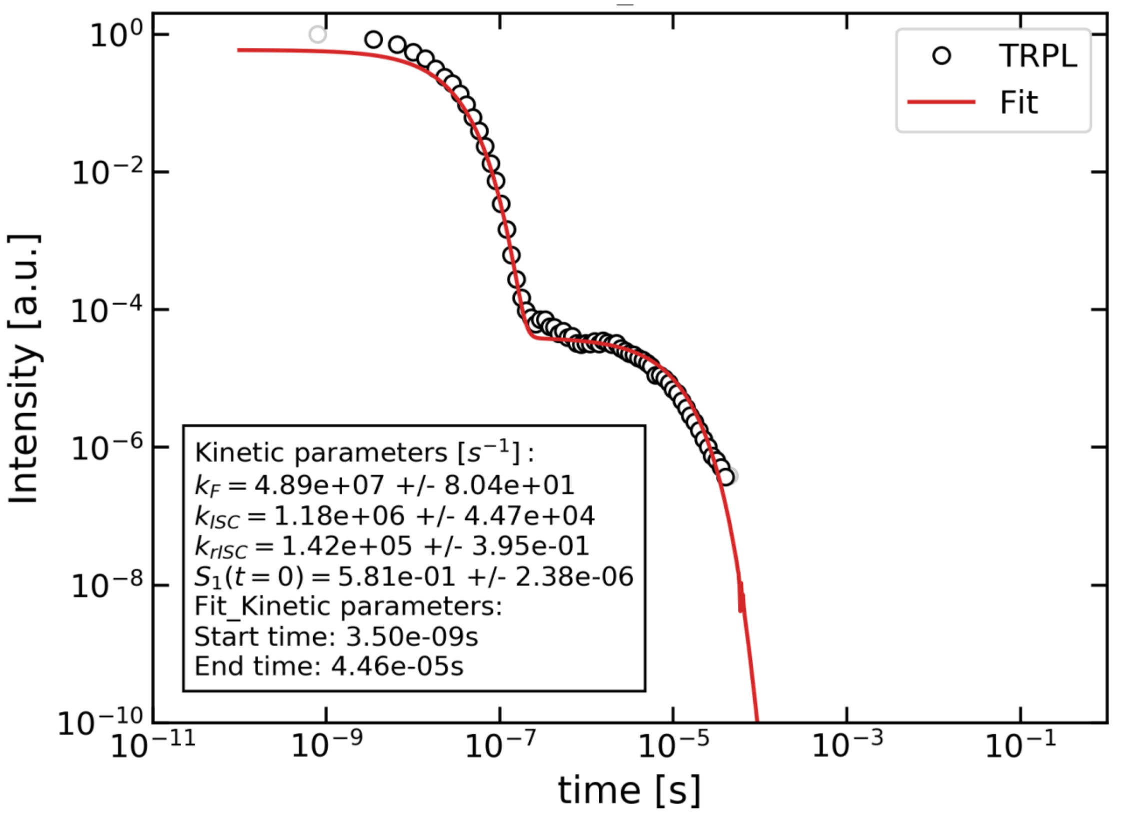

Figure S55. Emission decay fitting for **1c** in o-DCB (top biexponential, bottom kinetic)

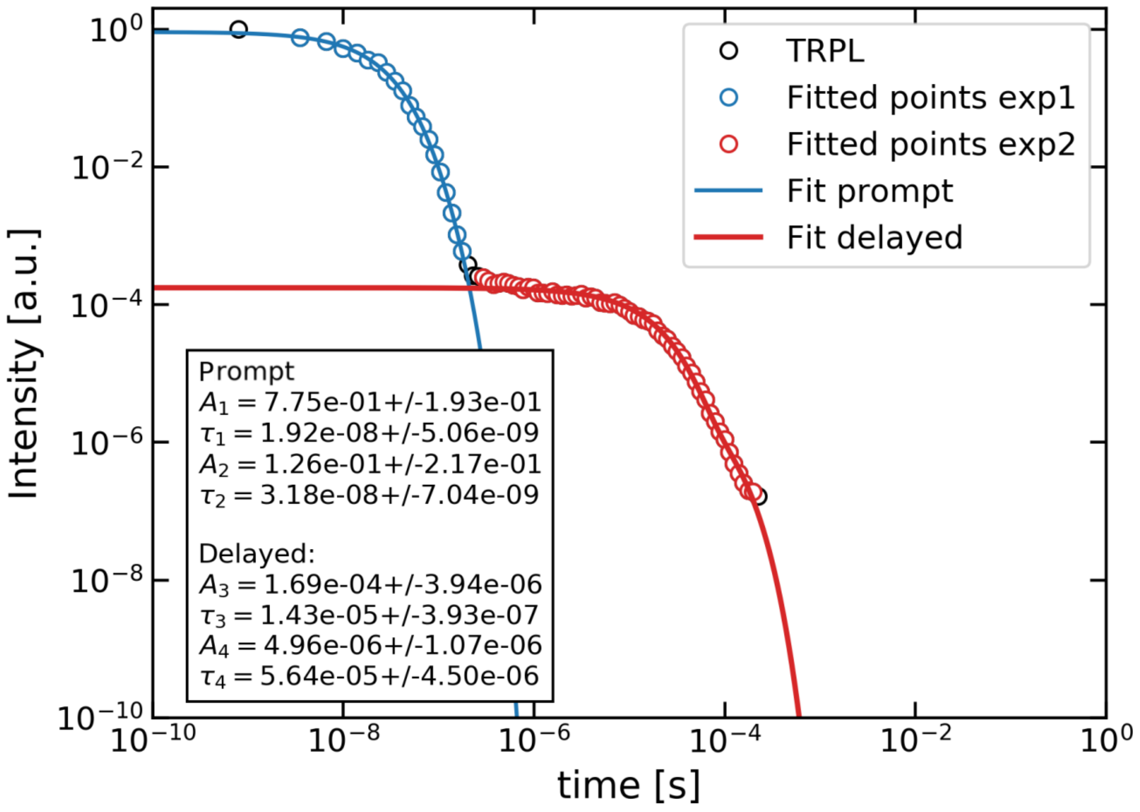

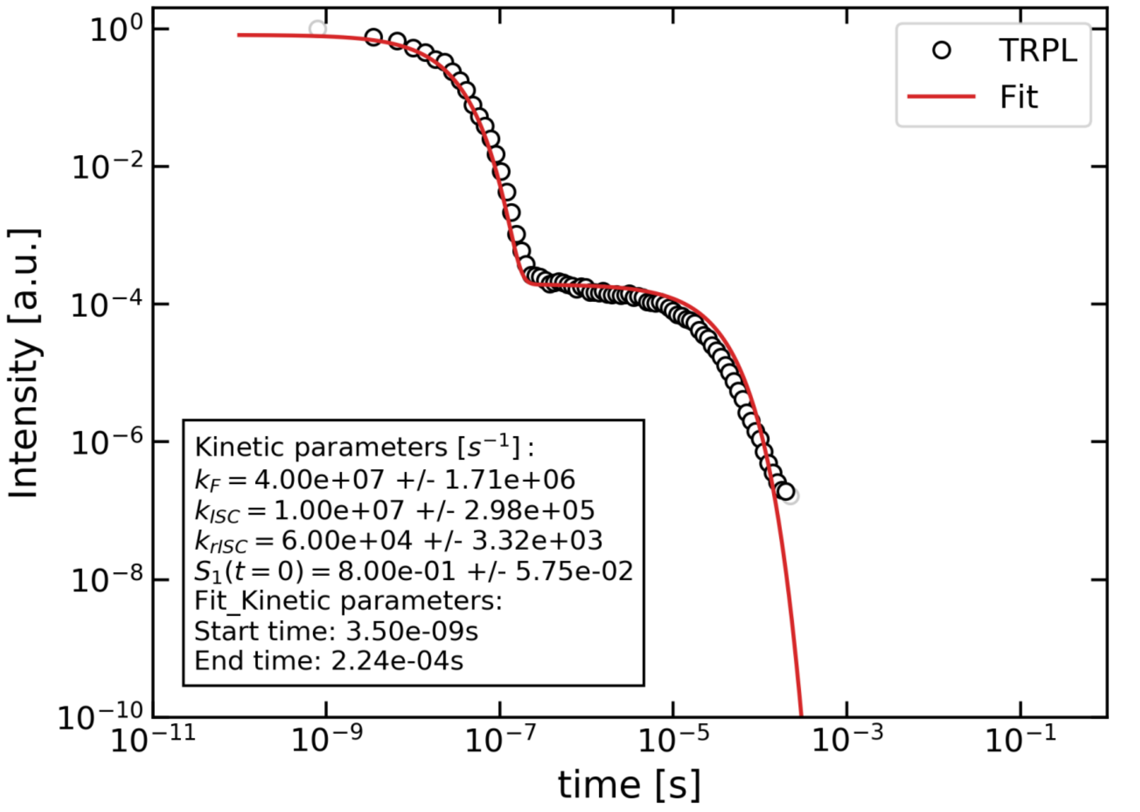

Figure S56. Emission decay fitting for **1b** in DCM (top biexponential, bottom kinetic)

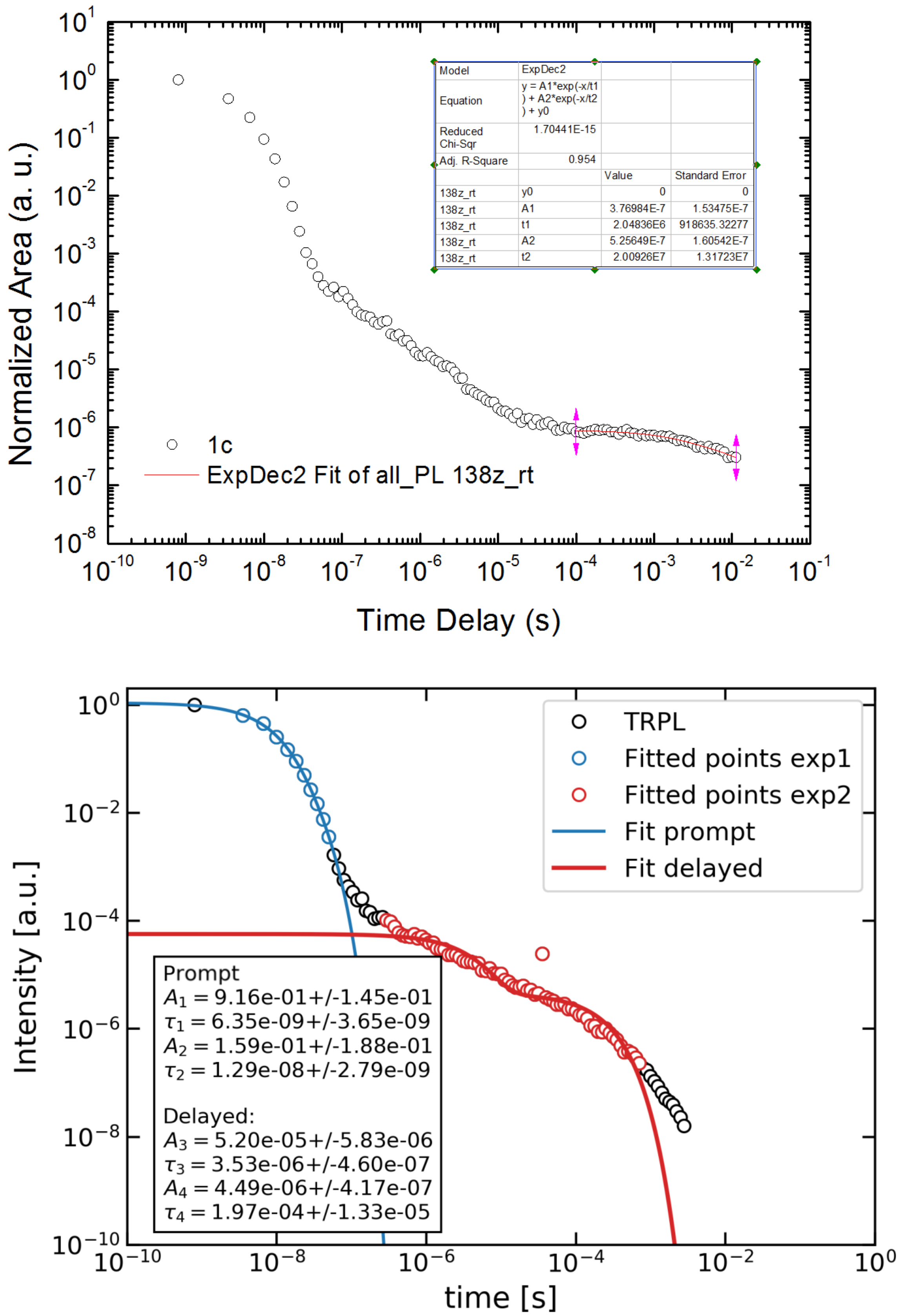

Figure S57. Emission decay fitting for **1c** in Zeonex (top) and neat film (bottom)

7. Cyclic Voltammetry

Table S5. Overview of the electrochemical analysis of compounds **1a-c** (performed in DMF (abs.), 0.1 M $TBAPF_6$, HOMO = -(IP+5.1) eV, LUMO = HOMO + Eg). Fc = $(C_5H_5)_2Fe$.

	IP (vs. Fc/Fc$^+$) [V]	EA (vs. Fc/Fc$^+$) [V]	Eg (V)	HOMO [eV]	LUMO [eV]
**1a**	0.38	-2.40	2.78	-5.48	-2.70
**1b**	0.40	-2.35	2.76	-5.50	-2.75
**1c**	0.39	-2.54	2.93	-5.49	-2.56

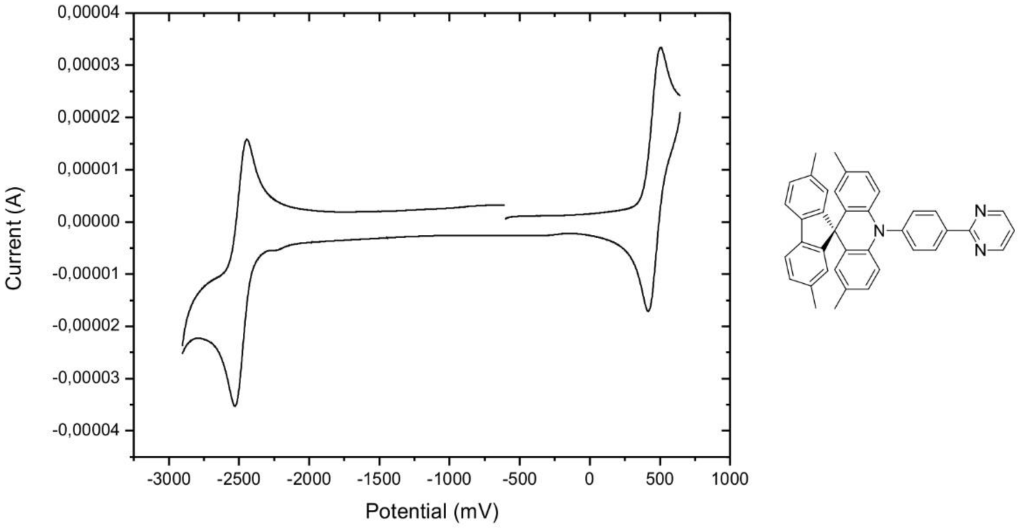

Figure S58. Cyclic voltammetry of compound **1a** (DMF (abs.), 0.1 M $TBAPF_6$, corrected vs. the ferrocene/ferrocenium couple Fc/Fc$^+$).

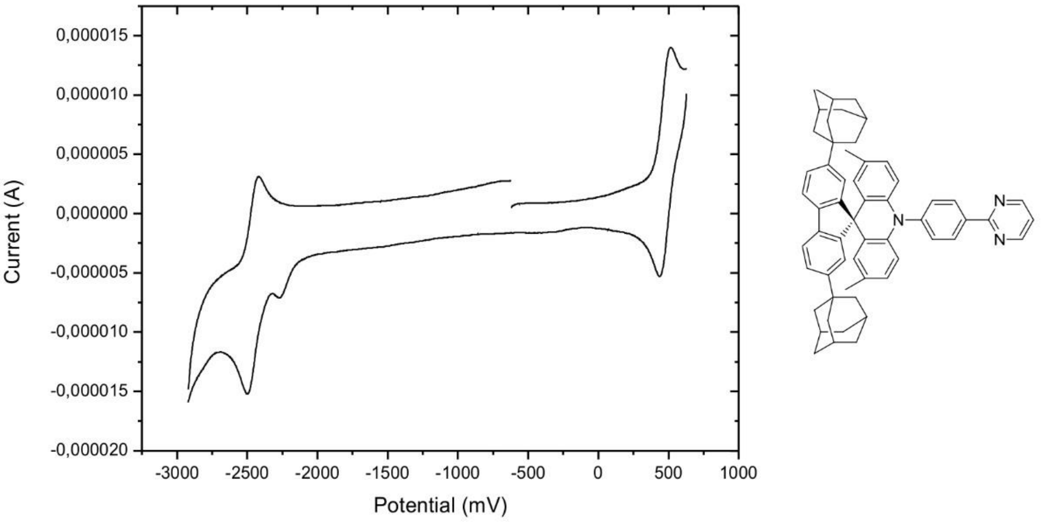

Figure S59. Cyclic voltammetry of compound **1b** (DMF (abs.), 0.1 M $TBAPF_6$, corrected vs. Fc/Fc$^+$).

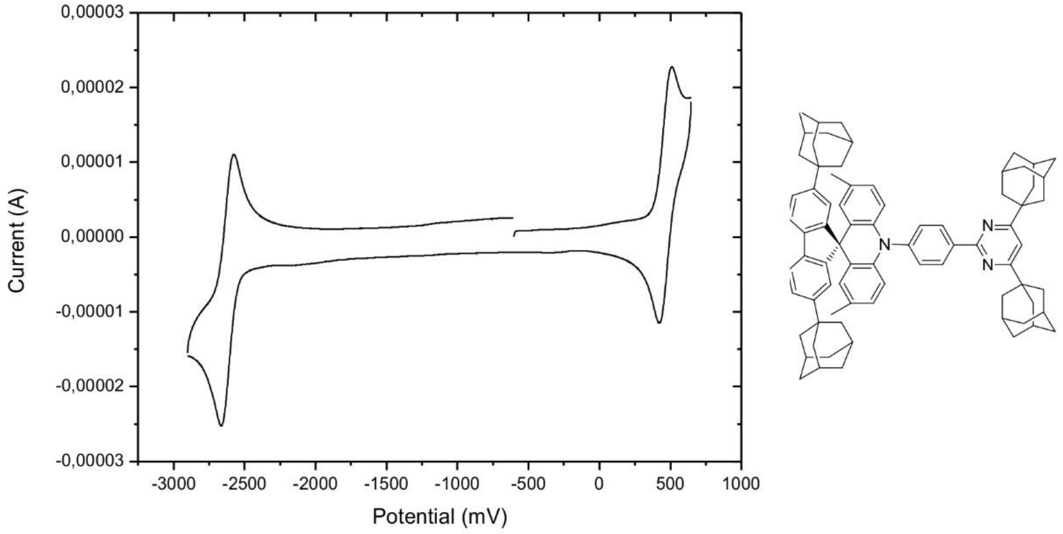

Figure S60. Cyclic voltammetry of compound **1c** (DMF (abs.), 0.1 M $TBAPF_6$, corrected vs. Fc/Fc^+).

8. References

1. Poriel, C.; Rault-Berthelot, J.; Barrière, F.; Slawin, A. M. Z., New Dispiro Compounds: Synthesis and Properties. *Org. Lett.* **2008,** *10* (3), 373-376.
2. Park, I. S.; Komiyama, H.; Yasuda, T., Pyrimidine-based twisted donor–acceptor delayed fluorescence molecules: a new universal platform for highly efficient blue electroluminescence. *Chem. Sci.* **2017,** *8* (2), 953-960.
3. Mai, W.-P.; Sun, B.; You, L.-Q.; Yang, L.-R.; Mao, P.; Yuan, J.-W.; Xiao, Y.-M.; Qu, L.-B., Silver catalysed decarboxylative alkylation and acylation of pyrimidines in aqueous media. *Org. Biomol. Chem.* **2015,** *13* (9), 2750-2755.
4. Eakins, G. L.; Alford, J. S.; Tiegs, B. J.; Breyfogle, B. E.; Stearman, C. J., Tuning HOMO–LUMO levels: trends leading to the design of 9-fluorenone scaffolds with predictable electronic and optoelectronic properties. *J. Phys. Org. Chem.* **2011,** *24* (11), 1119-1128.
5. Park, I. S.; Numata, M.; Adachi, C.; Yasuda, T., A Phenazaborin-Based High-Efficiency Blue Delayed Fluorescence Material. *Bull. Chem. Soc. Jpn.* **2016,** *89* (3), 375-377.
6. Neumann, T.; Benajiba, L.; Göring, S.; Stegmaier, K.; Schmidt, B., Evaluation of Improved Glycogen Synthase Kinase-3α Inhibitors in Models of Acute Myeloid Leukemia. *J. Med. Chem.* **2015,** *58* (22), 8907-8919.
7. Rees, B.; Jenner, L.; Yusupov, M., Bulk-solvent correction in large macromolecular structures. *Acta Crystallogr. D* **2005,** *61* (9), 1299-1301.
8. Frisch, M. J.; Trucks, G. W.; Schlegel, H. B.; Scuseria, G. E.; Robb, M. A.; Cheeseman, J. R.; Scalmani, G.; Barone, V.; Petersson, G. A.; Nakatsuji, H.; Li, X.; Caricato, M.; Marenich, A. V.; Bloino, J.; Janesko, B. G.; Gomperts, R.; Mennucci, B.; Hratchian, H. P.; Ortiz, J. V.; Izmaylov, A. F.; Sonnenberg, J. L.; Williams-Young, D.; Ding, F.; Lipparini, F.; Egidi, F.; Goings, J.; Peng, B.; Petrone, A.; Henderson, T.; Ranasinghe, D.; Zakrzewski, V. G.; Gao, J.; Rega, N.; Zheng, G.; Liang, W.; Hada, M.; Ehara, M.; Toyota, K.; Fukuda, R.; Hasegawa, J.; Ishida, M.; Nakajima, T.; Honda, Y.; Kitao, O.; Nakai, H.; Vreven, T.; Throssell, K.; Montgomery, J. A.; Jr.; Peralta, J. E.; Ogliaro, F.; Bearpark, M. J.; Heyd, J. J.; Brothers, E. N.; Kudin, K. N.; Staroverov, V. N.; Keith, T. A.; Kobayashi, R.; Normand, J.; Raghavachari, K.; Rendell, A. P.; Burant, J. C.; Iyengar, S. S.; Tomasi, J.; Cossi, M.; Millam, J. M.; Klene, M.; Adamo, C.; Cammi, R.; Ochterski, J. W.; Martin, R. L.; Morokuma, K.; Farkas, O.; Foresman, J. B.; Fox, D. J. *Gaussian 16, Revision B.01*, Gaussian 16, Revision B.01, Gaussian Inc.: Wallingford CT, 2016.

9. Becke, A. D., Density-functional thermochemistry. III. The role of exact exchange. *J. Chem. Phys.* **1993,** *98* (7), 5648-5652.
10. Lee, C.; Yang, W.; Parr, R. G., Development of the Colle-Salvetti correlation-energy formula into a functional of the electron density. *Phys. Rev. B* **1988,** *37* (2), 785-789.
11. Yanai, T.; Tew, D. P.; Handy, N. C., A new hybrid exchange–correlation functional using the Coulomb-attenuating method (CAM-B3LYP). *Chem. Phys. Lett.* **2004,** *393* (1), 51-57.
12. Boese, A. D.; Martin, J. M. L., Development of density functionals for thermochemical kinetics. *J. Chem. Phys.* **2004,** *121* (8), 3405-3416.
13. Petersson, G. A.; Bennett, A.; Tensfeldt, T. G.; Al-Laham, M. A.; Shirley, W. A.; Mantzaris, J., A complete basis set model chemistry. I. The total energies of closed-shell atoms and hydrides of the first-row elements. *J. Chem. Phys.* **1988,** *89* (4), 2193-2218.
14. Petersson, G. A.; Al-Laham, M. A., A complete basis set model chemistry. II. Open-shell systems and the total energies of the first-row atoms. *J. Chem. Phys.* **1991,** *94* (9), 6081-6090.
15. Dolomanov, O. V.; Bourhis, L. J.; Gildea, R. J.; Howard, J. A. K.; Puschmann, H., OLEX2: a complete structure solution, refinement and analysis program. *J. Appl. Crystallogr.* **2009,** *42* (2), 339-341.
16. Dreuw, A.; Weisman, J. L.; Head-Gordon, M., Long-range charge-transfer excited states in time-dependent density functional theory require non-local exchange. *J. Chem. Phys.* **2003,** *119* (6), 2943-2946.
17. Reimers, J. R.; Rätsep, M.; Freiberg, A., Asymmetry in the Q (y) Fluorescence and Absorption Spectra of Chlorophyll a Pertaining to Exciton Dynamics. *Front Chem* **2020,** *8*, 588289.
18. Shkoor, M.; Mehanna, H.; Shabana, A.; Farhat, T.; Bani-Yaseen, A. D., Experimental and DFT/TD-DFT computational investigations of the solvent effect on the spectral properties of nitro substituted pyridino[3,4-c]coumarins. *J. Mol. Liq.* **2020,** *313*, 113509.
19. Basha, M. T.; Alghanmi, R. M.; Soliman, S. M.; Alharby, W. J., Synthesis, spectroscopic, thermal, structural characterization and DFT/TD-DFT computational studies for charge transfer complexes of 2,4-diamino pyrimidine with some benzoquinone acceptors. *J. Mol. Liq.* **2020,** *309*, 113210.
20. Promkatkaew, M.; Suramitr, S.; Karpkird, T.; Ehara, M.; Hannongbua, S., DFT/TD-DFT investigation on the photoinduced electron transfer of diruthenium and viologen complexes. *J. Lumin.* **2020,** *222*, 117121.
21. Divya, V. V.; Suresh, C. H., Density functional theory study on the donating strength of donor systems in dye-sensitized solar cells. *New J. Chem.* **2020,** *44* (17), 7200-7209.
22. Sun, H.; Zhong, C.; Brédas, J.-L., Reliable Prediction with Tuned Range-Separated Functionals of the Singlet–Triplet Gap in Organic Emitters for Thermally Activated Delayed Fluorescence. *J. Chem. Theory Comput.* **2015,** *11* (8), 3851-3858.
23. Tawada, Y.; Tsuneda, T.; Yanagisawa, S.; Yanai, T.; Hirao, K., A long-range-corrected time-dependent density functional theory. *J. Chem. Phys.* **2004,** *120* (18), 8425-8433.
24. Peach, M. J. G.; Cohen, A. J.; Tozer, D. J., Influence of Coulomb-attenuation on exchange–correlation functional quality. *Phys. Chem. Chem. Phys.* **2006,** *8* (39), 4543-4549.
25. Mandal, I.; Manna, S.; Venkatramani, R., UV–Visible Lysine–Glutamate Dimer Excitations in Protein Charge Transfer Spectra: TDDFT Descriptions Using an Optimally Tuned CAM-B3LYP Functional. *J. Phys. Chem. B* **2019,** *123* (51), 10967-10979.
26. Ward, J. S.; Danos, A.; Stachelek, P.; Fox, M. A.; Batsanov, A. S.; Monkman, A. P.; Bryce, M. R., Exploiting trifluoromethyl substituents for tuning orbital character of singlet and triplet states to increase the rate of thermally activated delayed fluorescence. *Mater. Chem. Front.* **2020,** *4* (12), 3602-3615.
27. Hempe, M.; Harrison, A. K.; Ward, J. S.; Batsanov, A. S.; Fox, M. A.; Dias, F. B.; Bryce, M. R., Cyclophane Molecules Exhibiting Thermally Activated Delayed Fluorescence: Linking Donor Units to Influence Molecular Conformation. *J. Org. Chem.* **2021,** *86* (1), 429-445.
28. Shao, Y.; Mei, Y.; Sundholm, D.; Kaila, V. R. I., Benchmarking the Performance of Time-Dependent Density Functional Theory Methods on Biochromophores. *J. Chem. Theory Comput.* **2020,** *16* (1), 587-600.

29. Samanta, P. K.; Kim, D.; Coropceanu, V.; Brédas, J.-L., Up-Conversion Intersystem Crossing Rates in Organic Emitters for Thermally Activated Delayed Fluorescence: Impact of the Nature of Singlet vs Triplet Excited States. *J. Am. Chem. Soc.* **2017,** *139* (11), 4042-4051.
30. Improta, R.; Barone, V.; Scalmani, G.; Frisch, M. J., A state-specific polarizable continuum model time dependent density functional theory method for excited state calculations in solution. *J. Chem. Phys.* **2006,** *125* (5), 054103.
31. Penfold, T. J.; Dias, F. B.; Monkman, A. P., The theory of thermally activated delayed fluorescence for organic light emitting diodes. *Chem. Comm.* **2018,** *54* (32), 3926-3935.
32. Allouche, A.-R., Gabedit—A graphical user interface for computational chemistry softwares. *J. Comput. Chem.* **2011,** *32* (1), 174-182.
33. O'boyle, N. M.; Tenderholt, A. L.; Langner, K. M., cclib: A library for package-independent computational chemistry algorithms. *J. Comput. Chem.* **2008,** *29* (5), 839-845.
34. Neese, F.; Aravena, D.; Atanasov, M.; Auer, A. A.; Becker, U.; Bistoni, G.; Brehm, M.; Bykov, D.; Chilkuri, V. G.; Datta, D.; Dutta, A. K.; Ganyushin, D.; Garcia, M.; Guo, Y.; Hansen, A.; Helmich-Paris, B.; Huntington, L.; Izsak, R.; Kollmar, C.; Kossmann, S.; Krupicka, M.; Lang, L.; Lenk, D.; Liakos, D.; Manganas, D.; Pantazis, D.; Petrenko, T.; Pinski, P.; Reimann, C.; Retegan, M.; Riplinger, C.; Risthaus, T.; Roemelt, M.; Saitow, M.; Sandhofer, B.; Sen, A.; Sivalingam, K.; de Souza, B.; Stoychev, G.; Van den Heuvel, W.; Wezisla, B.; Wennmohs, F. *Orca version 4.1.1*, Department of Theory and Spectroscopy, Max Planck Institute fuer Kohlenforschung, Kaiser Wilhelm Platz 1, D-45470 Muelheim/Ruhr, Germany: 2019.
